# Examining the effects of additives and precursors on the reactivity of rhodium alkyl nitrenes generated from substituted hydroxylamines

**DOI:** 10.3389/fchem.2023.1271896

**Published:** 2023-11-03

**Authors:** Hidetoshi Noda, Yasuko Asada, Masakatsu Shibasaki

**Affiliations:** Institute of Microbial Chemistry (BIKAKEN), Tokyo, Japan

**Keywords:** nitrene, N-heterocycle, rhodium, C-H insertion, kinetic isotope effect

## Abstract

In this study, the reactivity of the alkyl nitrenes, generated from the substituted hydroxylamine precursors, was determined using the same rhodium catalyst. The results revealed that in competitive C–H insertion experiments, the regioselectivity between benzylic and tertiary C–H bonds could be modulated by adding Brønsted acids or changing the substituents on oxygen. This study enhances our understanding of the metallonitrene structures and provides valuable insights for further development of selective N-heterocycle syntheses.

## 1 Introduction

Nitrogen-containing compounds prevail over biologically active compounds (Lovering et al., 2009; Vitaku et al., 2014). Therefore, the synthetic chemists have made considerable efforts to introduce nitrogen atoms at desired positions in the molecular skeleton (Park et al., 2017; Trowbridge et al., 2020). Among various approaches, utilizing nitrenes is preferred, as they can functionalize the otherwise inert C–H bonds (Müller and Fruit, 2003; Díaz-Requejo and Pérez, 2008; Darses et al., 2017). Given the high reactivity of free nitrenes, metallonitrenes are primarily used for nitrogen insertion as their reactivity can be regulated by the structure of the metal complexes (Ju and Schomaker, 2021). Metalated nitrenes are typically generated from oxidized precursors, either prepared *in situ* or from those containing a labile bond (Breslow and Gellman, 1982; Nägeli et al., 1997; Lebel et al., 2005).

The substituents on the nitrogen can be used to classify nitrene structures such as carbamoyl (Cui and He, 2004), sulfamoyl (Espino et al., 2001), aryl (Stokes et al., 2007), acyl (Hong et al., 2018), and alkyl (Hennessy and Betley, 2013) nitrenes. The class of nitrenes determines the structure of the resulting product. For instance, intramolecular C–H insertion of sulfamoyl nitrenes provides a 1,3-aminoalcohol unit, whereas that of alkyl nitrenes delivers a saturated N-heterocycle. Therefore, the advancement in nitrene chemistry is directed toward expanding the product structures and its evolution has resulted in the development of efficient catalysts and new precursors (Roizen et al., 2012; Alderson et al., 2017; Hong et al., 2021). The chemoselective amination reactions have garnered considerable interest in this area that has triggered the identification of various catalyst-controlled aminations (Noda et al., 2021). However, there are limited studies in the literature investigating the reactivity difference between various nitrene classes using identical catalysts. The comparison of the same nitrene class obtained from different precursors is also lacking. This could be attributed to the lack of a suitable system for studying the reactivities.

**SCHEME 1:**
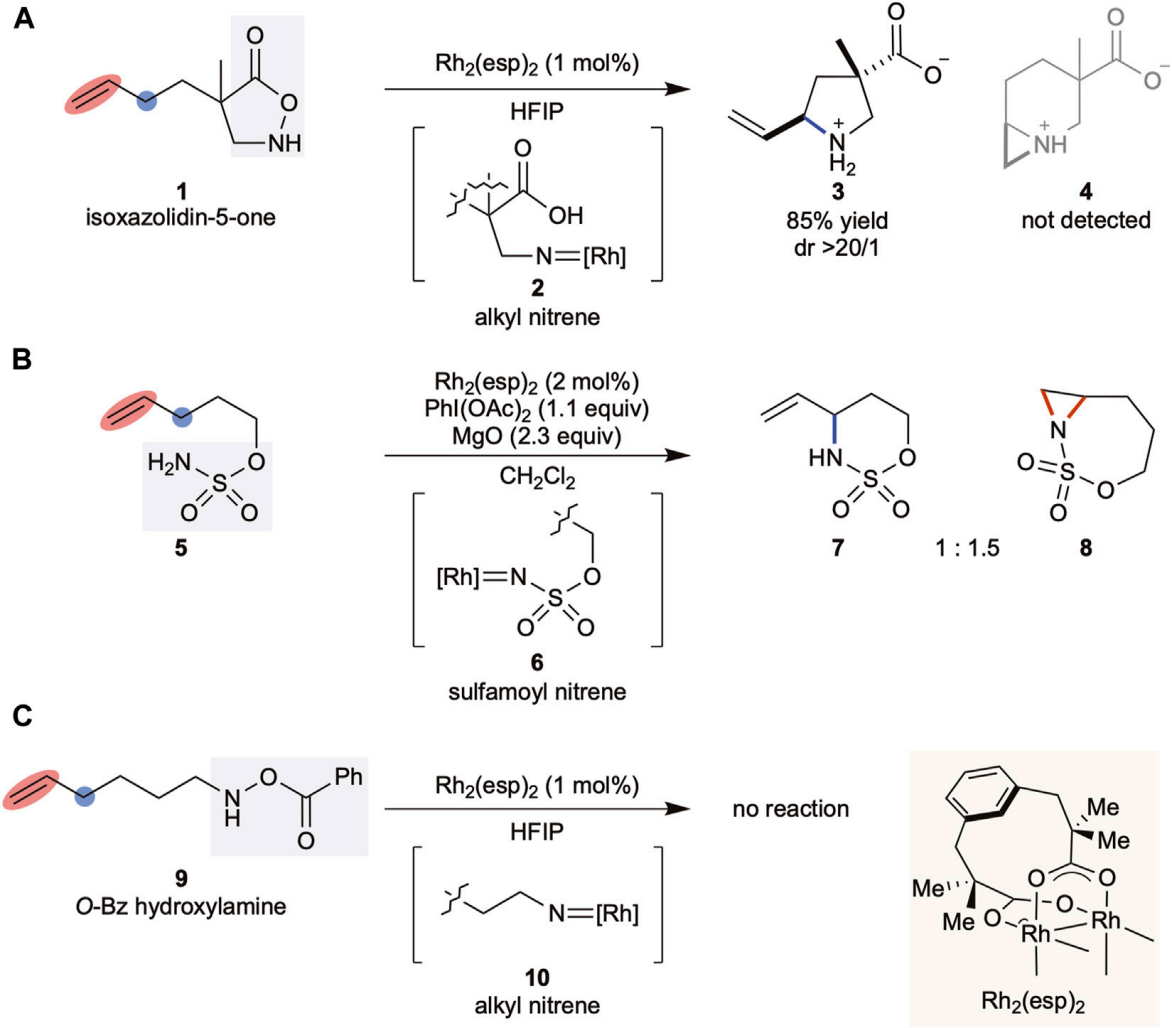
**(A–C)** Background of this work. esp: α, α, α′, α′-tetramethyl-1,3-benzenedipropionic acid, HFIP: 1,1,1,3,3,3-hexafluoroisopropanol.

We previously reported that substituted isoxazolidin-5-ones (Annibaletto et al., 2017; Noda, 2021) acted as alkyl nitrene precursors in the presence of rhodium (Yu et al., 2019) or copper catalysts (Tak et al., 2021a). The generated metallonitrene reacted intramolecularly with an aromatic ring (Tak et al., 2021b) or C(sp^3^)–H bond (Espinosa et al., 2019) to afford the corresponding unprotected cyclic β-amino acids. In our study to synthesize remotely decorated trisubstituted pyrrolidines via C(sp^3^)–H insertion (Tang et al., 2022) using Rh_2_(esp)_2_, a Du Bois catalyst (Espino et al., 2004), it was observed that the alkyl nitrene derived from the heterocycle selectively aminated the C(sp^3^)–H bond at the allylic position without touching the double bond (Scheme 1A), which was in contrast to the sulfamoyl nitrene favoring aziridination over C(sp^3^)–H insertion, using the same rhodium catalyst (Scheme 1B) (Fiori et al., 2009). These results highlighted the unique nature of the nitrene reactivities associated with their structural classes.

**SCHEME 2:**
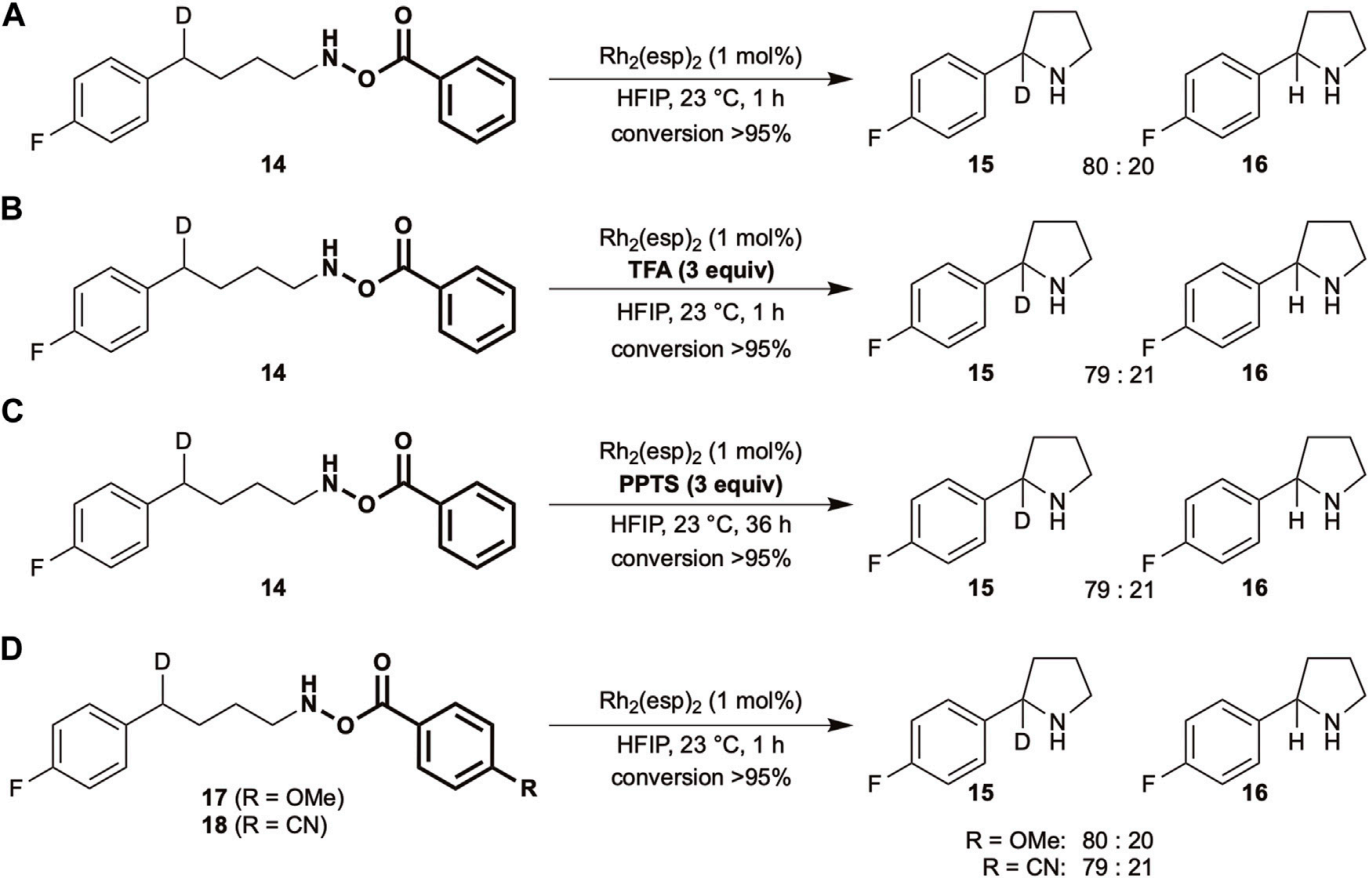
KIE experiments using Rh_2_(esp)_2_ as a catalyst under various conditions.

Driven by the significance of saturated N-heterocycles in drug discovery programs, we further investigated alkyl nitrenes and identified *O*-benzoyl hydroxylamines as efficient alkyl nitrene precursors for the transformation of a linear primary amine into the corresponding five-membered cyclic amine (Noda et al., 2020). When we subjected substrate **9** to the catalytic conditions to explore the scope of the method, no reaction was observed, resulting in full recovery of the substrate (Scheme 1C). As both substrates **1** and **9** were expected to generate similar alkyl rhodium nitrene species **2** and **10**, respectively, as shown in Scheme 2, this difference in the outcomes could be attributed to the precursor structure. However, the lack of insight into the structure-reactivity relationship between nitrene precursors and the reaction conditions required a further detailed examination of these factors. Herein, we report our study on the reactivity of alkyl nitrenes derived from substituted hydroxylamines.

## 2 Results

From the outset, we focused on the reactivities of alkyl nitrenes, as the products obtained from the intramolecular amination are medicinally important saturated N-heterocycles. In addition to isoxazolidin-5-ones and *O*-Bz hydroxylamines, alkyl azides (Thacker et al., 2016; Bagh et al., 2017; Shing et al., 2018; Qin et al., 2019), and *O*-Ts hydroxylamines (Munnuri et al., 2017) act as nitrene precursors. Owing to their stability and facile structural modification, *O*-Bz hydroxylamines were used as model substrates in this study, and Rh_2_(esp)_2_ was used as the catalyst. Examination of the presumed nitrene structures **2** and **10** implied that a suitably located acidic proton in **2** played an important role in determining the reactivity, which was the driving force to investigate the additive effect using Brønsted acids and bases.

Table 1 summarizes the influence of additives on the regioselectivity of reactions, where the selectivity between benzylic and tertiary C–H bonds was used as a reactivity probe. In the absence of additives, the site selectivity of **11aA** is close to 1:4, favoring the tertiary C–H bond (entry 1). The ratio marginally decreases in the presence of trifluoroacetic acid (TFA) (entry 2), suggesting that an acidic proton source plays a vital role in the selectivity-determining transition state. Higher acid loadings do not decrease the selectivity further (entry 3). It was observed that all the acids do not lower the selectivity, and the addition of a stronger acid, trifluoromethanesulfonic acid (TfOH), creates a strong bias in the reaction site for the tertiary C–H bond, although with a considerably slower kinetics (entry 4). Similar trend is observed for pyridinium *p*-toluenesulfonate (PPTS), which is a milder Brønsted acid compared to the TFA (entry 5). In contrast to acidic additives, the addition of a Brønsted base does not lead to a noticeable shift in the regioselectivity (entries 6–8).

**TABLE 1:** Evaluation of additives on the regioselectivity of Rh-alkyl nitrene.

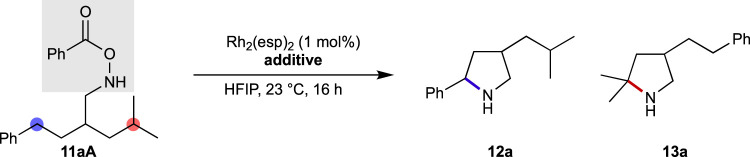			

Entry	Additive (x equiv)	Conversion (%)^a^	**12a:13a** ^b^
1	—	>95	21:79
2	TFA (3)	>95	29:71
3	TFA (10)	>95	31:69
4^c^	TfOH (3)	12 (21)	4:>96 (4: > 96)
5	PPTS (3)	27	4:>96
6	2,6-Lutidine (3)	>95	24:76
7	Et_3_N (3)	>95	26:74
8	Cs_2_CO_3_ (3)	>95	19:81
9	Barton’s base (3)	Complex mixture	

^a^ Conversion was determined using 1H NMR, analysis of unpurified reaction mixture.

^b^ The regioselectivity was determined using 1H NMR, and reverse-phase HPLC, analysis of unpurified reaction mixture.

^c^ The values in parentheses were obtained after 48 h. TFA, trifluoroacetic acid; TfOH, trifluoromethanesulfonic acid; PPTS, pyridinium *p*-toluenesulfonate.

A change in the selectivity is often accompanied by a change in the reaction mechanism. Therefore, to understand the nature of reactive intermediates under Brønsted acidic conditions, kinetic isotope effect (KIE) experiments were conducted (Scheme 2). Fluorine-substituted compounds were used for this purpose as the high sensitivity of ^19^F nuclei in nuclear magnetic resonance (NMR) is beneficial for determining the selectivity. Under standard conditions, using 1 mol% Rh_2_(esp)_2_ as a catalyst in 1,1,1,3,3,3-hexafluoroisopropanol (HFIP) at ambient temperature, a KIE value of 4.0 is obtained (Scheme 2A). The value remains the same in the presence of TFA (Scheme 2B), suggesting similar properties of the reactive intermediate in both cases. Despite the distinct selectivity trend observed in Table 1, the addition of PPTS does not change the KIE value (Scheme 2C). The KIE value of a similar *N*-Boc-*O*-Ts substrate was reported to be 5.3 (Munnuri et al., 2017), therefore, electronically tuned benzoate substrates were subjected to identical conditions. The obtained values are approximately same to those of the unmodified substrate (Scheme 2D).

We questioned whether the difference in the KIE values between *O*-Bz and *O*-Ts hydroxylamines would be translated into a difference in regioselectivities. Although the reactivity trend of *O*-Ts hydroxylamines has been previously studied using a different rhodium catalyst, a comparison with three classes of substrates was carried out for ranking the reactivities of various C–H bonds (benzylic, tertiary, secondary, and primary). In addition to regioselectivity, diastereoselectivity was utilized as the other reactivity probe.

The instability of the *N*-H form of *O*-Ts hydroxylamines requires *in situ* removal of a protective group from the nitrogen. Following the previous reports, the *N*-Boc group was used to mask the nitrogen atom and TFA was used as the proton source in TFE. Thus, TFA was included in the reactions with *O*-Bz substrates for a fair comparison (Table 2). All the products were isolated after conversion to their *N*-Ts forms. Examining the regioselectivity between benzylic and tertiary C–H bonds, a marginally lower selectivity is obtained using the *O*-Ts substrate compared to that of *O*-Bz substrate, although the diastereoselectivity is similar for both (entries 1 and 2). Owing to their diminished reactivity toward metallonitrene, cyclization products are not obtained at the secondary or primary C–H bonds for either of the substrates. However, better diastereoselectivities are recorded with *O*-Ts substrates compared to those with *O*-Bz substrates (entries 3 vs. 4 and 5 vs. 6).

**TABLE 2:** Comparison between *O*-Bz and *O*-Ts hydroxylamines using rhodium catalyst.^a^

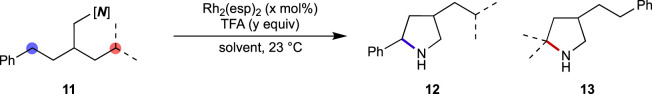					

Entry	**11**	Conditions	Yield (%)	**12:13**	*anti*/*syn* (**12**)
1	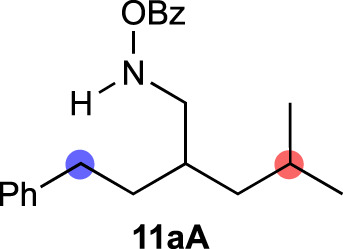	A	73	31:69	83/17
2	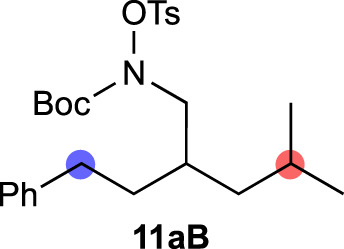	B	77	43:57	84/16
3	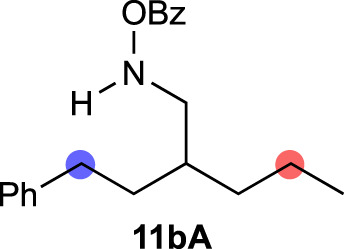	A	83	>96:4	81/19
4	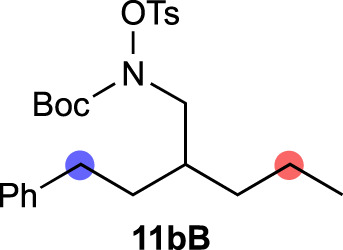	B	84	>96:4	88/12
5	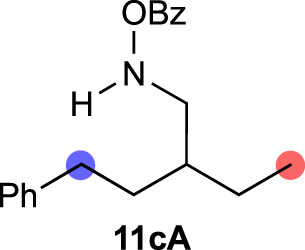	A	82	>96:4	83/17
6	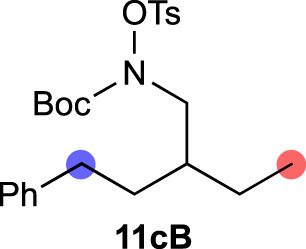	B	83	>96:4	89/11

Conditions A: Rh_2_(esp)_2_ (1 mol%), TFA (10 equiv), HFIP., Conditions B: Rh_2_(esp)_2_ (2 mol%), TFA (2 equiv), TFE.

^a^ Products were isolated after conversion into the corresponding *N*-Ts adducts. TFE; 2,2,2-trifluoroethanol, Ts; *p*-toluenesulfonyl.

## 3 Discussion

**SCHEME 3:**
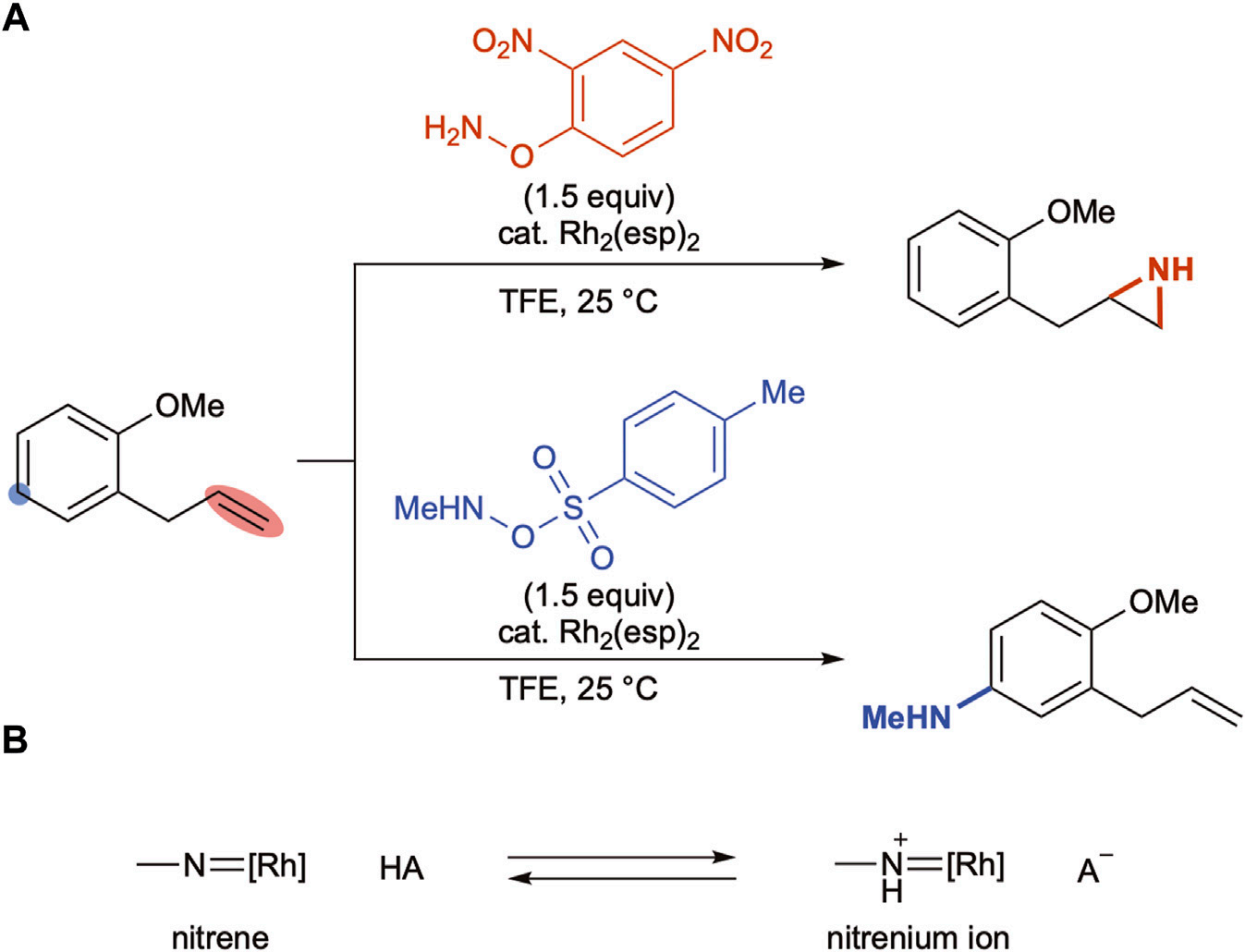
**(A)** Reagent-controlled chemoselectivity switch under rhodium-catalyzed conditions. **(B)** Possible interconversion between nitrenes and nitrenium ions.

Falck and coworkers reported aziridination of olefins with *O*-(2,4-dinitrophenyl)hydroxylamine under the influence of a Rh_2_(esp)_2_ catalyst (Jat et al., 2014). The group revealed in a subsequent report that the combination of the same rhodium catalyst with *O*-Ts hydroxylamine aminated an aromatic C–H bond keeping the olefin moiety intact (Scheme 3A) (Paudyal et al., 2016). The authors proposed that the rhodium nitrene generated upon N–O bond cleavage was in equilibrium with the protonated nitrenium ion and the two species exhibited distinct reactivity, which justified the remarkable chemoselectivity switch (Scheme 3B). Moreover, acidity of the liberated Brønsted acids determined the equilibrium positions; the nitrophenol was not strong enough to protonate metallonitrenes, whereas a stronger sulfonic acid propelled the equilibrium forward to yield nitrenium ions.

Given that the acidity of the reaction medium is responsible for the equilibrium position, it was hypothesized that the addition of external Brønsted acids should play an identical role. The results in Table 1 reveal that the inclusion of sulfonic acids drastically changes the regioselectivity trend (entries 4,5, Table 1), which can be explained by a possible shift in the equilibrium. The observed lower reactivity could be ascribed to either the lower C–H insertion activity of the nitrenium ion intermediate or the slower N–O bond cleavage to form a reactive intermediate. However, the addition of external Brønsted acids does not alter the KIE values (Scheme 2A–C), suggesting that a similar reactive intermediate is involved in the C–H amination step in the absence or presence of acids.

Over the years, KIE experiments have been used to assess and elucidate the nature of reactive intermediates in various C–H functionalization reactions. It is well-known in nitrene chemistry that KIE values lower than three indicate the presence of a concerted mechanism involving a singlet nitrene. For instance, the KIE values of sulfamoyl nitrenes using a similar rhodium catalyst were in the range of 1.9–2.9 (Fiori et al., 2009; Varela-Álvarez et al., 2016). In contrast, a stepwise mechanism displays higher KIE values (Badiei et al., 2008; Harvey et al., 2011). The difference in KIE values between *O*-Bz (4.0) and *O*-Ts (5.3) hydroxylamines using the same catalyst inferred that precursor structures play a significant role in determining the nature of reactive intermediate but the acidity of the reaction medium. As a slight modification of the benzoic acid derivatives (pKa in H_2_O: *p*-CN-C_6_H_4_ 3.55, C_6_H_5_ 4.21, *p*-MeO-C_6_H_4_ 4.47) did not affect the KIE values (Scheme 2D), a considerably drastic change in the acidity of the leaving groups might be required to induce a change.

**FIGURE 1:**
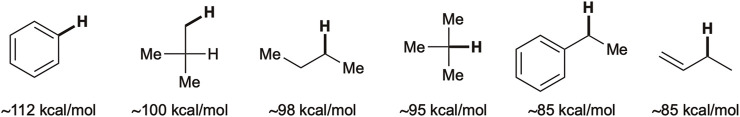
BDEs for various representative C–H bonds.

The precursor structure-dependent KIE value implies that the reactive intermediate generated from *O*-Ts substrates possesses a triplet, radical-like nature. Radical-like intermediates typically follow the bond dissociation energy (BDE) order, which is the enthalpy change associated with the homolytic scission of the bond (Figure 1). The results summarized in Table 2 agree with this trend. Therefore, the *O*-Ts substrate undergoes the benzylic amination preferably compared to that for *O*-Bz (entry 1 vs. 2, Table 2). Comparable results are obtained in HFIP using **11aB** (76% yield, **12a**:**13a** 41:59, *anti*/*syn* 83/17), excluding the possibility that the choice of solvent governs the selectivity. The observed diastereoselectivities support the different natures of the reactive intermediates generated from *O*-Bz and *O*-Ts hydroxylamines.

## 4 Conclusion

We have investigated the reactivity of rhodium alkyl nitrenes generated from substituted hydroxylamines. The addition of Brønsted acids modulated the regioselectivity of the intramolecular C–H insertion between the benzylic and tertiary positions. Despite the distinct regioselectivities, approximately identical KIE values were observed for various Brønsted acids. In contrast to external acids, the KIE values fluctuated as a function of the precursor structures. The conditions that produced the more radical-like reactive intermediate followed the expected reaction tendency. Although further efforts are required to completely understand the nature of reactive intermediates, particularly with the external addition of Brønsted acids, our results comprehensively confirmed that the reactivities of seemingly similar reactive intermediates could be regulated by incorporating additives or changing the precursor structures. This work significantly enhances our understanding of the rhodium nitrene structures, which are typically devoid of precursor residues, and opens up new avenues for a substrate-controlled approach to fine-tune the reactivity, as with other hydroxylamine-involving reactions (Noda et al., 2014; Niu and Buchwald, 2015).

## Data Availability

The original contributions presented in the study are included in the article/Supplementary Material, further inquiries can be directed to the corresponding authors.
