# Supplementary material for: Examining the effects of additives and precursors on the reactivity of rhodium alkyl nitrenes generated from substituted hydroxylamines
Source: Front Chem. 2023 Nov 3;11:1271896. doi: 10.3389/fchem.2023.1271896 (PMC10654751; doi:10.3389/fchem.2023.1271896)

Reactivity of Rhodium Alkyl Nitrenes Generated from Substituted Hydroxylamines

Hidetoshi Noda,* Yasuko Asada and Masakatsu Shibasaki*

Institute of Microbial Chemistry (BIKAKEN), Tokyo, Japan

Email: hnoda@bikaken.or.jp; mshibasa@bikaken.or.jp

Table of Contents

1. General information S2
   1-1. Reactions and purifications S2
   1-2. Characterizations S2
   1-3. Solvents and reagents S2

2. Synthesis of substrates S3

3. Rh-catalyzed C–H functionalization S9

4. References S10

5. NMR Spectra S11

1. General information

1-1. Reactions and purifications

Unless otherwise noted, all reactions were carried out under an air atmosphere and were stirred with Teflon-coated magnetically stirred bars. All work-up and purification procedures were carried out with reagent-grade solvents under ambient atmosphere. Thin layer chromatography (TLC) was performed on Merck TLC plates (0.25 mm) pre-coated with silica gel 60 F254 and visualized by UV quenching and staining with ninhydrin or $KMnO_4$. Normal phase flash column chromatography was performed on a Biotage Isolera Spektra One.

1-2. Characterizations

Infrared (IR) spectra were recorded on a HORIBA FT210 Fourier transform infrared spectrophotometer. NMR spectra were recorded on a Bruker AVANCE III HD400 NMR spectrometers. Chemical shifts (δ) are given in ppm relative to residual solvent peaks.[1] Data for ^{1}H NMR are reported as follows: chemical shift (multiplicity, coupling constants where applicable, number of hydrogens). Abbreviations are as follows: s (singlet), d (doublet), t (triplet), dd (doublet of doublet), dt (doublet of triplet), ddd (doublet of doublet of doublet), q (quartet), m (multiplet), br (broad). High-resolution mass spectra (ESI TOF (+)) were measured on a Thermo Fisher Scientific LTQ Orbitrap XL.

1-3. Solvents and reagents

Unless otherwise noted, materials were purchased from commercial suppliers (TCI, Merck-Sigma-Aldrich, Combi-Blocks, Enamine, BLD Pharmatech) and were used without further purification. Anhydrous MeOH were purchased from commercial suppliers. THF, CH_2Cl_2, and CH_3CN were purified by passing through a solvent purification system (Glass Contour). TFE was purchased from TCI, and used as received. HFIP and $Rh_2(esp)_2$ were purchased from Sigma-Aldrich, and used as received.

2. Synthesis of substrates

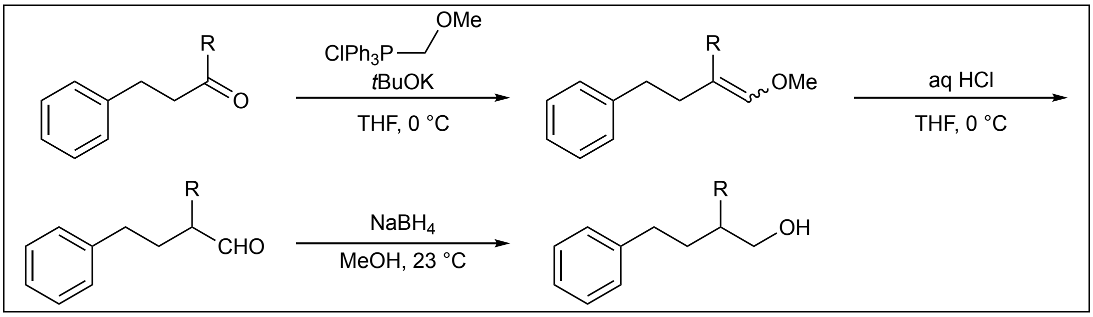

General procedure A: To a suspension of phosphonium chloride (1.1 equiv) in THF (0.2 M) was added tBuOK (1.0 M in THF, 1.06 equiv) at 0 °C. The solution was stirred for 1 h and ketone (1.0 equiv) was added. After the addition of sat aq NH_4Cl, the aqueous phase was extracted with EtOAc (x3). The combined organic layers were washed with brine, dried over Na_2SO_4, and removed under under reduced pressure to give a crude material.

The crude vinyl ether was dissolved in THF, followed by the addition of aq HCl (total volume ratio of conc HCl/H_2O/THF = 1/1/4, final concentration was 0.1 M). The solution was stirred for 1 h at 0 °C. After the addition of sat aq $NaHCO_3$, the aqueous phase was extracted with EtOAc (x3). The combined organic phase was washed with brine, dried over Na_2SO_4, and removed under reduced pressure. The obtained aldehyde was somewhat unstable and used immediately in the next step.

To a solution of crude aldehyde in MeOH was added $NaBH_4$ (1.0 equiv). The solution was stirred at 23 °C until the aldehyde was fully consumed (typically within 1 h). After the addition of 1M aq HCl, MeOH was removed under reduced pressure. The aqueous phase was extracted with EtOAc (x3). The combined organic phase was washed with brine, dried over Na_2SO_4, and removed. The resulting crude material was purified by silica gel column chromatography, eluting with hexane/EtOAc to afford primary alcohol.

4-Methyl-2-phenethylpentan-1-ol (S1): Prepared by the general procedure A from

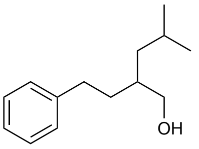

5-methyl-1-phenylhexan-3-one (3.44 g, 18.1 mmol), and isolated as a colorless oil (2.34 g, 78% yield for three steps). **IR** (thin film): *ν* 3328, 2953, 2924, 2867, 1454, 1384, 1366, 1029 cm^{-1}; **^{1}H NMR** (400 MHz, $CDCl_3$): δ 7.33–7.24 (m, 2H), 7.23–7.14 (m, 3H), 3.64–3.54 (m, 2H), 2.72–2.58 (m, 2H), 1.78–1.54 (m, 4H), 1.34 (brs, 1H), 1.29–1.13 (m, 2H), 0.89 (t, *J* = 6.7 Hz, 6H); **^{13}C NMR** (100 MHz, $CDCl_3$): δ 142.9, 128.5, 128.5, 125.9, 65.9, 40.7, 38.0, 33.3, 33.3, 25.5, 23.1, 23.1; **HRMS** (ESI): *m/z* calc'd for $C_{14}H_{22}ONa$ $[M + Na]^+$: 229.1563, found: 229.1559.

2-Phenethylpentan-1-ol (S2): Prepared by the general procedure A from 1-phenylhexan-3-one (582 mg, 3.3

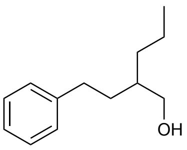

mmol), and isolated as a colorless oil (574 mg, 90% yield for three steps). **IR** (thin film): *ν* 3336, 2955, 2927, 2870, 1603, 1496, 1455, 1043, 1028 cm^{-1}; **^{1}H NMR** (400 MHz, $CDCl_3$): δ 7.33–7.24 (m, 2H), 7.23–7.14 (m, 3H), 3.67–3.52 (m, 2H), 2.65 (t, *J* = 8.0 Hz, 2H), 1.77–1.48 (m, 3H), 1.44–1.28 (m, 4H), 1.24 (brs, 1H), 0.97–0.86 (m, 3H); **^{13}C NMR** (100 MHz, $CDCl_3$): δ 142.9, 128.5, 128.5, 125.9, 65.7, 40.1, 33.4, 33.3, 33.0, 20.1, 14.6; **HRMS** (ESI): *m/z* calc'd for $C_{13}H_{20}ONa$ $[M + Na]^+$: 215.1406, found: 215.1403.

2-Ethyl-4-phenylbutan-1-ol (S3): Prepared by the general procedure A from 1-phenylpentan-3-one (350 mg, 2.1 mmol), and isolated as a colorless oil (259 mg, 67% yield for three steps). **IR** (thin film): *ν* 3338, 2960, 2929, 2874, 1496, 1455, 1043 cm^{-1}; **^{1}H NMR** (400 MHz, $CDCl_3$): δ 7.33–7.24 (m, 2H), 7.22–7.15 (m, 3H), 3.68–3.53 (m, 2H), 2.65 (t, *J* = 8.1 Hz, 2H), 1.78–1.58 (m, 2H), 1.53–1.37 (m, 3H), 0.93 (t, *J* = 7.4 Hz, 3H); **^{13}C NMR** (100 MHz, $CDCl_3$): δ 142.9, 128.5, 128.5 125.9, 65.2, 41.8, 33.4, 32.5, 23.4, 11.2; **HRMS** (ESI): *m*/*z* calc'd for $C_{12}H_{18}ONa$ [M + Na]$^+$: 201.1250, found: 201.1247.

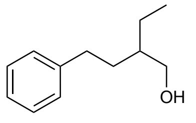

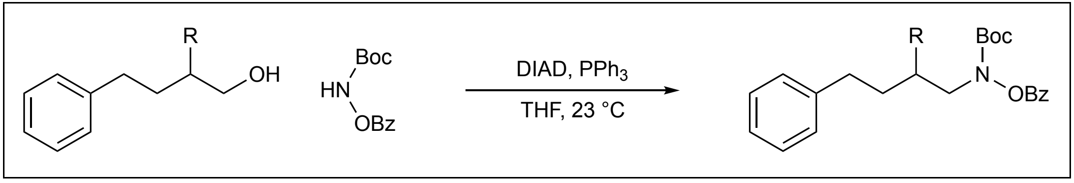

General procedure B: To a solution of alcohol (1.1 equiv), BocNHOBz, and Ph_3P (1.2 equiv) in THF (0.3 M) was added DIAD (1.1 equiv) slowly at 0 °C (ice bath). The solution was warmed to 23 °C and stirred until the hydroxylamine was fully consumed (typically 2–3 hours). The volatile was removed under reduced pressure to give a crude material, which was purified by silica gel column chromatography, eluting with hexane/EtOAc to afford protected hydroxylamine.

***tert*-Butyl (benzoyloxy)(4-methyl-2-phenethylpentyl)carbamate (Boc-11aA)**: Prepared by the general procedure B from **S1** (100 mg, 0.48 mmol), and isolated as a colorless oil (162 mg, 79% yield). **IR** (thin film): *ν* 2955, 2930, 2868, 1765, 1714, 1453, 1368, 1257, 1235, 1162, 1078, 1040, 1013 cm^{-1}; **^{1}H NMR** (400 MHz, $CDCl_3$): δ 8.08–8.00 (m, 2H), 7.66–7.59 (m, 1H), 7.51–7.43 (m, 2H), 7.25–7.18 (m, 2H), 7.18–7.10 (m, 3H), 3.68 (d, *J* = 6.3 Hz, 2H), 2.63 (dd, *J* = 7.2, 8.9 Hz, 2H), 1.91–1.54 (m, 4H), 1.46 (s, 9H), 1.34–1.20 (m, 2H), 0.87 (d, *J* = 6.6 Hz, 3H), 0.83 (d, *J* = 6.5 Hz, 3H); **^{13}C NMR** (100 MHz, $CDCl_3$): δ 164.8, 155.1, 142.7, 133.9, 130.0, 128.8, 128.5, 128.4, 127.8, 125.8, 82.4, 54.4, 41.4, 34.0, 33.9, 32.7, 28.3, 25.4, 23.1, 22.8.; **HRMS** (ESI): *m*/*z* calc'd for $C_{26}H_{35}O_4NNa$ [M + Na]$^+$: 448.2458, found: 448.2457.

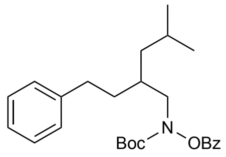

***tert*-Butyl (benzoyloxy)(2-phenethylpentyl)carbamate (Boc-11bA)**: Prepared by the general procedure B from **S2** (19.8 mg, 0.10 mmol), and isolated as a colorless oil (29 mg, 70% yield). **IR** (thin film): *ν* 2957, 2931, 2870, 1765, 1714, 1453, 1393, 1368, 1257, 1162, 1078, 1040, 1017 cm^{-1}; **^{1}H NMR** (400 MHz, $CDCl_3$): δ 8.08–8.01 (m, 2H), 7.66–7.59 (m, 1H), 7.51–7.43 (m, 2H), 7.25–7.19 (m, 2H), 7.18–7.10 (m, 3H), 3.69 (d, *J* = 6.3 Hz, 2H), 2.63 (dd, *J* = 8.0, 8.0 Hz, 2H), 1.85–1.60 (m, 3H), 1.46 (s, 9H), 1.43–1.28 (m, 4H), 0.89 (t, *J* = 7.0 Hz, 3H); **^{13}C NMR** (100 MHz, $CDCl_3$): δ 164.8, 155.0, 142.7, 133.9, 130.0, 128.8, 128.5, 128.4, 127.8, 125.8, 82.4, 54.2, 36.0, 33.8, 33.4, 32.9, 28.3, 19.6, 14.5; **HRMS** (ESI): *m*/*z* calc'd for $C_{25}H_{33}O_4NNa$ [M + Na]$^+$: 434.2302, found: 434.2293.

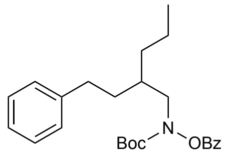

***tert*-Butyl (benzoyloxy)(2-ethyl-4-phenylbutyl)carbamate (Boc-11cA)**: Prepared by the general procedure B

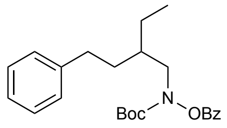

from **S3** (249 mg, 1.4 mmol), and isolated as a colorless oil (488 mg, 88% yield). **IR** (thin film): *ν* 2967, 2933, 2875, 1765, 1714, 1453, 1393, 1368, 1258, 1233, 1162, 1016 cm^{-1}; **^{1}H NMR** (400 MHz, $CDCl_3$): δ 8.09–8.01 (m, 2H), 7.66–7.58 (m, 1H), 7.51–7.43 (m, 2H), 7.25–7.19 (m, 2H), 7.18–7.09 (m, 3H), 3.70 (d, *J* = 6.3 Hz, 2H), 2.63 (dd, *J* = 6.5, 8.7 Hz, 2H), 1.77–1.67 (m, 3H), 1.54–1.44 (m, 1H), 0.92 (t, *J* = 7.4 Hz, 3H); **^{13}C NMR** (100 MHz, $CDCl_3$): δ 164.8, 155.1, 142.7, 133.9, 130.0, 128.8, 128.5, 128.4, 127.8, 125.8, 82.4, 53.8, 37.6, 32.9, 32.9, 28.3, 24.0, 10.7; **HRMS** (ESI): *m*/*z* calc'd for $C_{24}H_{31}O_4NNa$ [M + Na]$^+$: 420.2145, found: 420.2137.

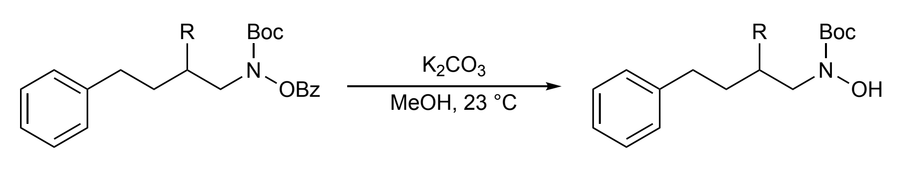

General procedure C: To a solution of Boc-protected hydroxylamine in MeOH (0.5 M) was added K_2CO_3 (1.0 equiv) in one portion at 23 °C). The resulting solution was stirred until the substrate was fully consumed (typically <1 hour). After the addition of H_2O, the mixture was was extracted with EtOAc (x 4). The combined organic layers were washed with brine, dried over Na_2SO_4, and removed under reduced pressure. The crude residue was purified by silica gel column chromatography, eluting with hexane/EtOAc to afford the corresponding *N*-Boc-*O*-H hydroxylamines.

***tert*-Butyl hydroxy(4-methyl-2-phenethylpentyl)carbamate (S4)**: Prepared by the general procedure C from

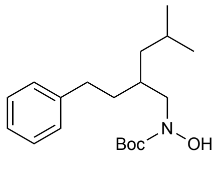

Boc-11aA (80 mg, 0.19 mmol), and isolated as a colorless oil (58 mg, 96% yield). **IR** (thin film): *ν* 3221, 2954, 2930, 2868, 1691, 1455, 1406, 1367, 1166, 1127 cm^{-1}; **^{1}H NMR** (400 MHz, $CDCl_3$): δ 7.31–7.24 (m, 2H), 7.21–7.13 (m, 3H), 3.53–3.37 (m, 2H), 2.69–2.56 (m, 2H), 1.90 (dd, *J* = 5.9, 7.2 Hz, 1H), 1.76–1.53 (m, 4H), 1.48 (s, 9H), 1.29–1.11 (m, 2H), 0.89 (d, *J* = 6.6 Hz, 3H), 0.87 (d, *J* = 6.6 Hz, 3H); **^{13}C NMR** (100 MHz, $CDCl_3$): δ 156.8, 142.9, 128.5, 125.8, 82.0, 53.8, 41.6, 34.0, 33.8, 32.7, 28.5, 25.4, 23.1, 22.9; **HRMS** (ESI): *m*/*z* calc'd for $C_{19}H_{31}O_3NNa$ [M + Na]$^+$: 344.2196, found: 344.2191.

***tert*-Butyl hydroxy(2-phenethylpentyl)carbamate (S5)**: Prepared by the general procedure C from **Boc-11bA**

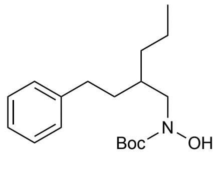

(33 mg, 0.081 mmol), and isolated as a colorless oil (24 mg, 94% yield). **IR** (thin film): *ν* 3217, 2957, 2930, 2870, 1691, 1455, 1367, 1166, 1141 cm^{-1}; **^{1}H NMR** (400 MHz, $CDCl_3$): δ 7.32–7.24 (m, 2H), 7.22–7.13 (m, 3H), 6.63 (s, 1H), 3.46 (d, J = 6.7 Hz, 2H), 2.71 (m, 2H), 1.92–1.83 (m, 1H), 1.72–1.57 (m, 2H), 1.47 (s, 9H), 1.40–1.30 (m, 4H), 0.95–0.86 (m, 3H); **^{13}C NMR** (100 MHz, $CDCl_3$): δ 156.8, 142.9, 128.5, 125.8, 82.0, 53.4, 35.8, 33.9, 33.5, 32.9, 28.5, 19.6, 14.6; **HRMS** (ESI): *m*/*z* calc'd for $C_{18}H_{29}O_3NNa$ [M + Na]$^+$: 330.2040, found: 330.2034.

***tert*-Butyl (2-ethyl-4-phenylbutyl)(hydroxy)carbamate (S6)**: Prepared by the general procedure C from

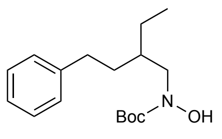

Boc-11cA (250 mg, 0.63 mmol), and isolated as a colorless oil (174 mg, 94% yield). **IR** (thin film): *ν* 3220, 2965, 2932, 2876, 1691, 1455, 1367, 1247, 1167, 1140 cm^{-1}; **^{1}H NMR** (400 MHz, $CDCl_3$): δ 7.32–7.23 (m, 2H), 7.22–7.14 (m, 3H), 6.24 (brs, 1H), 3.46 (dd, *J* = 1.0, 6.7 Hz, 2H),

2.71–2.54 (m, 2H), 1.88–1.74 (m, 1H), 1.70–1.58 (m, 2H), 1.48 (s, 9H), 1.45–1.34 (m, 2H), 0.92 (t, *J* = 7.5 Hz, 3H); **^{13}C NMR** (100 MHz, $CDCl_3$): δ 156.8, 142.8, 128.5, 125.8, 82.0, 53.0, 37.3, 33.0, 28.5, 23.9, 10.6; **HRMS** (ESI): *m/z* calc'd for $C_{17}H_{27}O_3NNa$ [M + Na]$^+$: 316.1883, found: 316.1877.

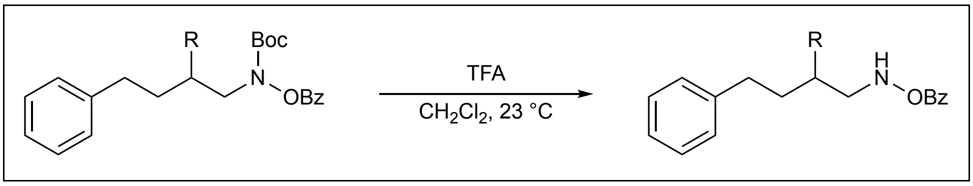

General procedure D: To a solution of Boc-protected hydroxylamine in CH_2Cl_2 (0.5 M) was added TFA (1:1 volume to CH_2Cl_2) at 0 °C (ice bath). The resulting solution was warmed to 23 °C and stirred until the substrate was fully consumed (typically <1 hour). After the dilution with CH_2Cl_2, the mixture was basified with sat aq $NaHCO_3$. The aqueous phase was extracted with CH_2Cl_2. The combined organic layers were washed with brine, dried over Na_2SO_4, and removed under reduced pressure. The crude residue was purified by silica gel column chromatography, eluting with hexane/EtOAc to afford the corresponding *O*-Bz hydroxylamines.

***O*-Benzoyl-*N*-(4-methyl-2-phenethylpentyl)hydroxylamine (11aA)**: Prepared by the general procedure D from

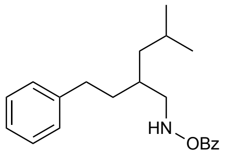

Boc-11aA (70 mg, 0.16 mmol), and isolated as a colorless oil (53 mg, 99% yield). **IR** (thin film): *ν* 3243, 2954, 2927, 2867, 1720, 1452, 1270, 1089, 1066, 1025 cm^{-1}; **^{1}H NMR** (400 MHz, $CDCl_3$): δ 8.06–7.98 (m, 2H), 7.64–7.55 (m, 1H), 7.49–7.42 (m, 2H), 7.30–7.23 (m, 2H), 7.22–7.12 (m, 3H), 3.18–3.05 (m, 2H), 2.74–2.59 (m, 2H), 1.90–1.61 (m, 4H), 1.40–1.21 (m, 2H), 0.90 (d, *J* = 6.6 Hz, 3H), 0.88 (d, *J* = 6.6 Hz, 3H); **^{13}C NMR** (100 MHz, $CDCl_3$): δ 167.1, 142.5, 133.5, 129.5, 128.7, 128.6, 128.5, 128.5, 125.9, 56.3, 42.1, 34.5, 33.5, 33.0, 25.5, 23.0, 22.9; **HRMS** (ESI): *m/z* calc'd for $C_{21}H_{28}O_2N$ [M + H]$^+$: 326.2115, found: 326.2108.

***O*-Benzoyl-*N*-(2-phenethylpentyl)hydroxylamine (11bA)**: Prepared by the general procedure D from

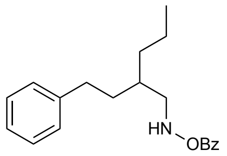

Boc-11bA (19.5 mg, 0.047 mmol), and isolated as a colorless oil (14 mg, 94% yield). **IR** (thin film): *ν* 3242, 2956, 2929, 2870, 1719, 1452, 1270, 1089, 1066, 1025 cm^{-1}; **^{1}H NMR** (400 MHz, $CDCl_3$): δ 8.03–7.97 (m, 2H), 7.63–7.56 (m, 1H), 7.48–7.42 (m, 2H), 7.29–7.22 (m, 2H), 7.21–7.12 (m, 3H), 3.12 (d, *J* = 5.7 Hz, 2H), 2.67 (t, *J* = 7.7 Hz, 2H), 1.83–1.65 (m, 3H), 1.56–1.32 (m, 4H), 0.92 (t, *J* = 8.0 Hz, 3H); **^{13}C NMR** (100 MHz, $CDCl_3$): δ 167.1, 142.5, 133.5, 129.5, 128.7, 128.5, 128.5, 128.5, 125.9, 56.0, 35.6, 34.5, 34.1, 33.2, 19.9, 14.5.; **HRMS** (ESI): *m/z* calc'd for $C_{20}H_{26}O_2N$ [M + H]$^+$: 312.1958, found: 312.1956.

***O*-Benzoyl-*N*-(2-ethyl-4-phenylbutyl)hydroxylamine (11cA)**: Prepared by the general procedure D from

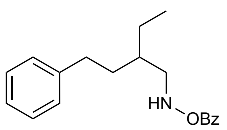

Boc-11cA (200 mg, 0.50 mmol), and isolated as a colorless oil (137 mg, 92% yield). **IR** (thin film): *ν* 3242, 2961, 2930, 2874, 1719, 1452, 1271, 1089, 1066, 1025 cm^{-1}; **^{1}H NMR** (400 MHz, $CDCl_3$): δ 8.05–7.93 (m, 3H), 7.63–7.55 (m, 1H), 7.50–7.42 (m, 2H), 7.31–7.22 (m, 2H), 7.22–7.13 (m, 3H), 3.11 (d, *J* = 5.3 Hz, 2H), 2.67 (t, *J* = 7.8 Hz, 2H), 1.84–1.63 (m, 3H), 1.60–1.46 (m, 3H), 0.95 (t, *J* = 7.5 Hz, 3H); **^{13}C NMR** (100 MHz, $CDCl_3$): δ 167.1, 142.6, 133.5, 129.5, 128.7, 128.6, 128.5, 128.5, 125.9, 55.6, 37.1, 33.6, 33.2, 24.6, 10.9; **HRMS** (ESI): *m/z* calc'd for $C_{19}H_{24}O_2N$ [M + H]$^+$: 298.1802, found: 298.1795.

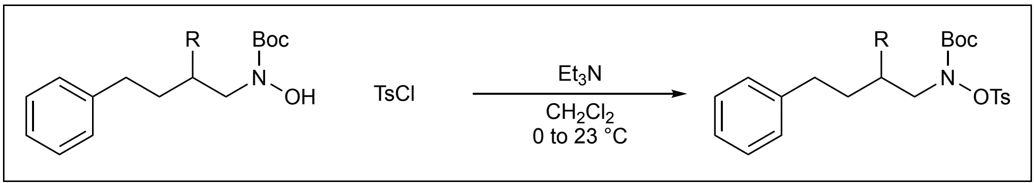

General procedure E: To a solution of Boc-protected hydroxylamine in CH_2Cl_2 (0.1 M) was added Et_3N (1.1 equiv) and pTsCl (1.1 equiv) at 0 °C (ice bath). The resulting solution was warmed to 23 °C and stirred until the substrate was fully consumed. After the addition of sat aq NH_4Cl, the aqueous phase was extracted with CH_2Cl_2 (x 2). The combined organic layers were washed with brine, dried over Na_2SO_4, and removed under reduced pressure. The crude residue was purified by silica gel column chromatography, eluting with hexane/EtOAc to afford the corresponding products.

***tert*-Butyl (4-methyl-2-phenethylpentyl)(tosyloxy)carbamate (11aB)**: Prepared by the general procedure E

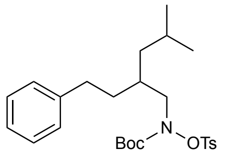

from **S4** (30 mg, 0.094 mmol), and isolated as a colorless oil (41 mg, 93% yield). **IR** (thin film): *ν* 2955, 2930, 2868, 1747, 1717, 1381, 1369, 1191, 1179, 1158 cm^{-1}; **^{1}H NMR** (400 MHz, $CDCl_3$): δ 7.90–7.83 (m, 2H), 7.37–7.32 (m, 2H), 7.29–7.22 (m, 2H), 7.20–7.12 (m, 3H), 3.76–3.53 (m, 2H), 2.69–2.48 (m, 2H), 2.45 (s, 3H), 2.09–1.97 (m, 1H), 1.69–1.48 (m, 4H), 1.20 (s, 9H), 1.07 (dt, *J* = 6.9, 13.7 Hz, 1H), 0.87 (d, *J* = 6.9 Hz, 3H), 0.84 (d, *J* =6.9 Hz, 3H); **^{13}C NMR** (100 MHz, $CDCl_3$): δ 155.4, 145.8, 142.7, 131.6, 129.8, 129.7, 128.5, 128.4, 125.8, 83.1, 56.2, 41.2, 33.8, 33.2, 32.4, 27.8, 25.4, 23.2, 22.7, 21.8; **HRMS** (ESI): *m/z* calc'd for $C_{26}H_{37}O_5NNaS$ $[M + Na]^+$: 498.2285, found: 498.2278.

***tert*-Butyl (2-phenethylpentyl)(tosyloxy)carbamate (11bB)**: Prepared by the general procedure E from **S5** (16

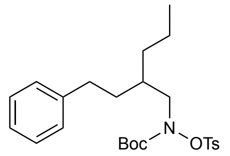

mg, 0.053 mmol), and isolated as a colorless oil (21 mg, 88% yield). **IR** (thin film): *ν* 2957, 2931, 2871, 1747, 1718, 1382, 1370, 1192, 1179, 1159 cm^{-1}; **^{1}H NMR** (400 MHz, $CDCl_3$): δ 7.90–7.81 (m, 2H), 7.38–7.31 (m, 2H), 7.30–7.22 (m, 2H), 7.20–7.12 (m, 3H), 3.64 (brs, 2H), 2.68–2.49 (m, 2H), 2.45 (s, 3H), 2.03–1.92 (m, 1H), 1.64–1.50 (m, 2H), 1.39–1.23 (m, 4H), 1.20 (s, 9H), 0.93–0.82 (m, 3H); **^{13}C NMR** (100 MHz, $CDCl_3$): δ 155.4, 145.9, 142.7, 131.6, 129.8, 129.7, 128.5, 128.4, 125.8, 83.1, 55.8, 35.1, 33.5, 33.3, 32.6, 27.8, 21.8, 19.4, 14.5; **HRMS** (ESI): *m/z* calc'd for $C_{25}H_{35}O_5NNaS$ $[M + Na]^+$: 484.2128, found: 484.2122.

***tert*-Butyl (2-ethyl-4-phenylbutyl)(tosyloxy)carbamate (11cB)**: Prepared by the general procedure E from **S6**

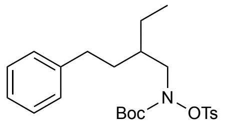

(100 mg, 0.34 mmol), and isolated as a white solid (150 mg, 98% yield). **m.p.** 51–52 °C; **IR** (thin film): *ν* 2965, 2932, 1746, 1718, 1380, 1370, 1192, 1179, 1159 cm^{-1}; **^{1}H NMR** (400 MHz, $CDCl_3$): δ 7.90–7.81 (m, 2H), 7.38–7.31 (m, 2H), 7.30–7.22 (m, 2H), 7.20–7.11 (m, 3H), 3.64 (brs, 2H), 2.66–2.50 (m, 2H), 2.45 (s, 3H), 2.00–1.88 (m, 1H), 1.65–1.49 (m, 2H), 1.45–1.29 (m, 2H), 1.21 (s, 9H), 0.88 (t, *J* = 7.4 Hz, 3H); **^{13}C NMR** (100 MHz, $CDCl_3$): δ 155.4, 145.9, 142.7, 131.6, 129.8, 129.7, 128.5, 128.5, 125.9, 83.1, 55.5, 36.5, 32.8, 32.7, 27.8, 23.5, 21.8, 10.3; **HRMS** (ESI): *m/z* calc'd for $C_{24}H_{33}O_5NNaS$ $[M + Na]^+$: 470.1972, found: 470.1964.

***tert*-Butyl (4-(4-fluorophenyl)butyl-4-*d*)(hydroxy)carbamate (S7)**: Prepared by the general procedure C from

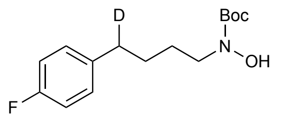

tert-butyl (benzoyloxy)(4-(4-fluorophenyl)butyl-4-*d*)carbamate[2] (2.13 g, 5.3 mmol), and isolated as a colorless oil (1.62 g, 99% yield). **IR** (thin film): *ν* 3230, 2978, 2933, 2866, 1692, 1509, 1368, 1220, 1159, 1121 cm^{-1}; **^{1}H NMR** (400 MHz, $CDCl_3$): δ 7.15–7.08 (m, 2H), 6.99–6.90 (m, 2H), 3.54–3.44 (m, 2H), 2.64–2.54 (m, 1H), 1.69–1.59 (m, 4H), 1.45 (s, 9H); **^{13}C NMR** (100 MHz, $CDCl_3$): δ 161.4 (d, *J* = 243.2 Hz), 157.1, 137.9 (d, *J* = 3.3 Hz), 129.8 (d, *J* = 7.7 Hz), 115.2 (d, *J* = 20.9 Hz), 82.0, 49.9, 34.4 (t, *J* = 18.9 Hz), 28.5, 27.8, 26.5; **^{19}F NMR** (376 MHz, $CDCl_3$) δ –117.94; **HRMS** (ESI): *m*/*z* calc'd for $C_{15}H_{21}DO_3NFNa$ [M + Na]$^+$: 307.1539, found: 307.1532.

***tert*-Butyl (4-(4-fluorophenyl)butyl-4-*d*)((4-methoxybenzoyl)oxy)carbamate (Boc-17)**: Prepared by the general

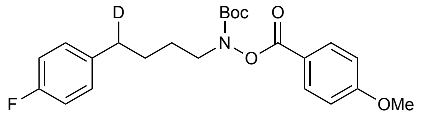

procedure E from **S7** (180 mg, 0.63 mmol) and p-MeO-benzoyl chloride (118 mg, 0.69 mmol) instead of pTsCl, and isolated as a colorless oil (229 mg, 86% yield). **IR** (thin film): *ν* 2977, 2935, 1758, 1714, 1606, 1509, 1368, 1254, 1221, 1159 cm^{-1}; **^{1}H NMR** (400 MHz, $CDCl_3$): δ 8.04−7.95 (m, 2H), 7.15–7.06 (m, 2H), 6.98–6.88 (m, 4H), 3.88 (s, 3H), 3.70 (t, *J* = 6.5 Hz, 2H), 2.59 (t, *J* = 7.1 Hz, 1H), 1.75–1.60 (m, 4H), 1.45 (s, 9H); **^{13}C NMR** (100 MHz, $CDCl_3$): δ 164.5, 164.2, 161.4 (d, J = 242.8 Hz), 155.1, 137.8 (d, J = 3.3 Hz), 132.2, 129.8 (d, J = 7.7 Hz), 120.0, 115.1 (d, J = 21.0 Hz), 114.1, 82.3, 55.7, 50.4, 34.3 (t, J = 19.1 Hz), 28.5, 28.3, 26.8; **^{19}F NMR** (376 MHz, $CDCl_3$) δ –117.94; **HRMS** (ESI): *m*/*z* calc'd for $C_{23}H_{27}DO_5NFNa$ [M + Na]$^+$: 441.1906, found: 441.1900.

***N*-(4-(4-Fluorophenyl)butyl-4-*d*)-*O*-(4-methoxybenzoyl)hydroxylamine (17)**: Prepared by the general

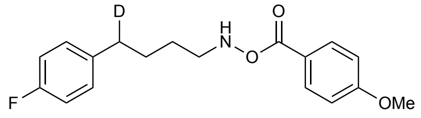

procedure D from **Boc-17** (120 mg, 0.28 mmol), and isolated as a colorless oil (89 mg, 97% yield). **IR** (thin film): *ν* 3235, 2935, 2862, 1714, 1606, 1509, 1258, 1219, 1169 cm^{-1}; **^{1}H NMR** (400 MHz, $CDCl_3$): δ 8.00−7.92 (m, 2H), 7.16–7.08 (m, 2H), 7.00–6.89 (m, 4H), 3.87 (s, 3H), 3.13 (dt, *J* = 6.2, 7.5 Hz, 2H), 2.61 (t, *J* = 7.4 Hz, 1H), 1.77–1.58 (m, 4H); **^{13}C NMR** (100 MHz, $CDCl_3$): δ 166.9, 163.8, 161.4 (d, *J* = 243.2 Hz), 137.8 (d, *J* = 3.3 Hz), 131.6, 129.8 (d, *J* = 7.7 Hz), 120.8, 115.2 (d, *J* = 20.9 Hz), 114.0, 55.6, 52.5, 34.6 (t, *J* = 19.3 Hz), 28.9, 26.8.; **^{19}F NMR** (376 MHz, $CDCl_3$) δ –117.84; **HRMS** (ESI): *m*/*z* calc'd for $C_{18}H_{20}DO_3NF$ [M + H]$^+$: 319.1563, found: 319.1558.

***tert*-Butyl ((4-cyanobenzoyl)oxy)(4-(4-fluorophenyl)butyl-4-*d*)carbamate (Boc-18)**: Prepared by the general

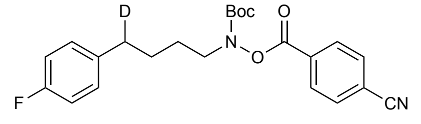

procedure E from **S7** (180 mg, 0.63 mmol) and p-CN-benzoyl chloride (115 mg, 0.69 mmol) instead of pTsCl, and isolated as a colorless oil (260 mg, 99% yield). **IR** (thin film): *ν* 2979, 2934, 2232, 1770, 1720, 1509, 1369, 1253, 1221, 1159 cm^{-1}; **^{1}H NMR** (400 MHz, $CDCl_3$): δ 8.16–8.09 (m, 2H), 7.82–7.74 (m, 2H), 7.15–7.06 (m, 2H), 6.98–6.88 (m, 2H), 3.71 (t, *J* = 6.6 Hz, 2H), 2.60 (t, *J* = 7.2 Hz, 1H), 1.75–1.60 (m, 4H), 1.45 (s, 9H); **^{13}C NMR** (100 MHz, $CDCl_3$): δ 163.3, 161.4 (d, *J* = 243.6 Hz), 154.9, 137.6 (d, *J* = 3.3 Hz), 132.6, 131.7, 130.5, 129.8 (d, *J* = 8.1 Hz), 117.8, 117.4, 115.2 (d, *J* = 21.3), 83.0, 50.8, 34.3 (t, *J* = 19.9 Hz), 28.5, 28.4, 26.7; **^{19}F NMR** (376 MHz, $CDCl_3$) δ –117.67; **HRMS** (ESI): *m*/*z* calc'd for $C_{23}H_{24}DO_4N_2FNa$ [M + Na]$^+$: 436.1753, found: 436.1748.

4-((((4-(4-Fluorophenyl)butyl-4-*d*)amino)oxy)carbonyl)benzonitrile (18): Prepared by the general procedure D

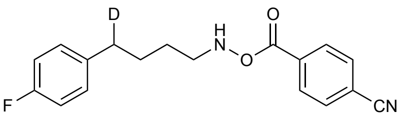

from **Boc-18** (120 mg, 0.29 mmol), and isolated as a white solid (79 mg, 87% yield). **m.p.** 42–43 °C **IR** (thin film): *ν* 3242, 2934, 2863, 2231, 1725, 1509, 1270, 1219, 1093 cm^{-1}; **^{1}H NMR** (400 MHz, $CDCl_3$): δ 8.12–8.06 (m, 2H), 7.79–7.72 (m, 2H), 7.17–7.07 (m, 2H), 6.99–6.91 (m, 2H), 3.19 (brt, *J* = 6.8 Hz, 2H), 2.62 (t, *J* = 7.4 Hz, 1H), 1.78–1.57 (m, 4H); **^{13}C NMR** (100 MHz, $CDCl_3$): δ 165.3, 161.4 (d, *J* = 243.2 Hz), 137.6 (d, *J* = 3.3 Hz), 132.5, 132.4, 130.0, 129.8 (d, *J* = 7.7 Hz), 117.9, 117.0, 115.2 (d, *J* = 21.0 Hz), 52.5, 34.5 (t, *J* = 719.5 Hz), 28.8, 26.6; **^{19}F NMR** (376 MHz, $CDCl_3$) δ –117.61; **HRMS** (ESI): *m*/*z* calc'd for $C_{18}H_{17}DO_2N_2F$ $[M + H]^+$: 314.1410, found: 314.1402.

3. Rh-catalyzed C–H functionalization

Procedure for Table 1: To a 10 mL test tube equipped with a magnetic stirring bar were added *O*-benzoylhydroxylamine **11aA** (0.1 mmol), HFIP (1 mL, 0.1 M), and additive, followed by the addition of $Rh_2(esp)_2$ (1 mol%, 0.002 mmol) at 23 °C. The resulting mixture was stirred at the same temperature for 16 h. The reaction mixture was concentrated under reduced pressure, which was analyzed by ^{1}H NMR to determine the conversion and the regioselectivity.

Procedure for Table 2: To a 10 mL test tube equipped with a magnetic stirring bar were added hydroxylamine (0.2 mmol), solvent (2 mL, 0.1 M), and TFA, followed by the addition of $Rh_2(esp)_2$ (1 or 2 mol%) at 23 °C. The resulting mixture was stirred at the same temperature. After full consumption of the substrates by TLC analysis, the reaction mixture was concentrated under reduced pressure. The residue was dissolved in EtOAc and sat aq $NaHCO_3$. The aquous phase was extracted with EtOAc (x3). The combined organic layers were washed with brine, dried over Na_2SO_4, and removed. The crude mixture was analyzed by ^{1}H NMR to determine the selectivity. To the crude residue were added CH_2Cl_2 (1 mol, 0.2 M), Et_3N (2.0 equiv), and TsCl (2.0 equiv). The solution was stirred for 2 h at 23 °C and directly placed on a silica gel column chromatography, with hexane/EtOAc as the eluent, affording the *N*-Ts product as a diastereomixture.

2,2-Dimethyl-4-phenethyl-1-tosylpyrrolidine (Ts-13a): white solid; **m.p.** 75–77 °C; **IR** (thin film): *ν* 2966, 2924,

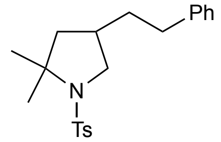

1600, 1495, 1455, 1335, 1155, 1103, 1092 cm^{-1}; **^{1}H NMR** (400 MHz, $CDCl_3$): δ 7.76–7.69 (m, 2H), 7.33–7.23 (m, 4H), 7.22–7.16 (m, 1H), 7.15–7.09 (m, 2H), 3.68–3.61 (m, 1H), 2.83 (dd, *J* = 9.6, 9.6 Hz, 1H), 2.64–2.50 (m, 2H), 2.42 (s, 3H), 2.28–2.14 (m, 1H), 1.93 (ddd, *J* = 1.1, 6.3, 12.1 Hz, 1H), 1.62 (dt, *J* = 7.2, 8.5 Hz, 2H), 1.47 (s, 3H), 1.42 (s, 3H); **^{13}C NMR** (100 MHz, $CDCl_3$): δ 142.7, 141.8, 138.7, 129.5, 128.6, 128.4, 127.3, 126.1, 65.7, 54.7, 49.6, 35.4, 35.0, 34.6, 29.1, 28.7, 21.6; **HRMS** (ESI): *m*/*z* calc'd for $C_{21}H_{28}O_2NS$ $[M + H]^+$: 358.1835, found: 358.1829.

4-Isobutyl-2-phenyl-1-tosylpyrrolidine (Ts-12a): colorless oil; **IR** (thin film): *ν* 2956, 2925, 2869, 1598, 1453, 1348,

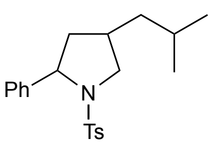

1160, 1093 cm^{-1}; **^{1}H NMR** (400 MHz, $CDCl_3$): δ 7.69–7.65 (m, 0.5H), 7.62–7.57 (m, 2H), 7.32–7.19 (m, 8.75H), 4.84–4.80 (m, 0.25H), 4.66–4.60 (m, 1H), 3.91–3.85 (m, 1H), 3.78–3.72 (m, 0.25H), 3.11–3.04 (m, 1H), 2.91–2.85 (m, 0.25H), 2.44–2.33 (m, 5.0H), 1.92–1.73 (m, 1.25H),

1.63–1.54 (m, 0.25H), 1.51–1.40 (m, 2.25H), 1.20–1.14 (m, 2H), 1.09–1.03 (m, 0.5H), 0.84–0.76 (m, 7.5H); ^{13}**C NMR** (100 MHz, $CDCl_3$): δ 143.5, 143.4, 143.3, 143.1, 135.9, 135.1, 129.7, 129.6, 128.5, 128.4, 127.7, 127.6, 127.3, 127.1, 126.5, 126.2, 64.5, 63.0, 55.7, 54.7, 44.4, 44.4, 42.1, 41.6, 36.9, 34.8, 26.9, 26.9, 22.9, 22.7, 22.6, 21.7, 21.6; **HRMS** (ESI): *m*/*z* calc'd for $C_{21}H_{28}O_2NS$ [M + H]$^+$: 358.1835, found: 358.1828.

The relative configuration of **Ts-12a** was determined by NOE analysis. The major diastereomer of **Ts-12b** and **Ts-12c** was assigned by analogy.

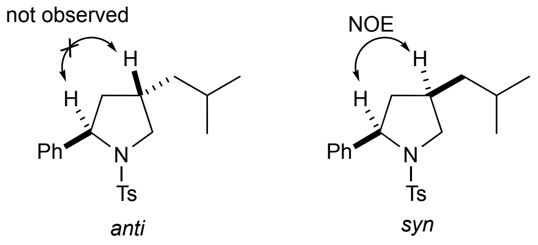

2-Phenyl-4-propyl-1-tosylpyrrolidine (Ts-12b): colorless oil; **IR** (thin film): *ν* 2958, 2925, 2871, 1598, 1494, 1453, 1348, 1160, 1092 cm^{-1}; ^{1}**H NMR** (400 MHz, $CDCl_3$): δ 7.70–7.65 (m, 0.5H), 7.61–7.57 (m, 2H), 7.31–7.18 (m, 8.75H), 4.85–4.81 (m, 0.25H), 4.65–4.59 (m, 1H), 3.91–3.84 (m, 1H), 3.78–3.72 (m, 0.25H), 3.13–3.06 (m, 1H), 2.93–2.87 (m, 0.25H), 2.44–2.35 (m, 4.75H), 2.27–2.20 (m, 0.25H), 1.93–1.86 (m, 0.25H), 1.81–1.69 (m, 1H), 1.63–1.57 (m, 0.25H), 1.52–1.42 (m, 1H), 1.33–1.11 (m, 5.0H), 0.88–0.79 (m, 3.75H); ^{13}**C NMR** (100 MHz, $CDCl_3$): δ 143.5, 143.4, 143.3, 143.1, 135.8, 135.1, 129.7, 129.6, 128.5, 128.4, 127.7, 127.6, 127.2, 127.1, 126.5, 126.2, 64.6, 63.1, 55.5, 54.6, 44.1, 41.9, 38.7, 36.7, 35.1, 34.6, 21.7, 21.7, 21.5, 21.4, 14.3, 14.2; **HRMS** (ESI): *m*/*z* calc'd for $C_{20}H_{26}O_2NS$ [M + H]$^+$: 344.1679, found: 344.1674.

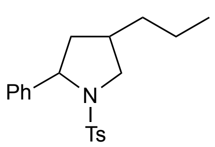

4-Ethyl-2-phenyl-1-tosylpyrrolidine (Ts-12c): white solid; **m.p.** 81–83 °C; **IR** (thin film): *ν* 2961, 2926, 2874, 1598, 1450, 1348, 1159, 1092 cm^{-1}; ^{1}**H NMR** (400 MHz, $CDCl_3$): δ 7.70–7.65 (m, 0.5H), 7.62–7.56 (m, 2H), 7.32–7.19 (m, 8.75H), 4.87–4.83 (m, 0.25H), 4.62 (dd, *J* = 7.1, 9.5 Hz, 1H), 3.87 (ddd, *J* = 1.3, 7.3, 11.1 Hz, 1H), 3.78–3.72 (m, 0.25H), 3.15–3.07 (m, 1H), 2.94–2.88 (m, 0.25H), 2.46–2.34 (m, 3.75H), 1.93–1.87 (m, 0.25H), 1.79–1.17 (m, 6.0H), 0.87–0.78 (m, 3.75H); ^{13}**C NMR** (100 MHz, $CDCl_3$): δ 143.5, 143.4, 143.3, 143.1, 135.8, 135.2, 129.7, 129.6, 128.5, 128.4, 127.7, 127.6, 127.3, 127.1, 126.5, 126.2, 64.6, 63.2, 55.3, 54.4, 43.8, 41.5, 40.6, 38.6, 25.8, 25.5, 21.7, 21.7, 12.6, 12.6; **HRMS** (ESI): *m*/*z* calc'd for $C_{19}H_{24}O_2NS$ [M + H]$^+$: 330.1522, found: 330.1516.

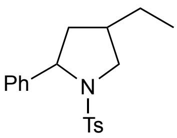

4. References

(1) Fulmer, G. R.; Miller, A. J. M.; Sherden, N. H.; Gottlieb, H. E.; Nudelman, A.; Stoltz, B. M.; Bercaw, J. E.; Goldberg, K. I. (2010) Organometallics, 29, 2176.

(2) Noda, H., Asada, Y., and Shibasaki, M. (2020). *O*-Benzoylhydroxylamines as Alkyl Nitrene Precursors: Synthesis of Saturated N-Heterocycles from Primary Amines. Org. Lett. 22, 8769–8773.

5. NMR Spectra

4-Methyl-2-phenethylpentan-1-ol (S1):

^{1}H NMR (400 MHz, $CDCl_3$)

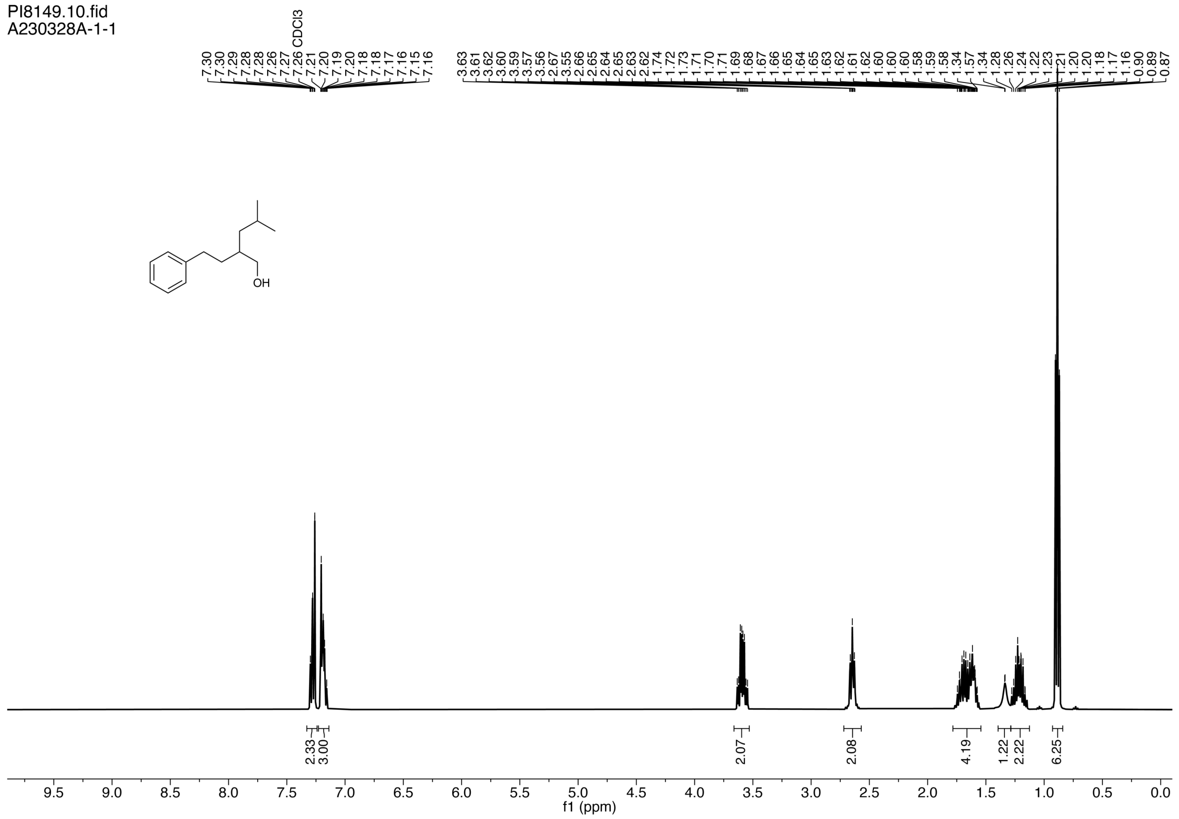

^{13}C NMR (100 MHz, $CDCl_3$)

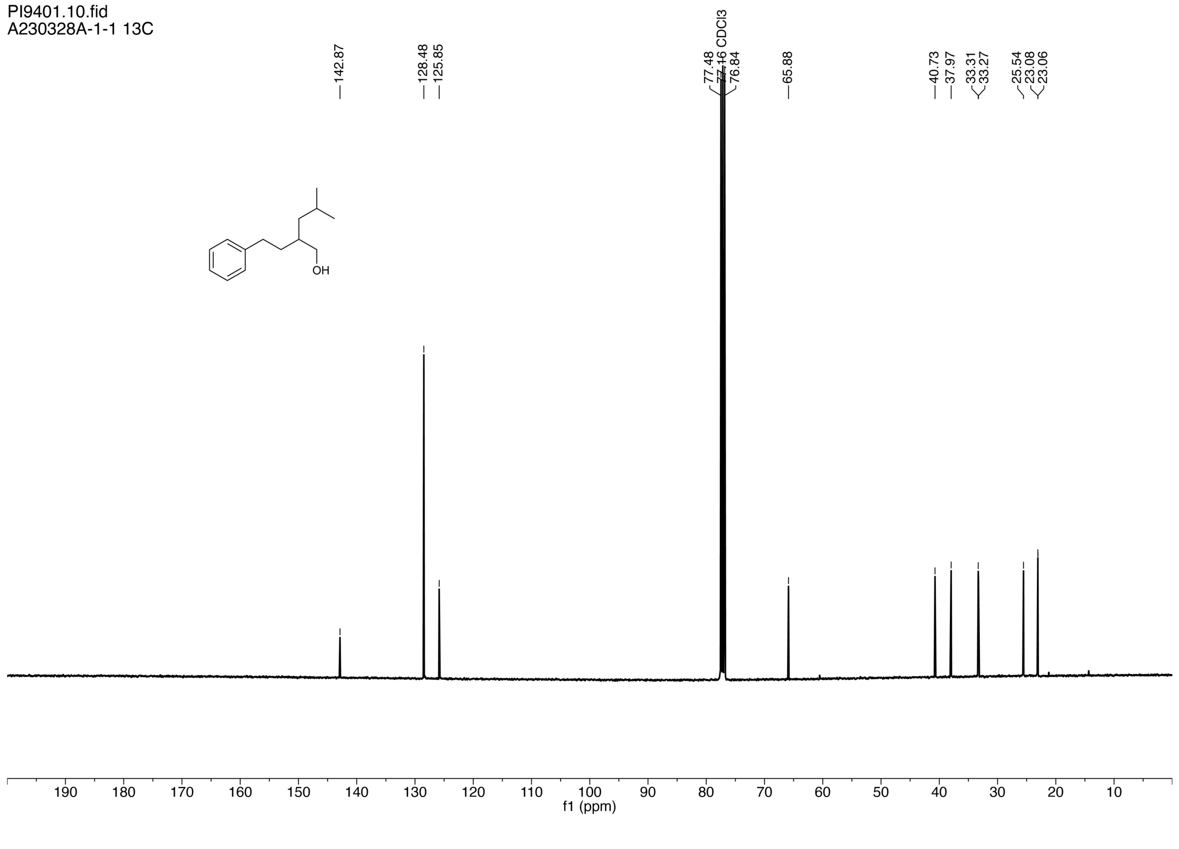

2-Phenethylpentan-1-ol (S2):

^{1}**H NMR** (400 MHz, $CDCl_3$)

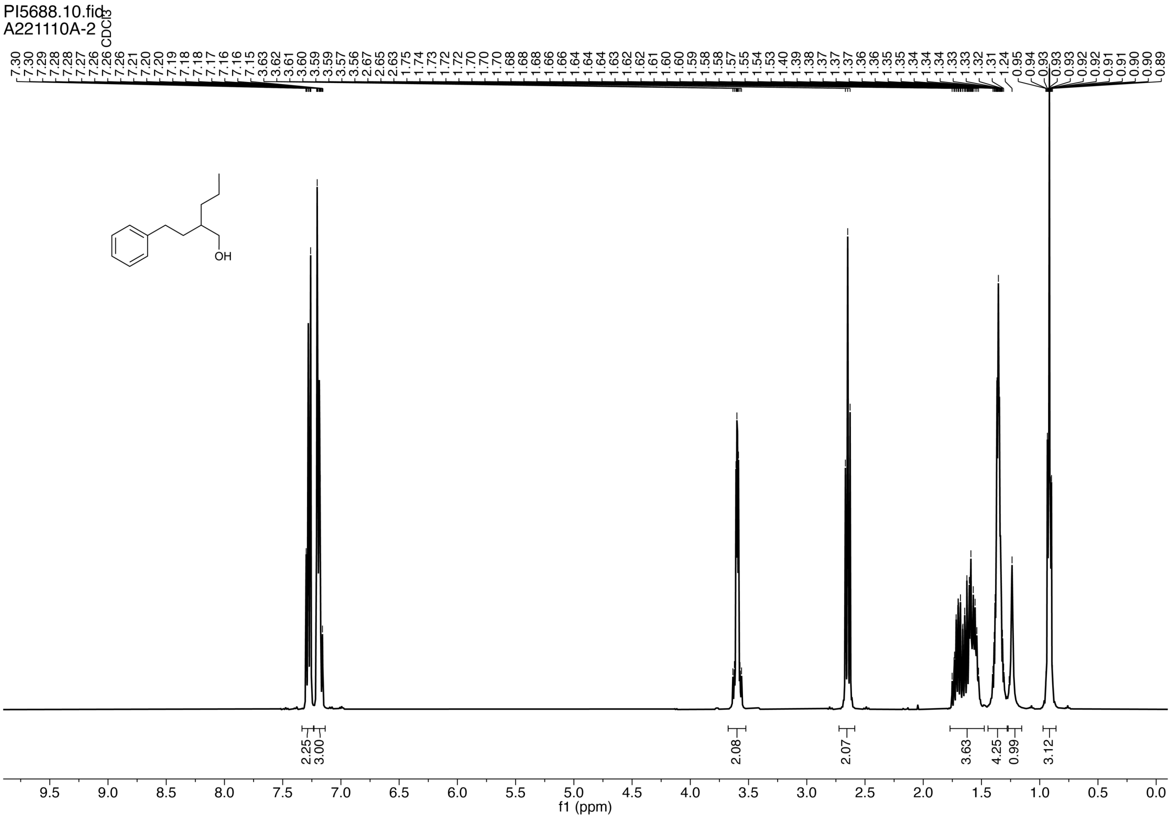

^{13}**C NMR** (100 MHz, $CDCl_3$)

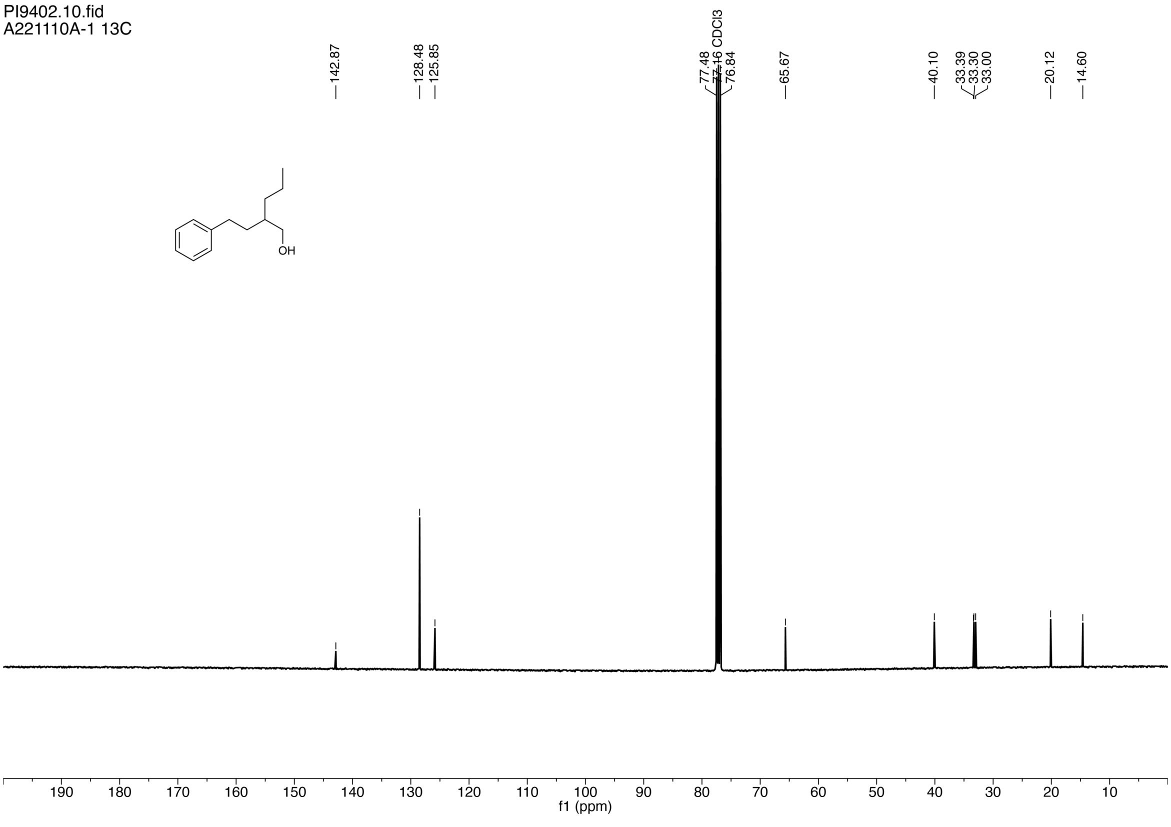

2-Ethyl-4-phenylbutan-1-ol (S3):

^{1}H NMR (400 MHz, $CDCl_3$)

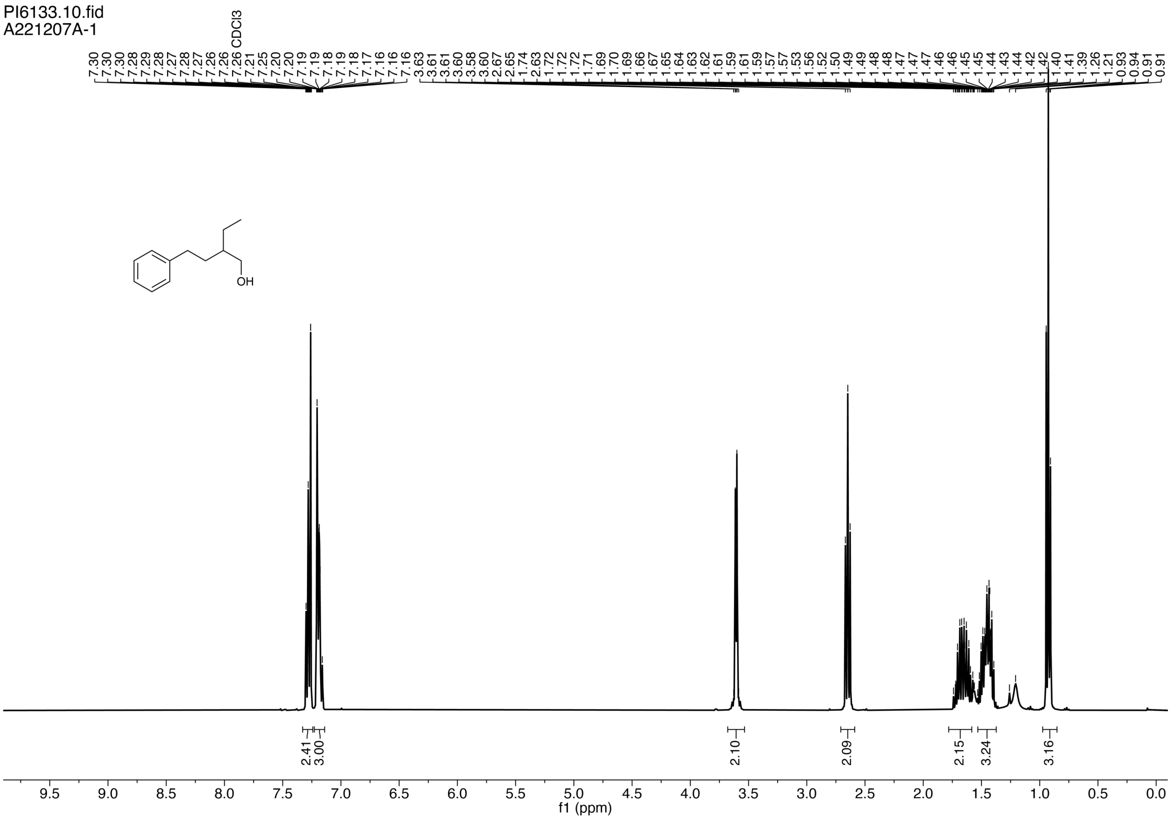

^{13}C NMR (100 MHz, $CDCl_3$)

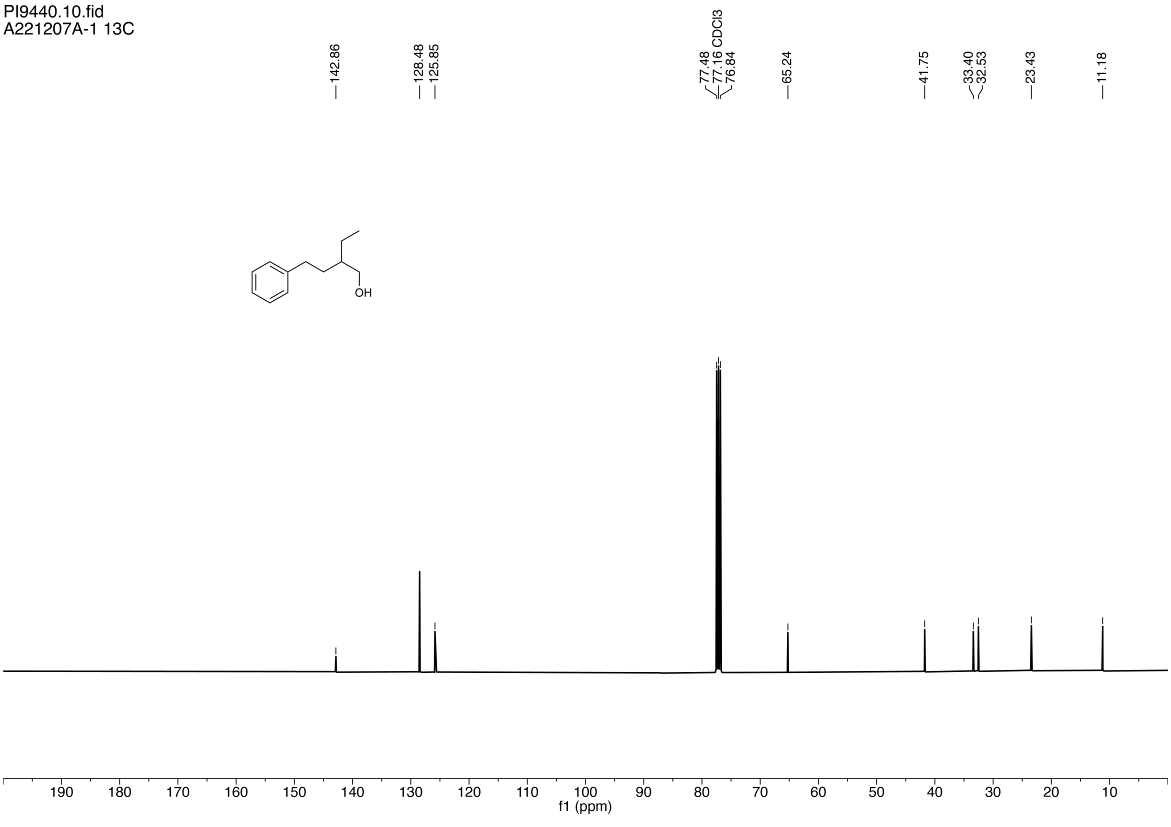

tert-Butyl (benzoyloxy)(4-methyl-2-phenethylpentyl)carbamate (Boc-11aA):

^{1}H NMR (400 MHz, $CDCl_3$)

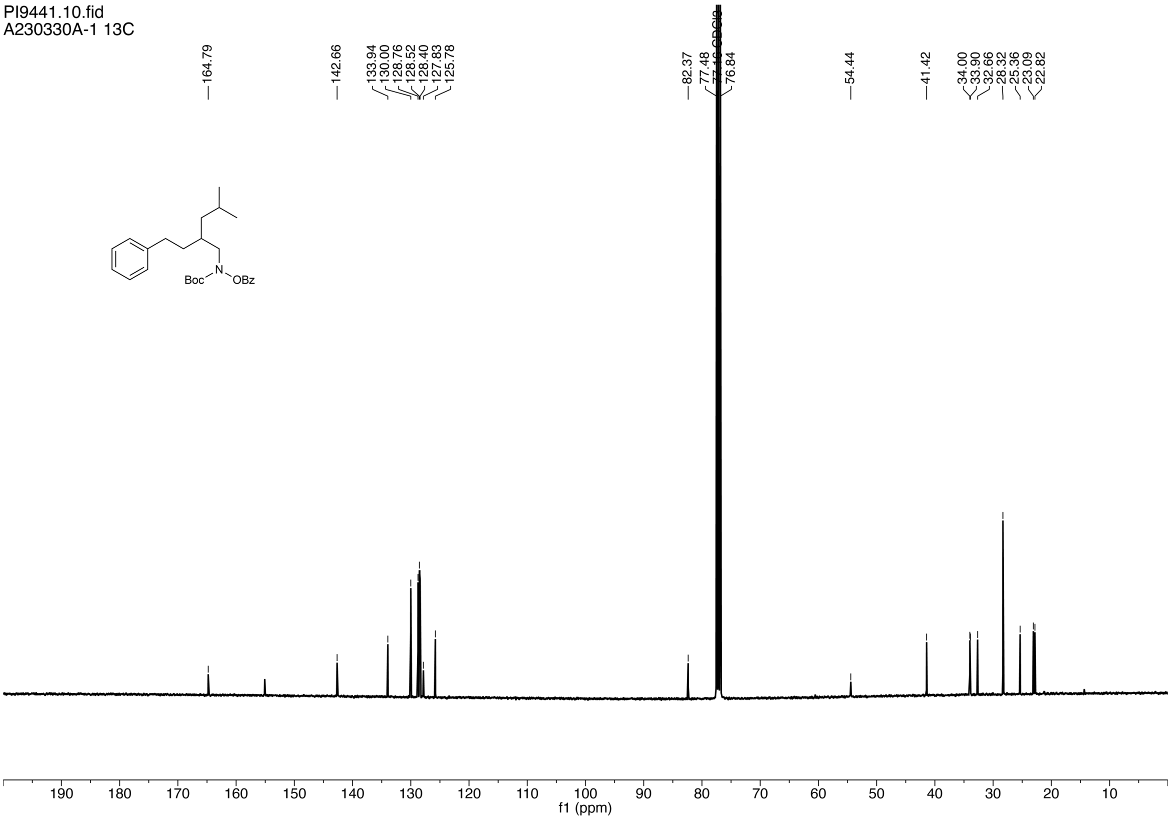

^{13}C NMR (100 MHz, $CDCl_3$)

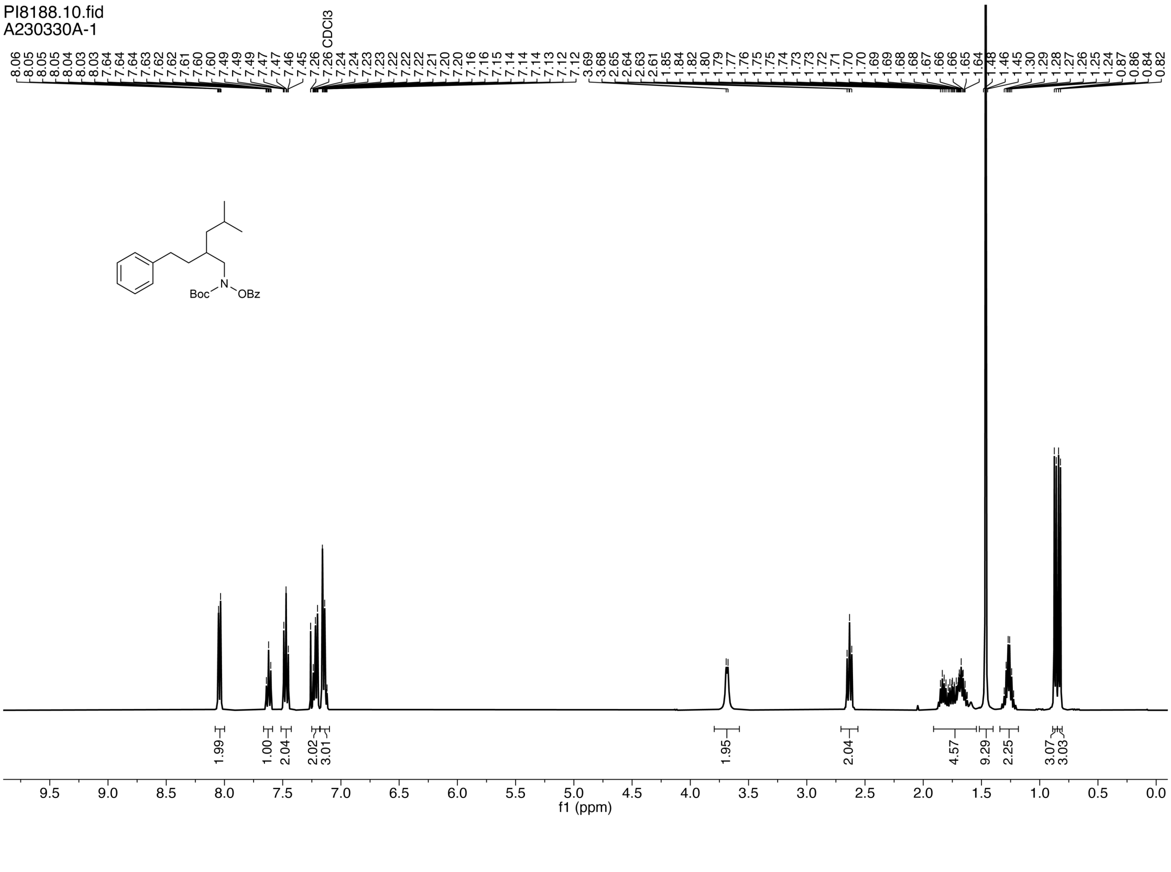

***tert*-Butyl (benzoyloxy)(2-phenethylpentyl)carbamate (Boc-11bA)**:

^{1}H NMR (400 MHz, $CDCl_3$)

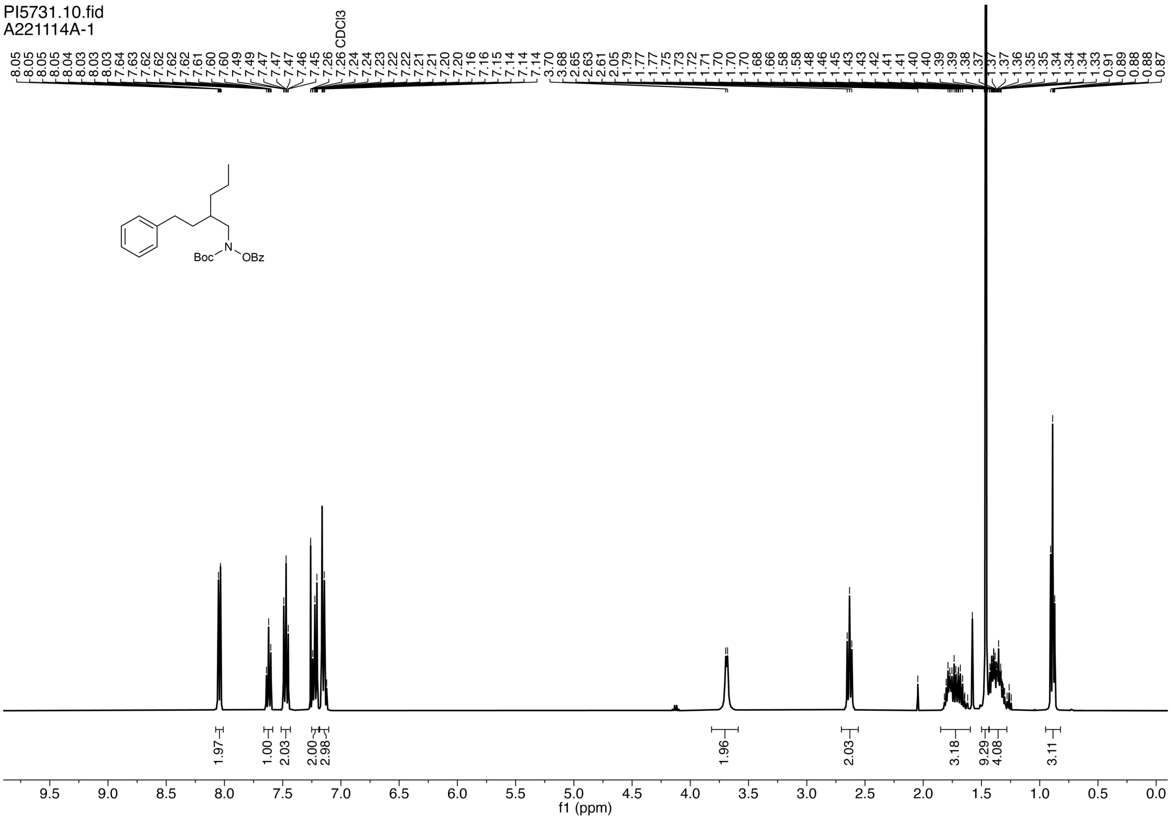

^{13}C NMR (100 MHz, $CDCl_3$)

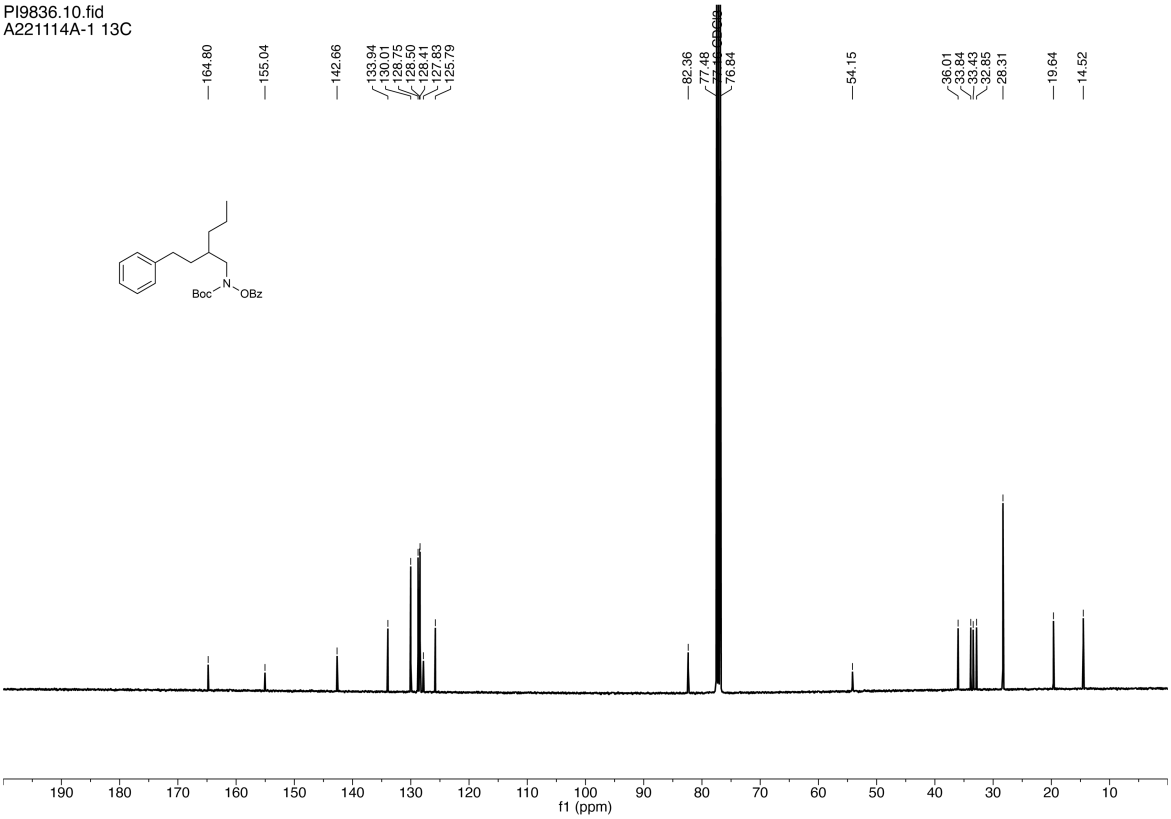

***tert*-Butyl (benzoyloxy)(2-ethyl-4-phenylbutyl)carbamate (Boc-11cA)**:

^{1}H NMR (400 MHz, $CDCl_3$)

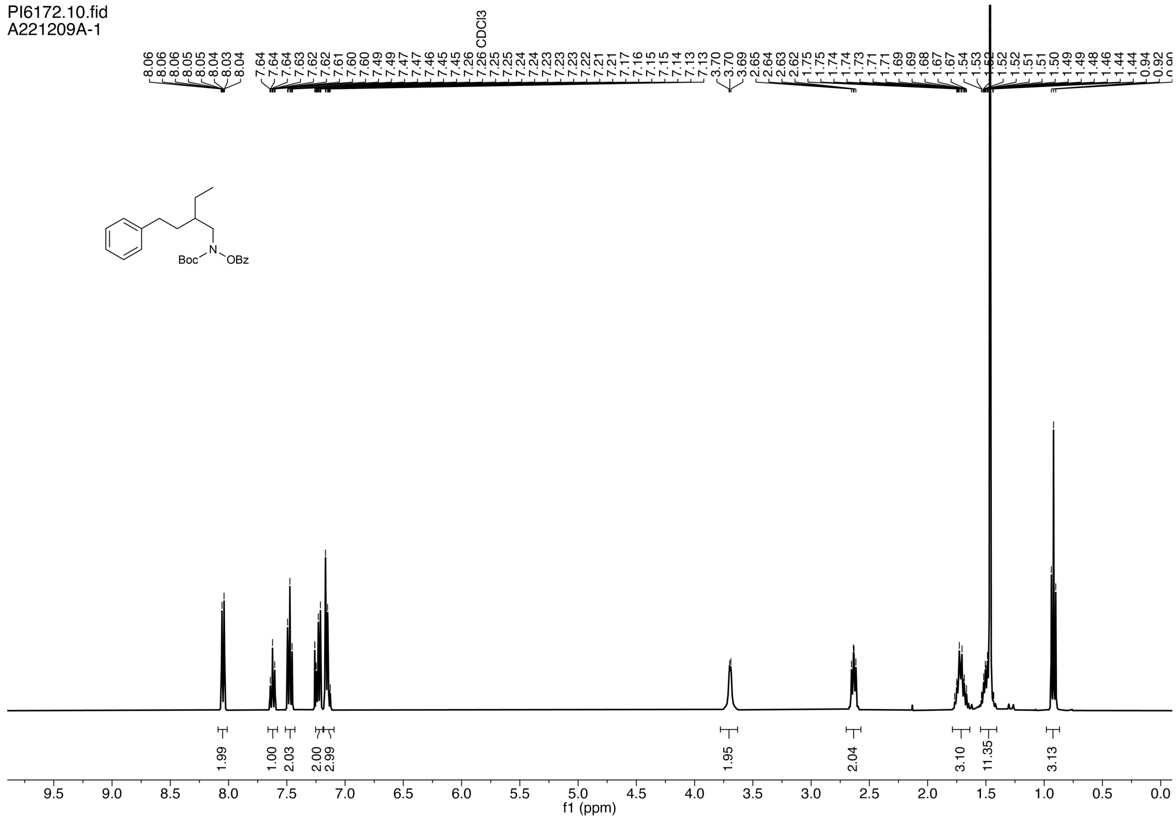

^{13}C NMR (100 MHz, $CDCl_3$)

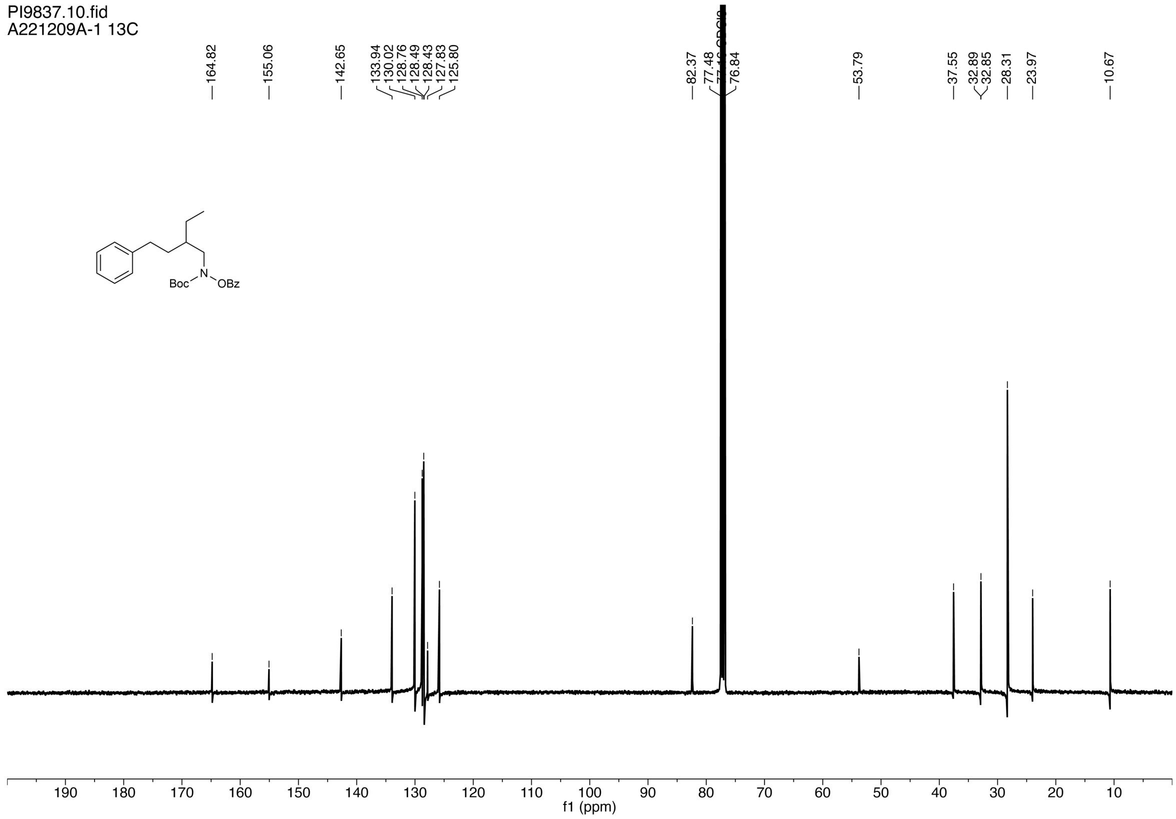

***tert*-Butyl hydroxy(4-methyl-2-phenethylpentyl)carbamate (S4)**:

^{1}H NMR (400 MHz, $CDCl_3$)

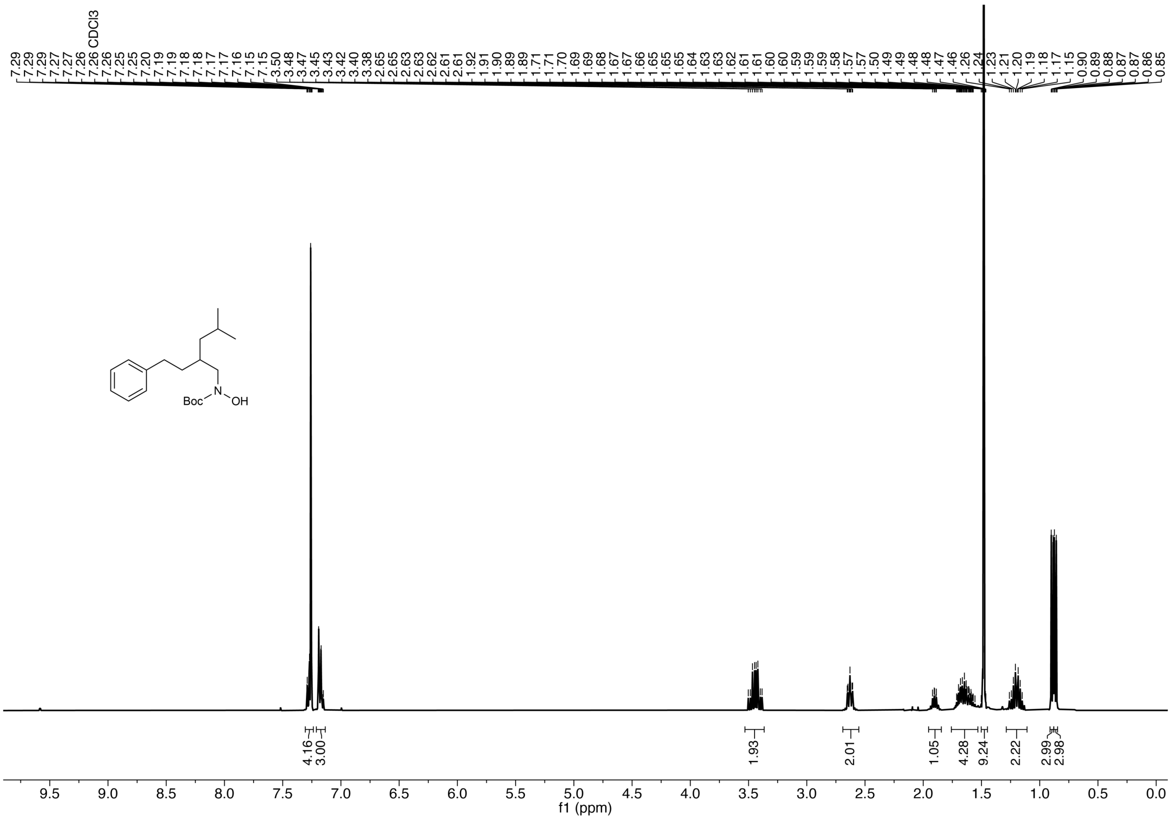

^{13}C NMR (100 MHz, $CDCl_3$)

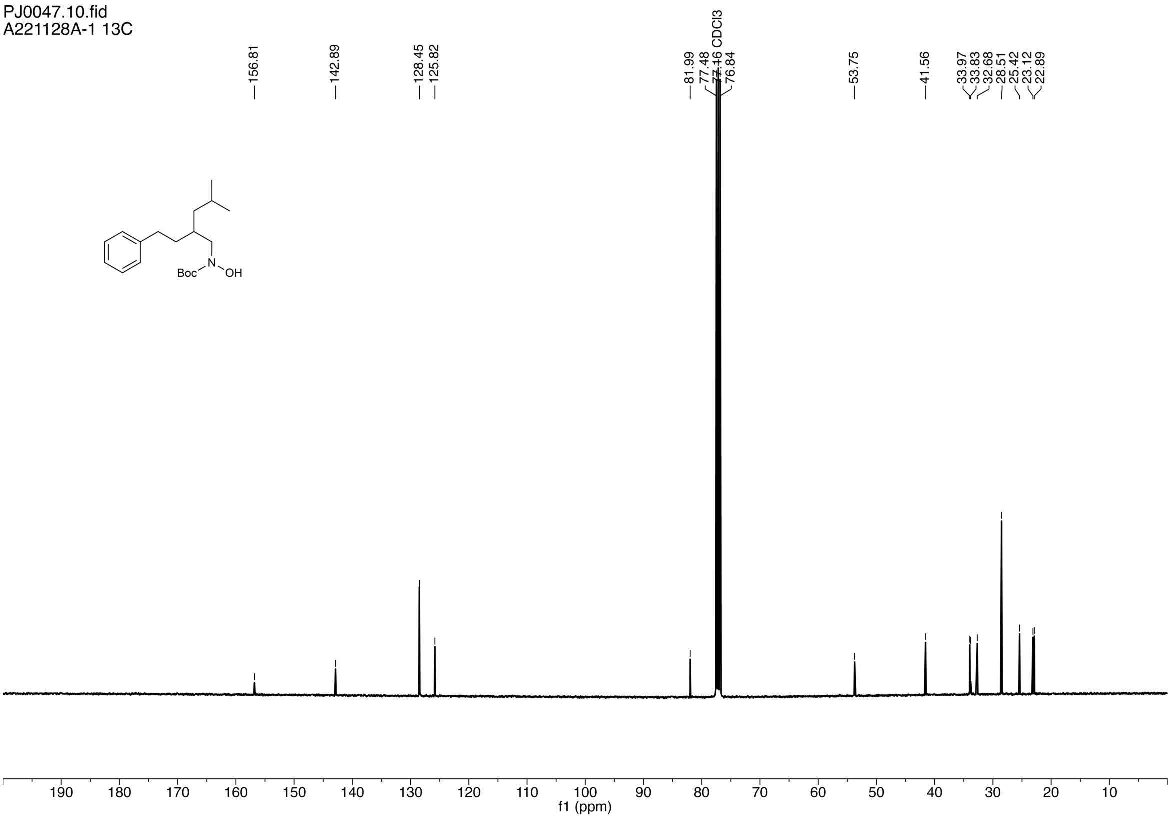

***tert*-Butyl hydroxy(2-phenethylpentyl)carbamate (S5)**:

^{1}H NMR (400 MHz, $CDCl_3$)

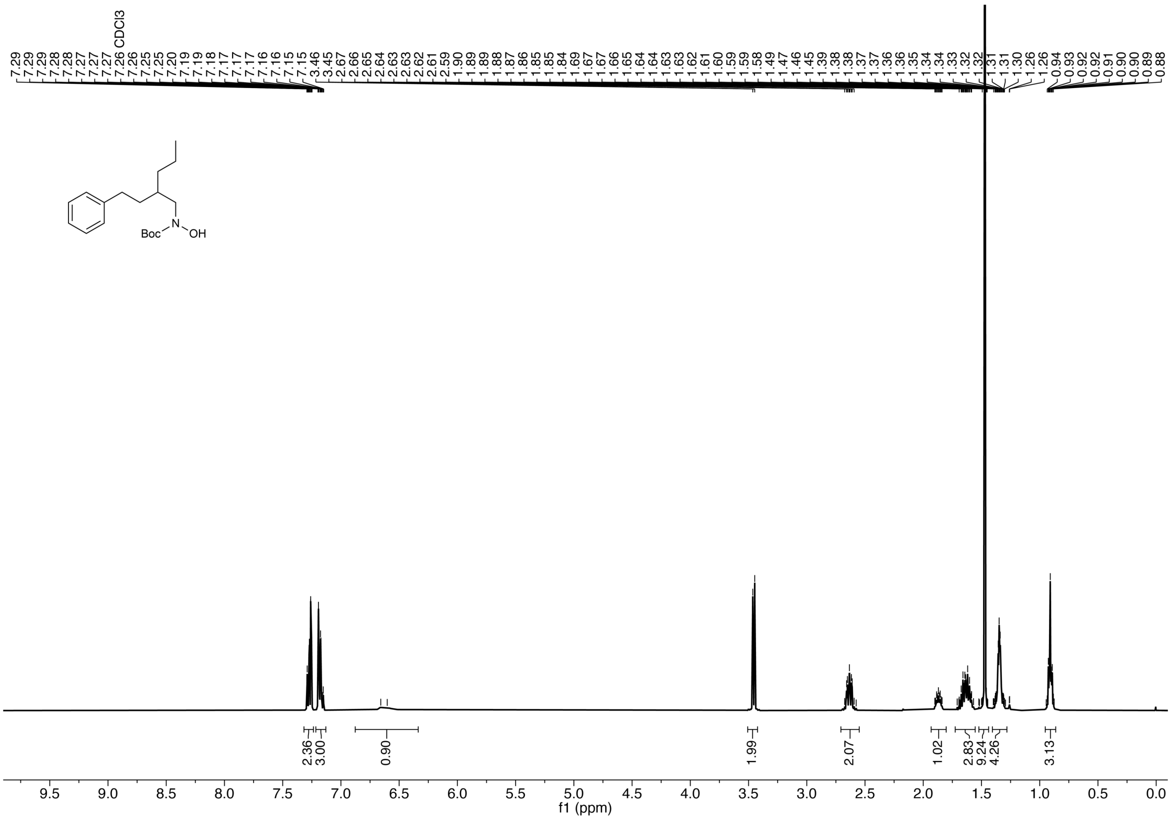

^{13}C NMR (100 MHz, $CDCl_3$)

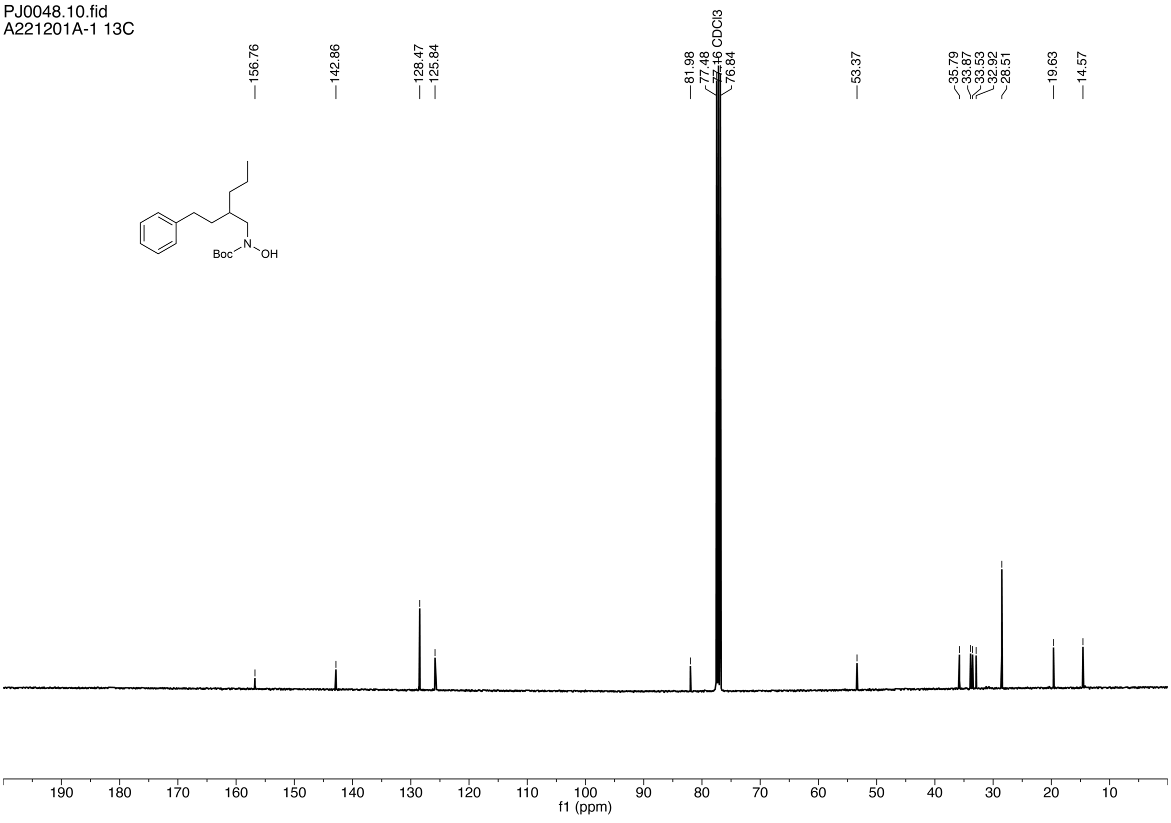

***tert*-Butyl (2-ethyl-4-phenylbutyl)(hydroxy)carbamate (S6)**:

^{1}H NMR (400 MHz, $CDCl_3$)

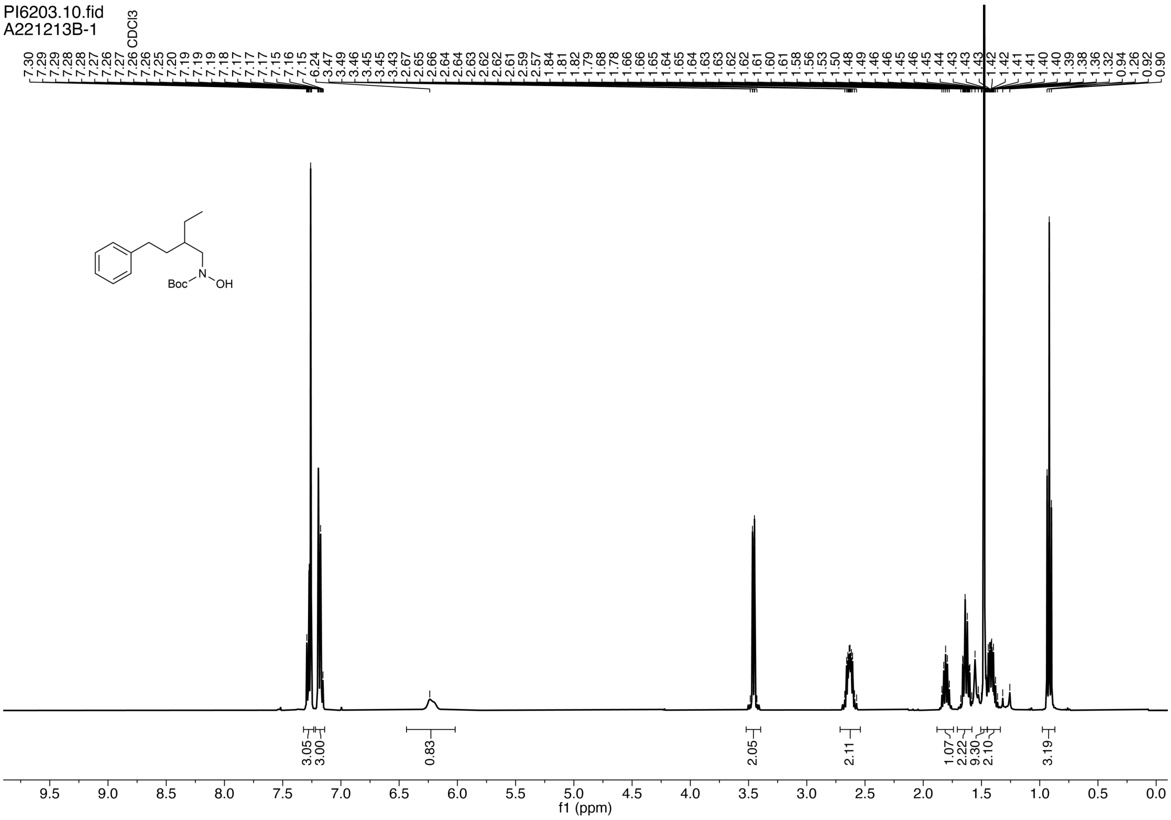

^{13}C NMR (100 MHz, $CDCl_3$)

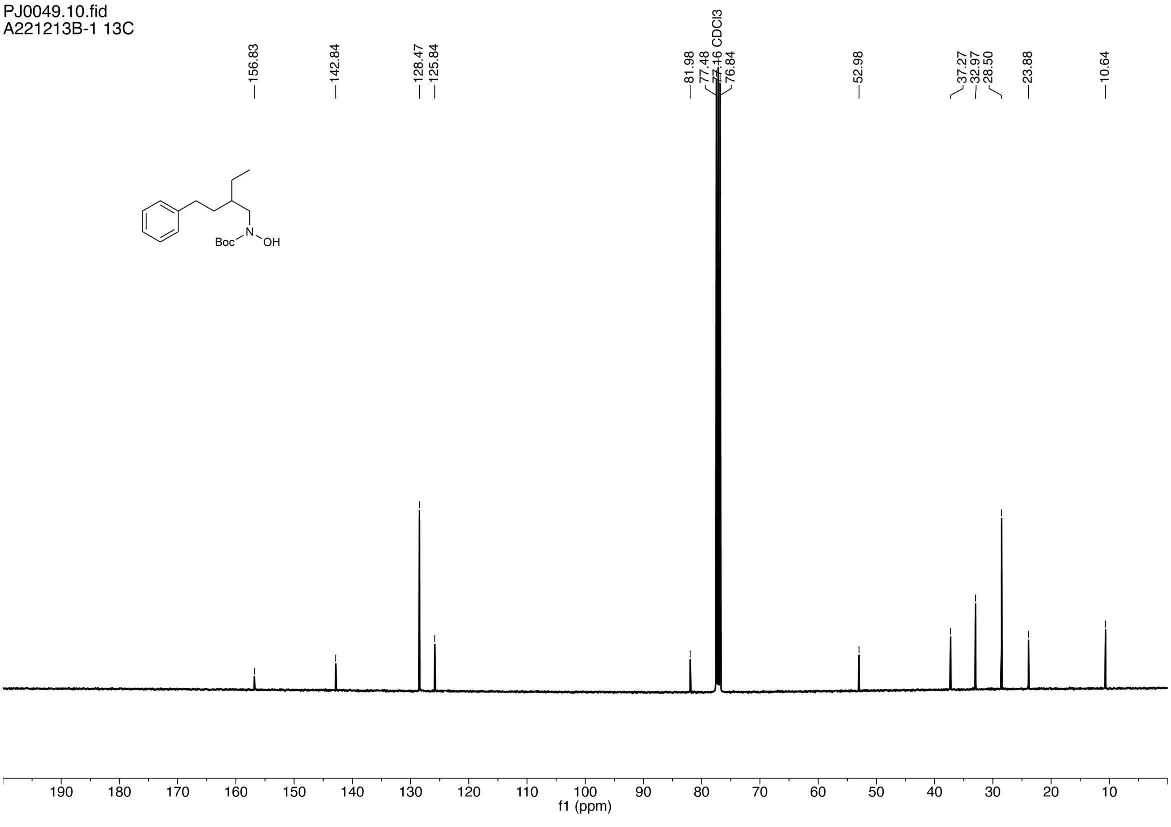

***O*-Benzoyl-*N*-(4-methyl-2-phenethylpentyl)hydroxylamine (11aA)**:

^{1}H NMR (400 MHz, $CDCl_3$)

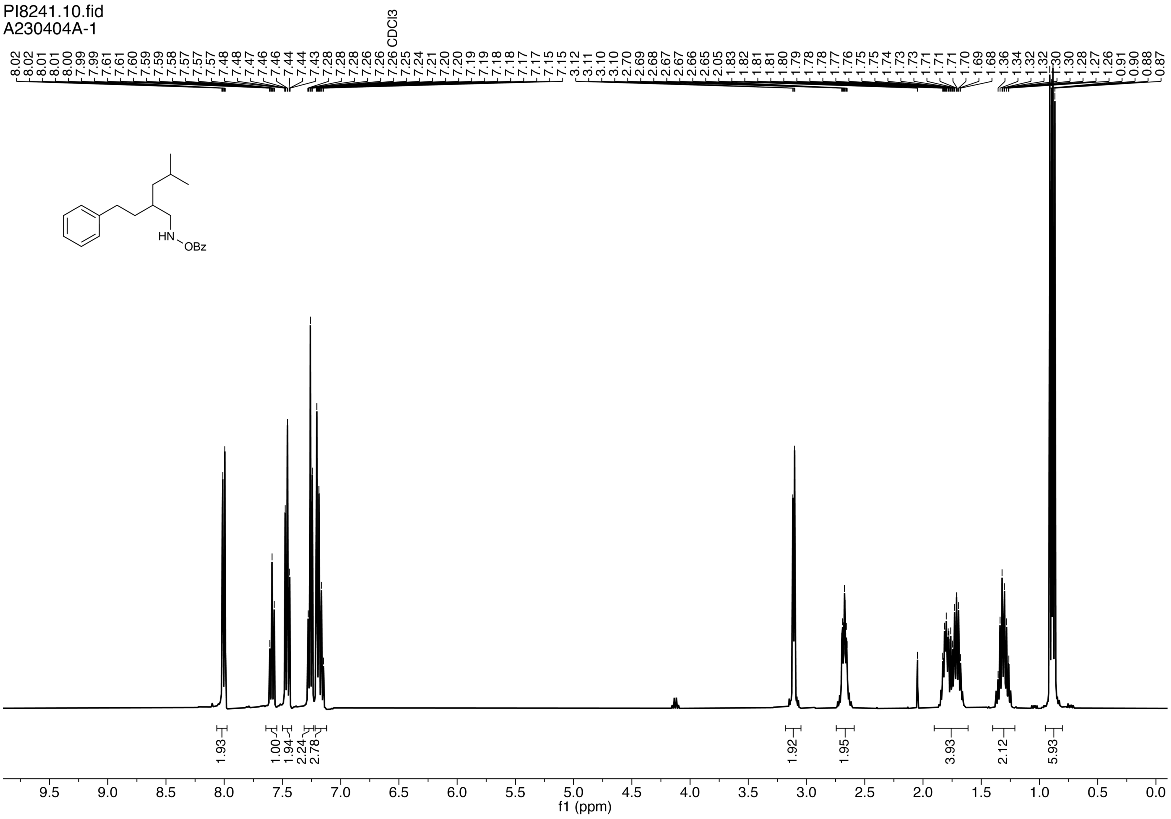

^{13}C NMR (100 MHz, $CDCl_3$)

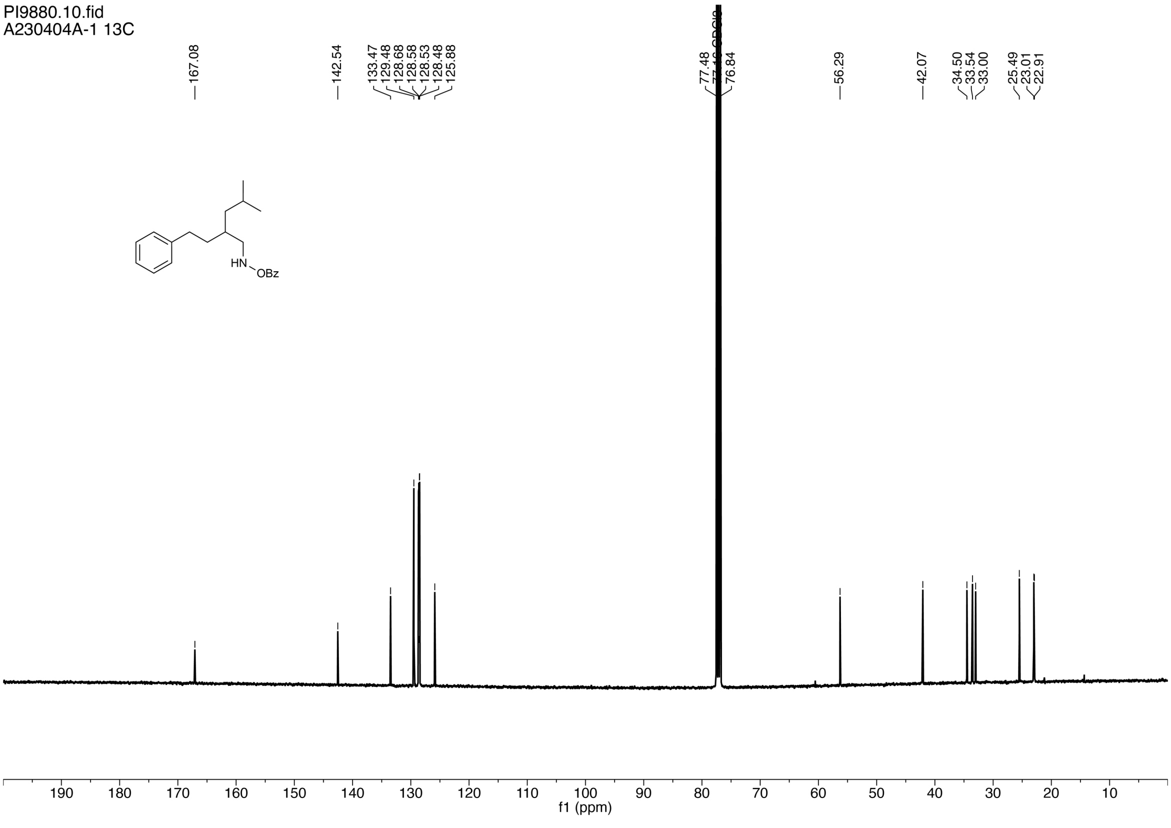

***O*-Benzoyl-*N*-(2-phenethylpentyl)hydroxylamine (11bA)**:

¹H NMR (400 MHz, $CDCl_3$)

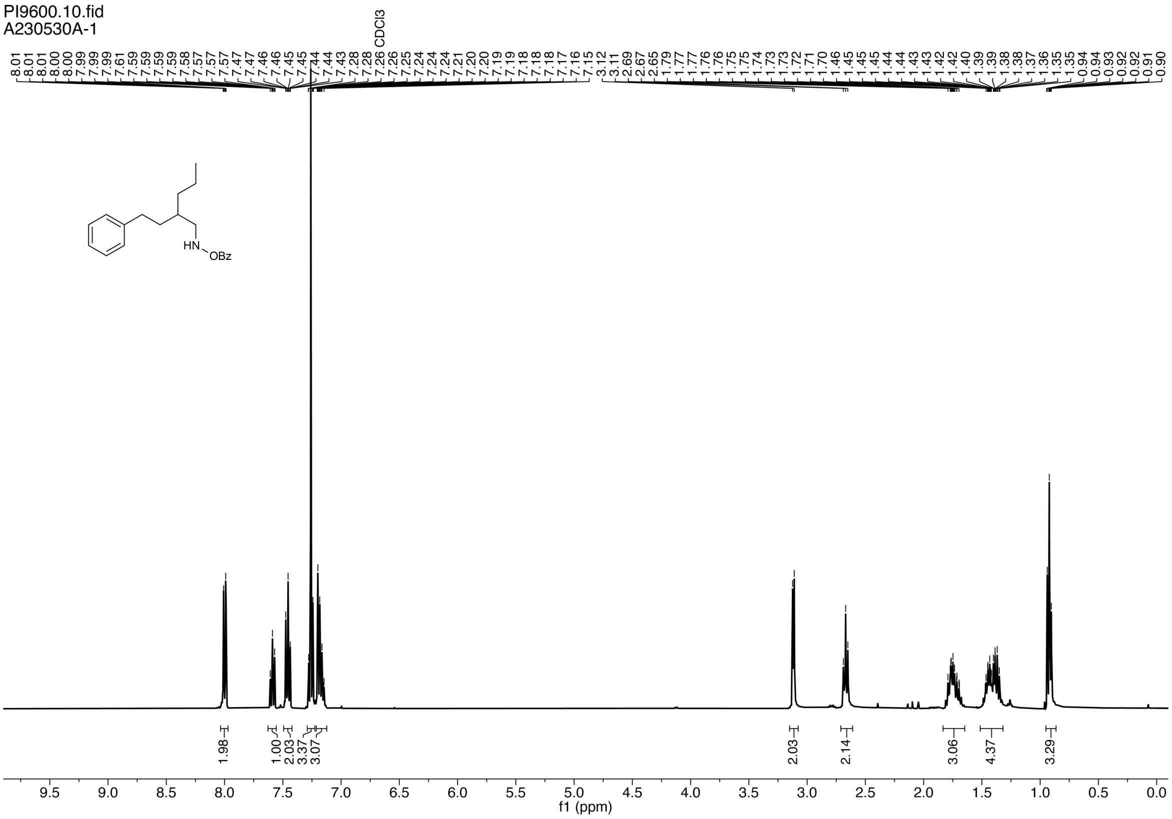

¹³C NMR (100 MHz, $CDCl_3$)

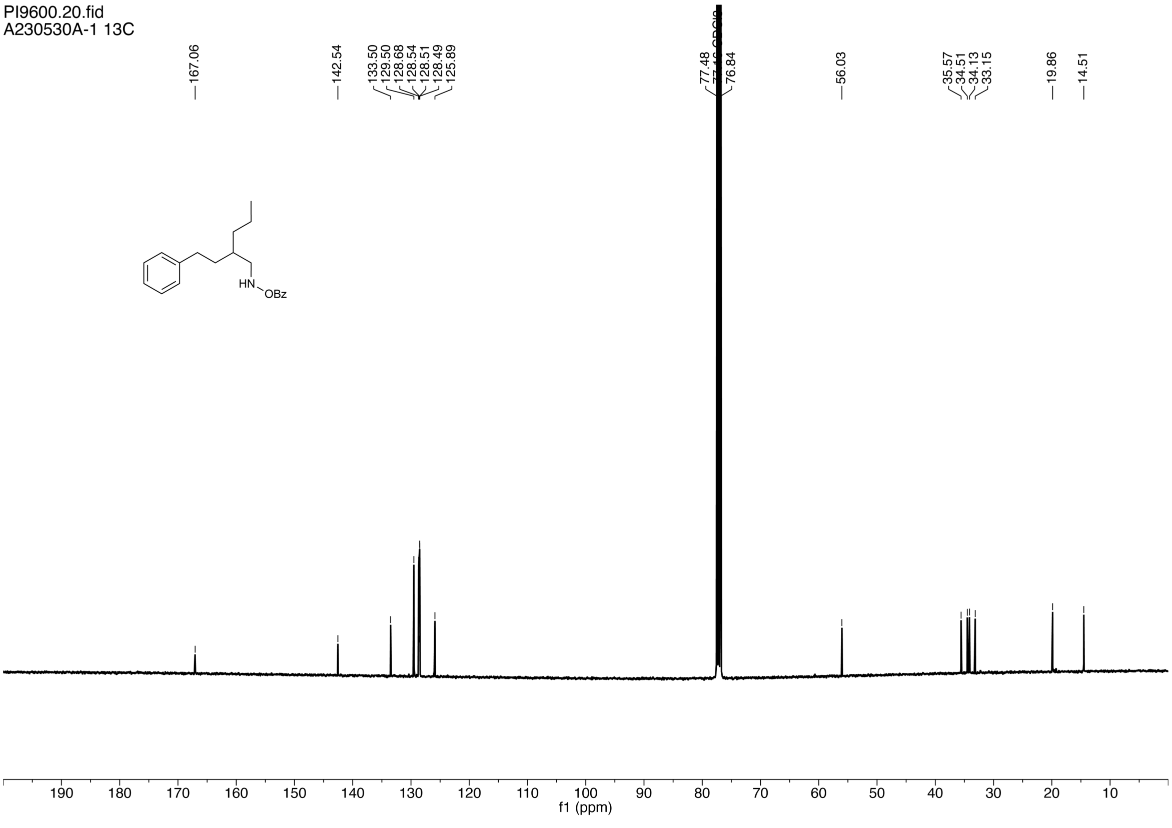

***O*-Benzoyl-*N*-(2-ethyl-4-phenylbutyl)hydroxylamine (11cA)**:

^{1}H NMR (400 MHz, $CDCl_3$)

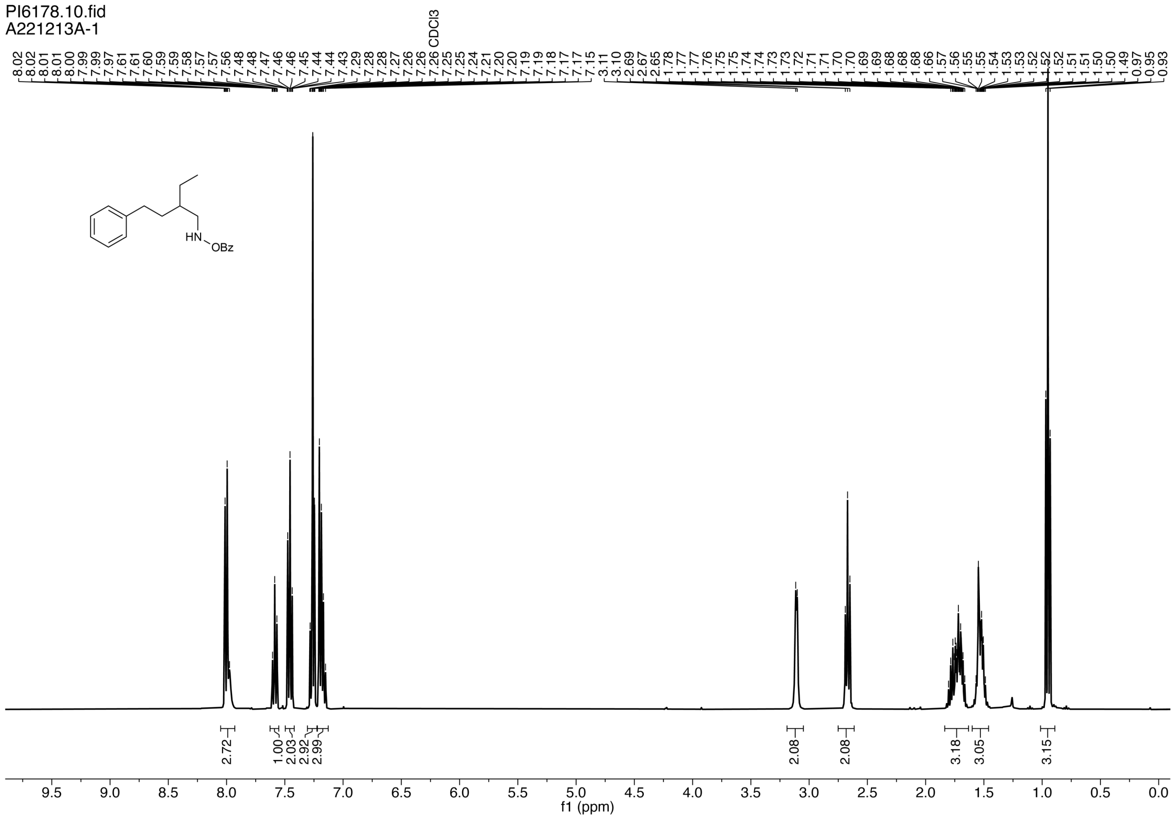

^{13}C NMR (100 MHz, $CDCl_3$)

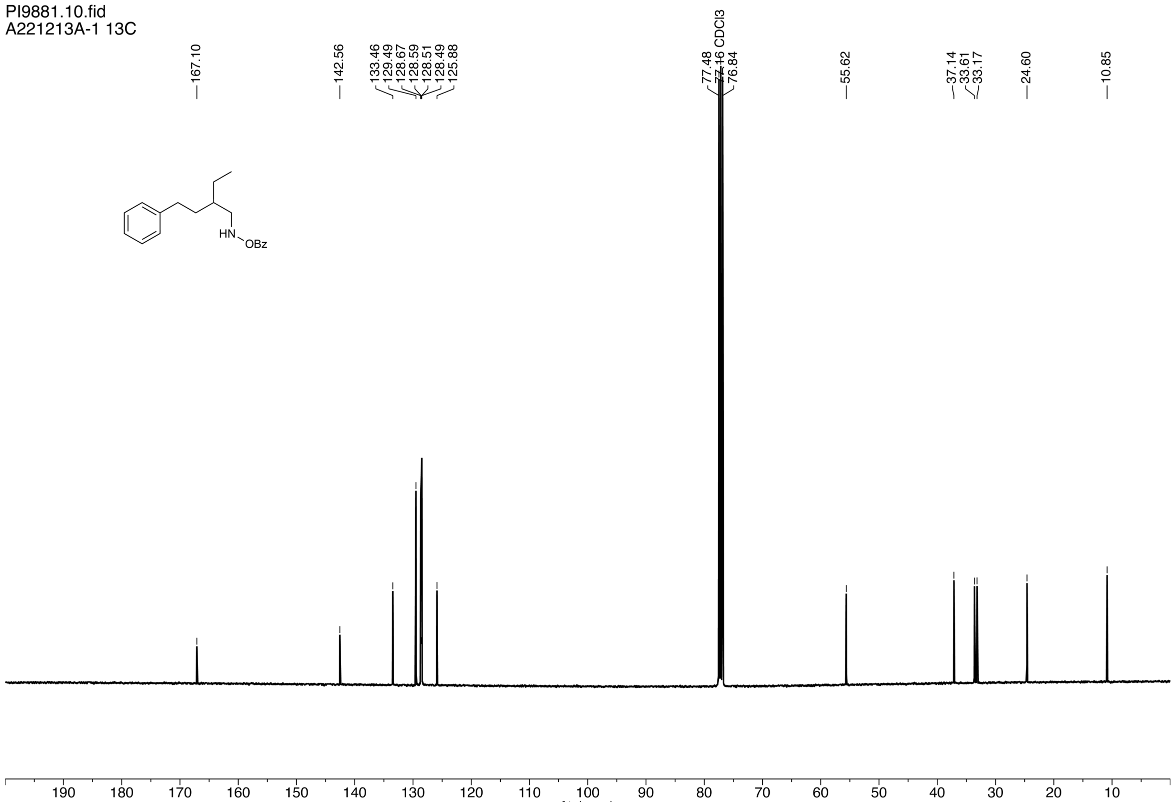

***tert*-Butyl (4-methyl-2-phenethylpentyl)(tosyloxy)carbamate (11aB)**:

^{1}H NMR (400 MHz, $CDCl_3$)

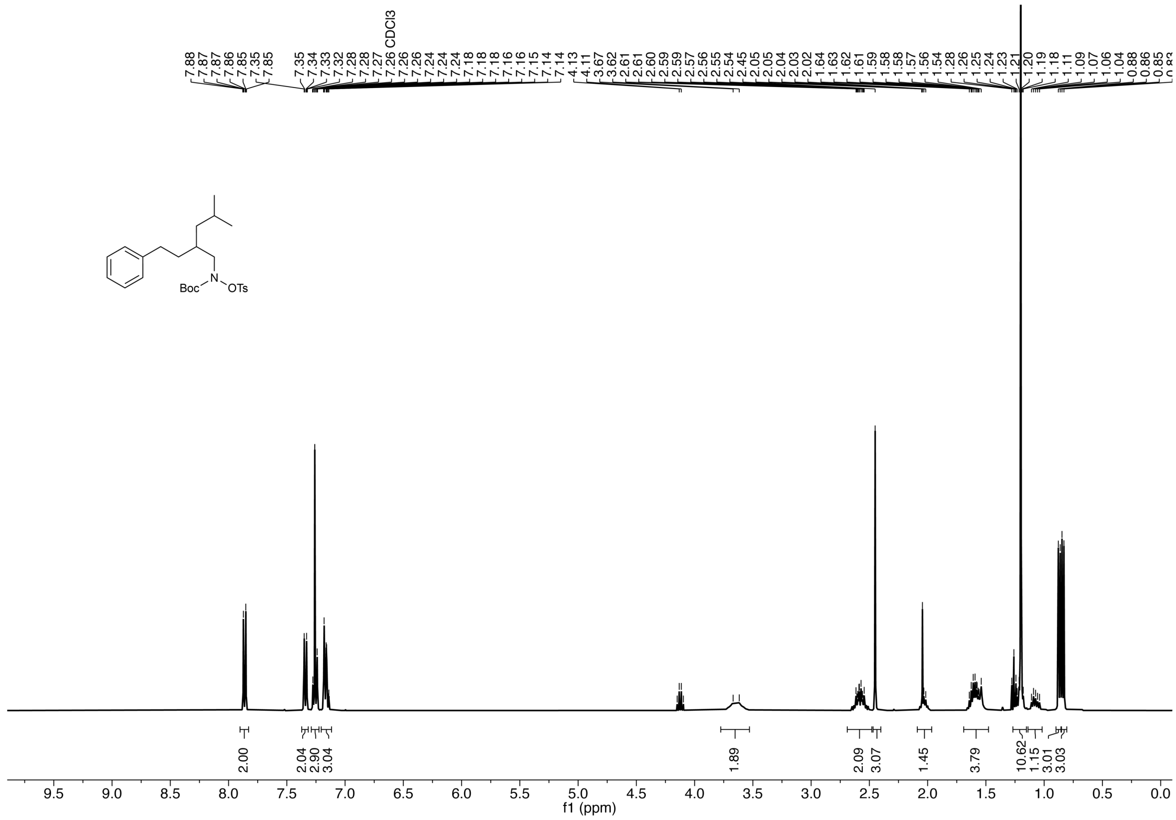

^{13}C NMR (100 MHz, $CDCl_3$)

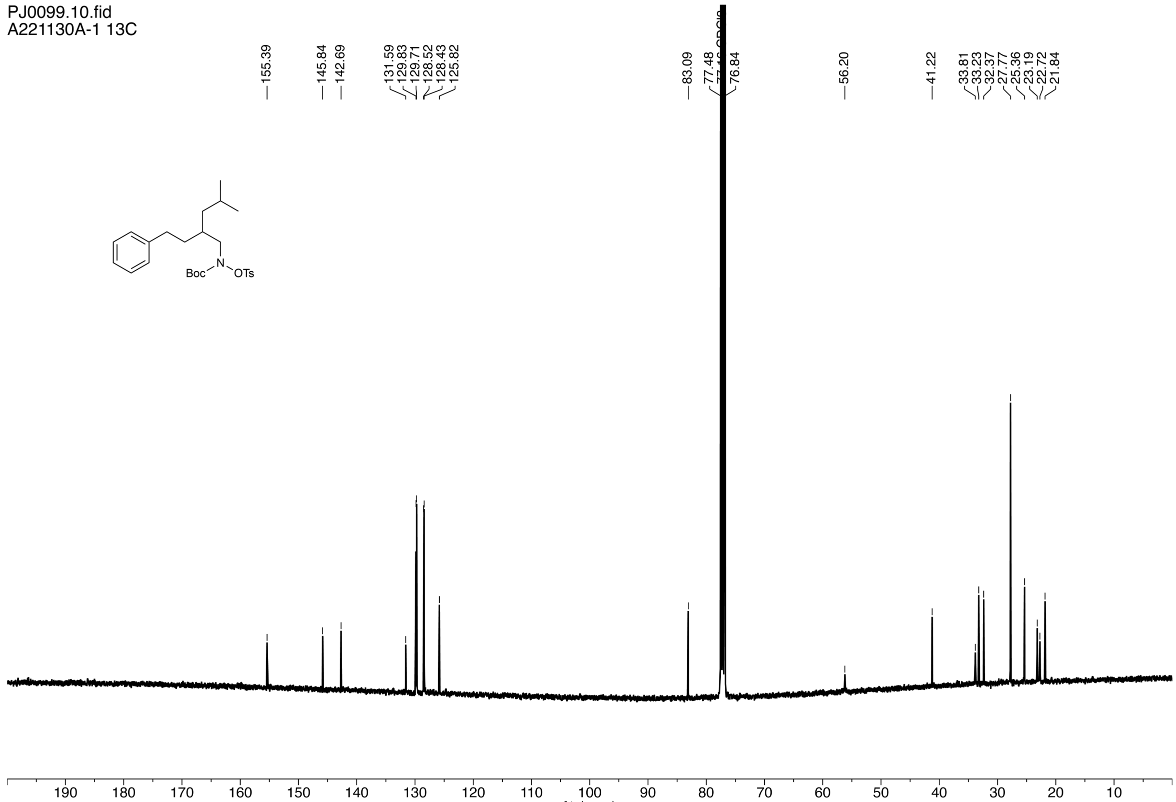

***tert*-Butyl (2-phenethylpentyl)(tosyloxy)carbamate (11bB)**:

^{1}H NMR (400 MHz, $CDCl_3$)

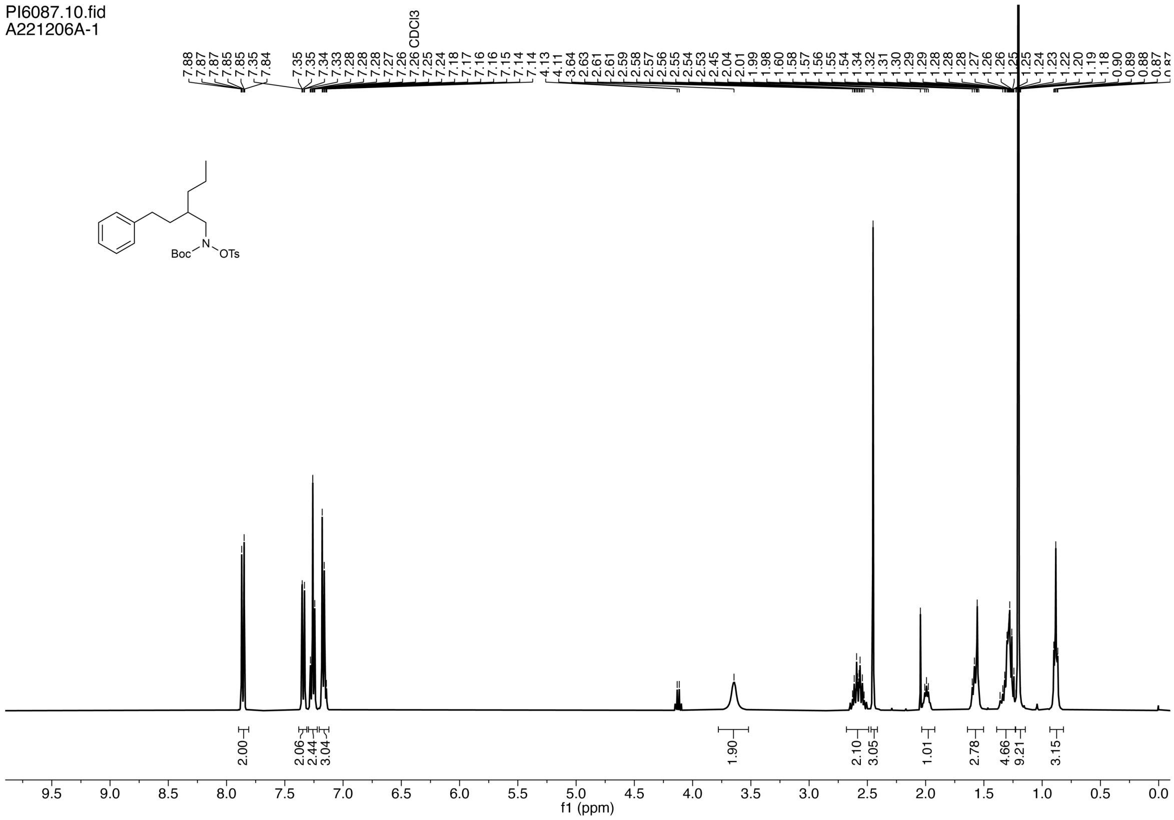

^{13}C NMR (100 MHz, $CDCl_3$)

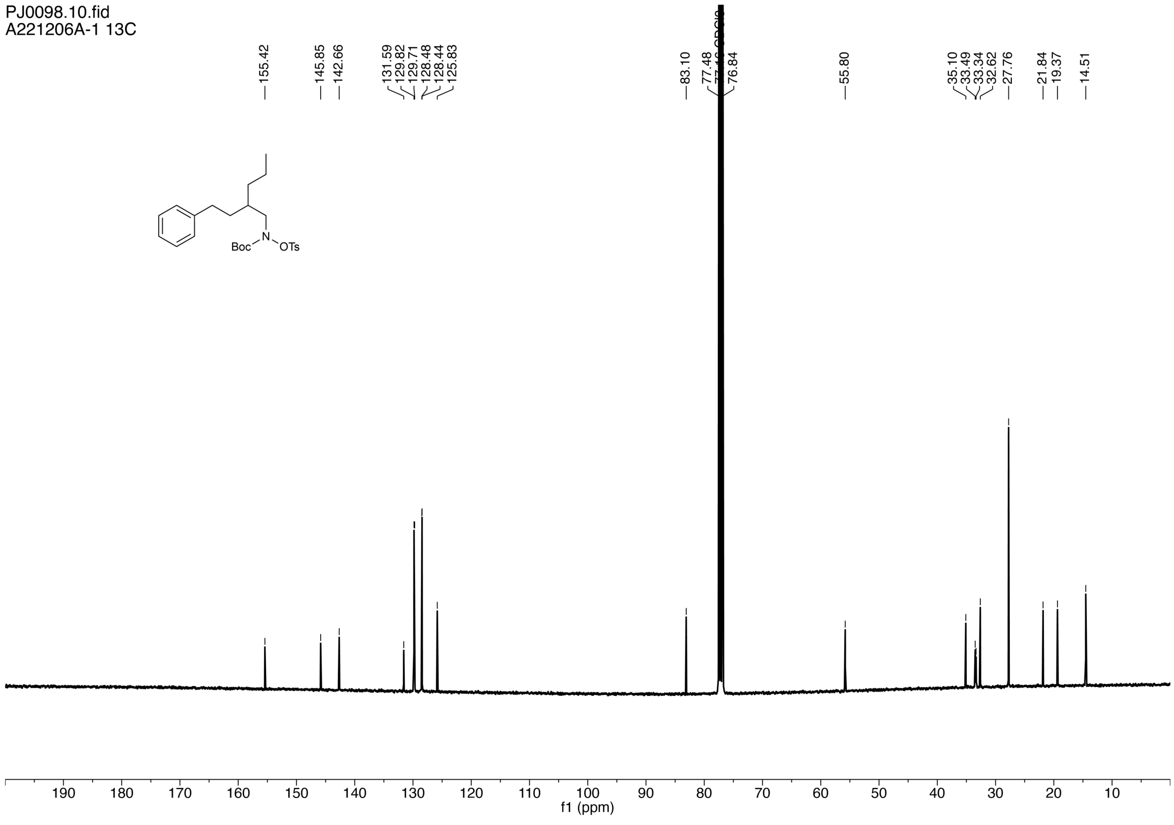

***tert*-Butyl (2-ethyl-4-phenylbutyl)(tosyloxy)carbamate (11cB)**:

^{1}H NMR (400 MHz, $CDCl_3$)

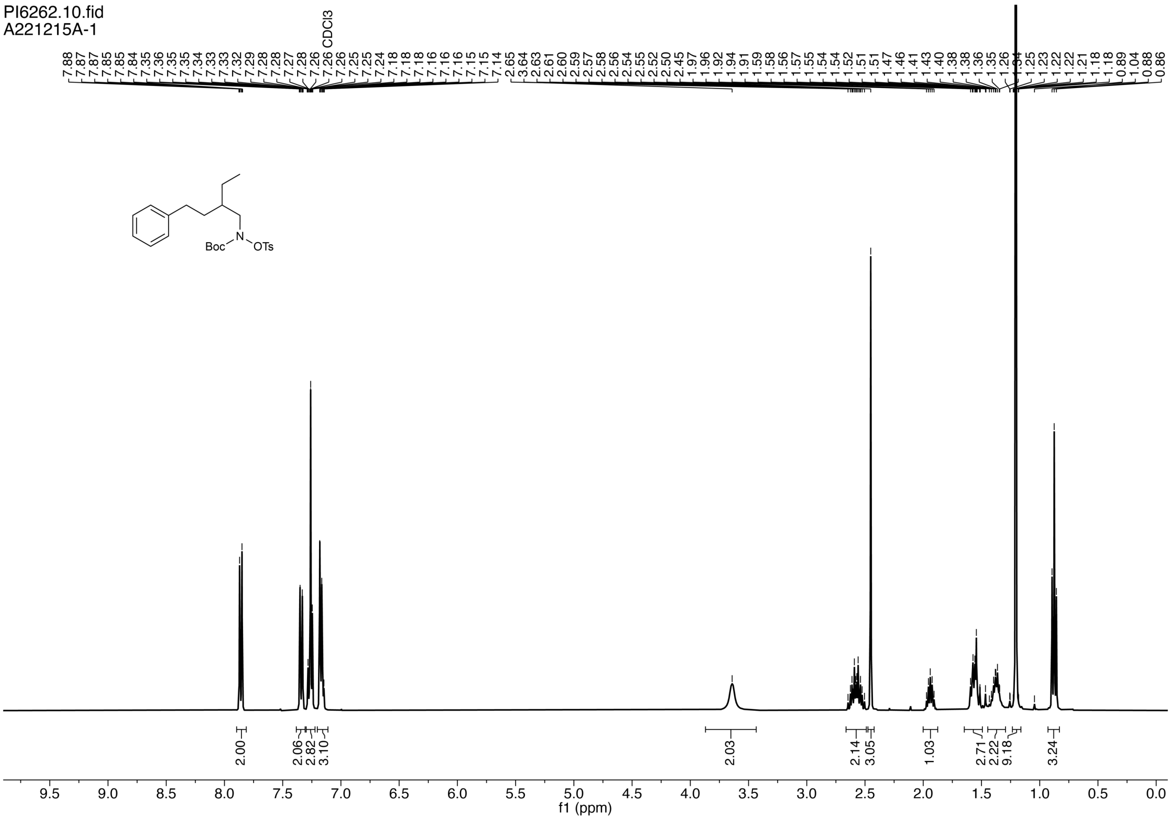

^{13}C NMR (100 MHz, $CDCl_3$)

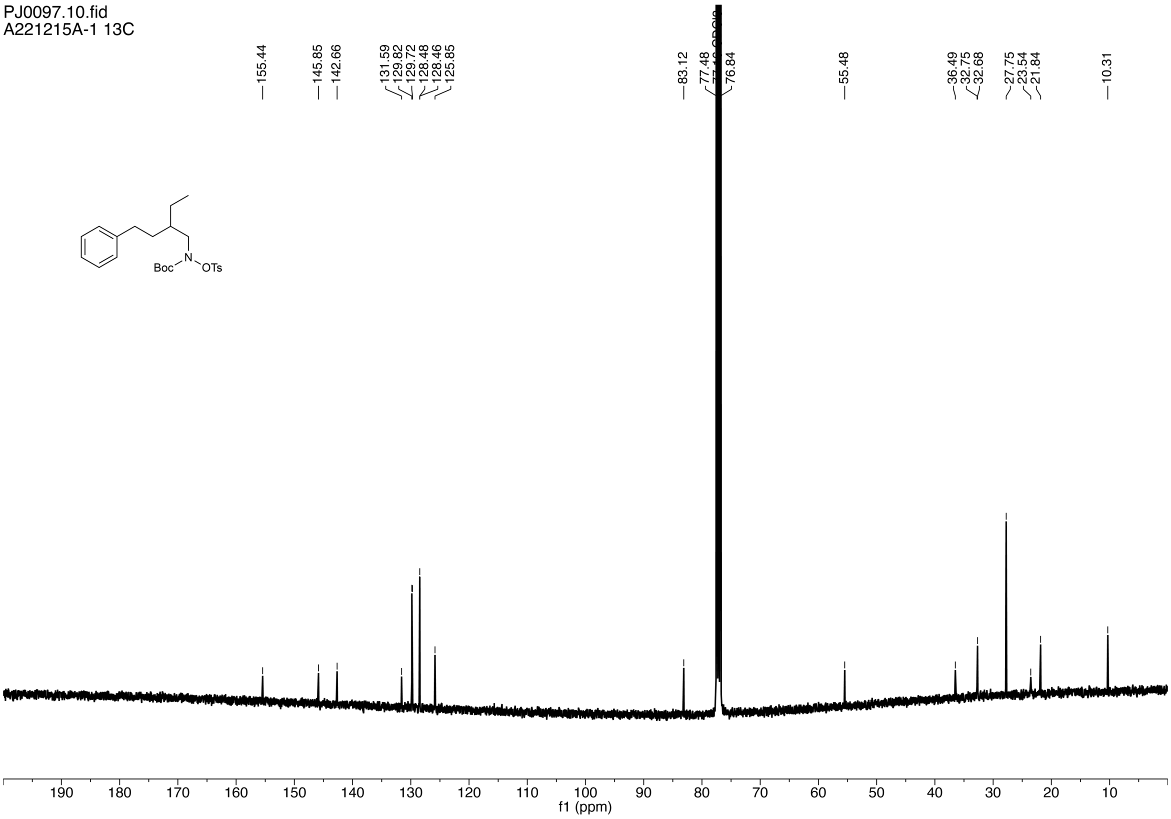

***tert*-Butyl (4-(4-fluorophenyl)butyl-4-*d*)(hydroxy)carbamate (S7):**

^{1}H NMR (400 MHz, $CDCl_3$)

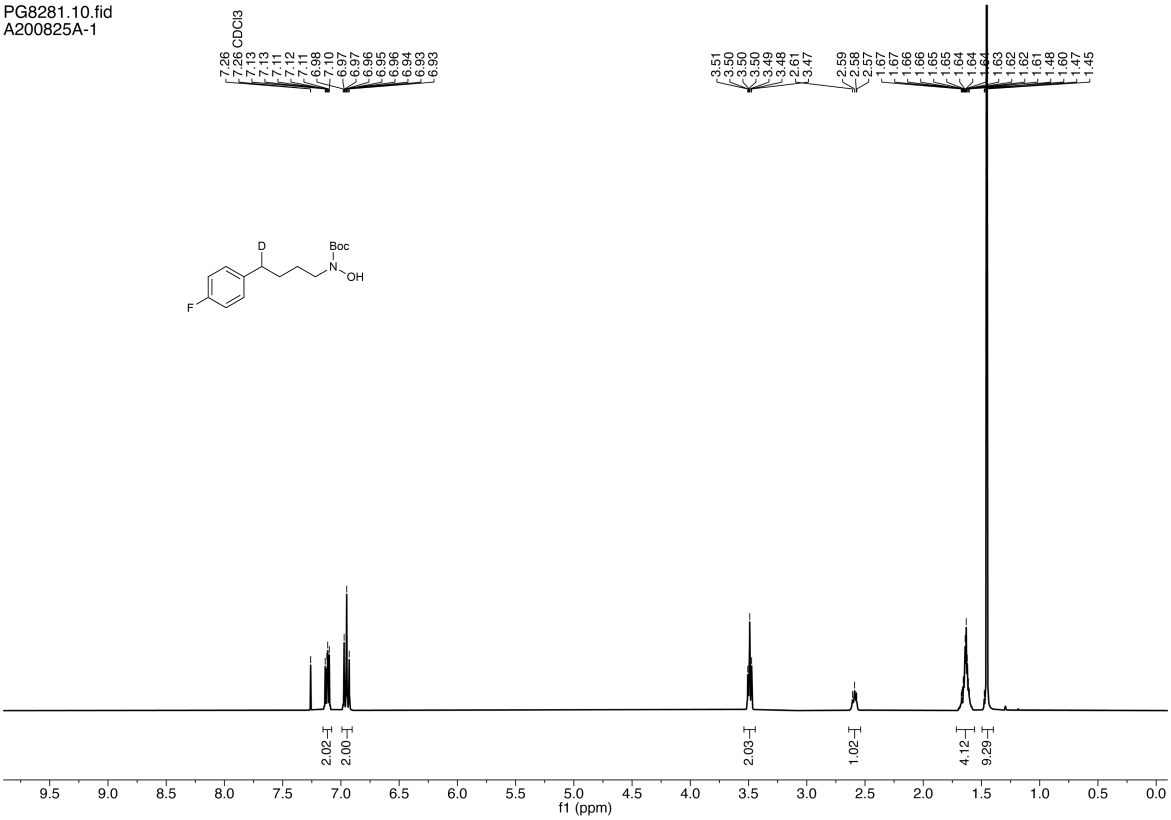

^{13}C NMR (100 MHz, $CDCl_3$)

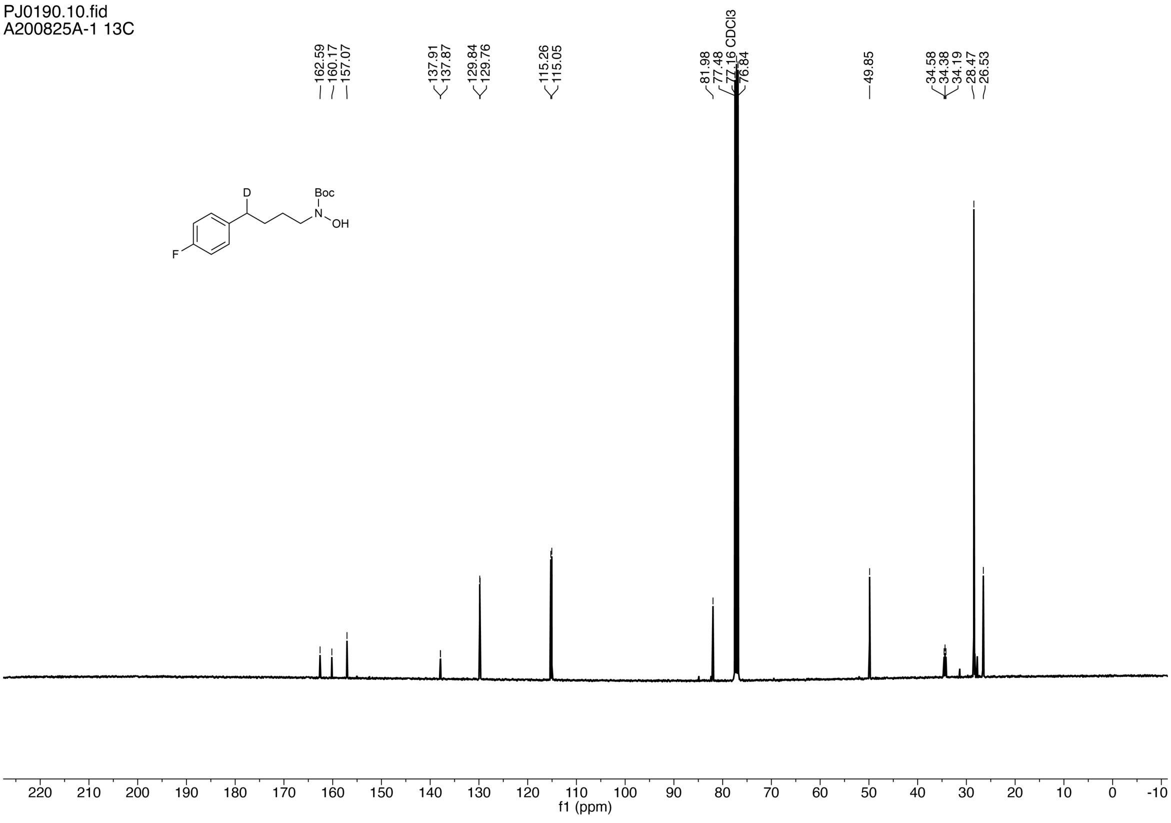

^{19}F NMR (376 MHz, $CDCl_3$)

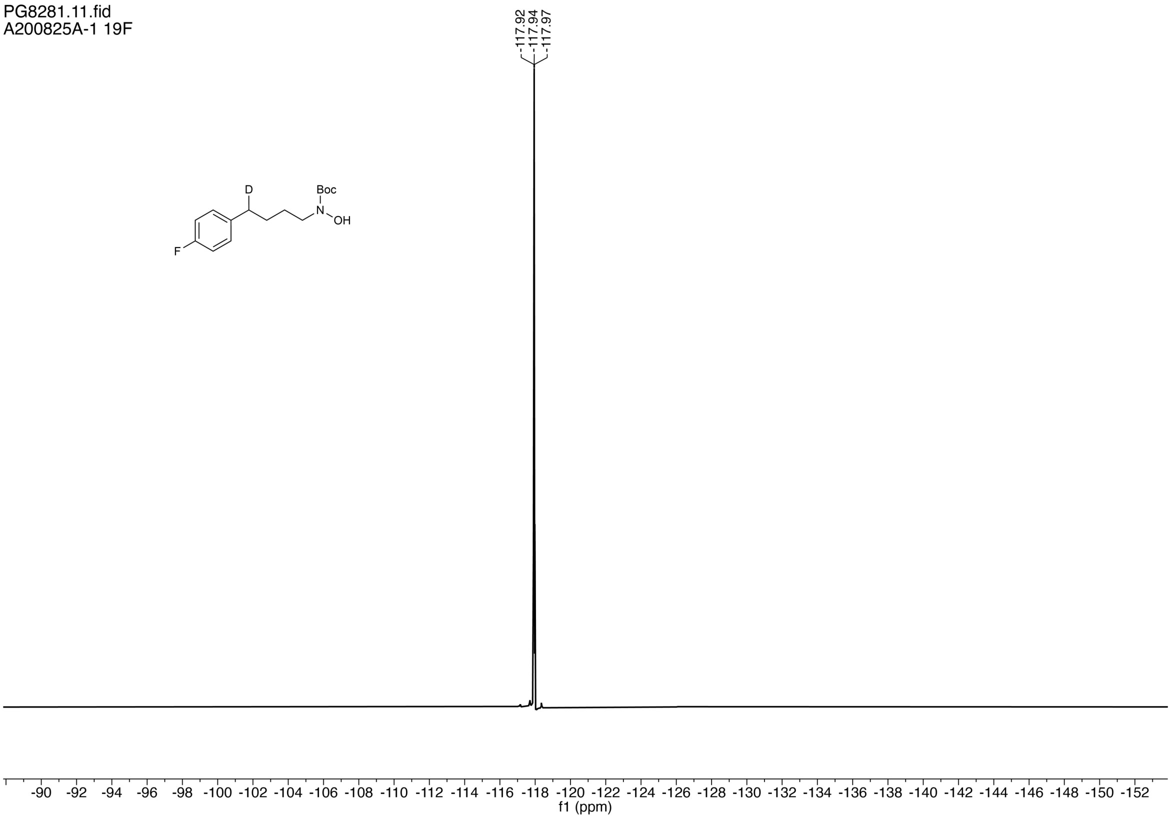

***tert*-Butyl (4-(4-fluorophenyl)butyl-4-*d*)((4-methoxybenzoyl)oxy)carbamate (Boc-17)**:

^{1}H NMR (400 MHz, $CDCl_3$)

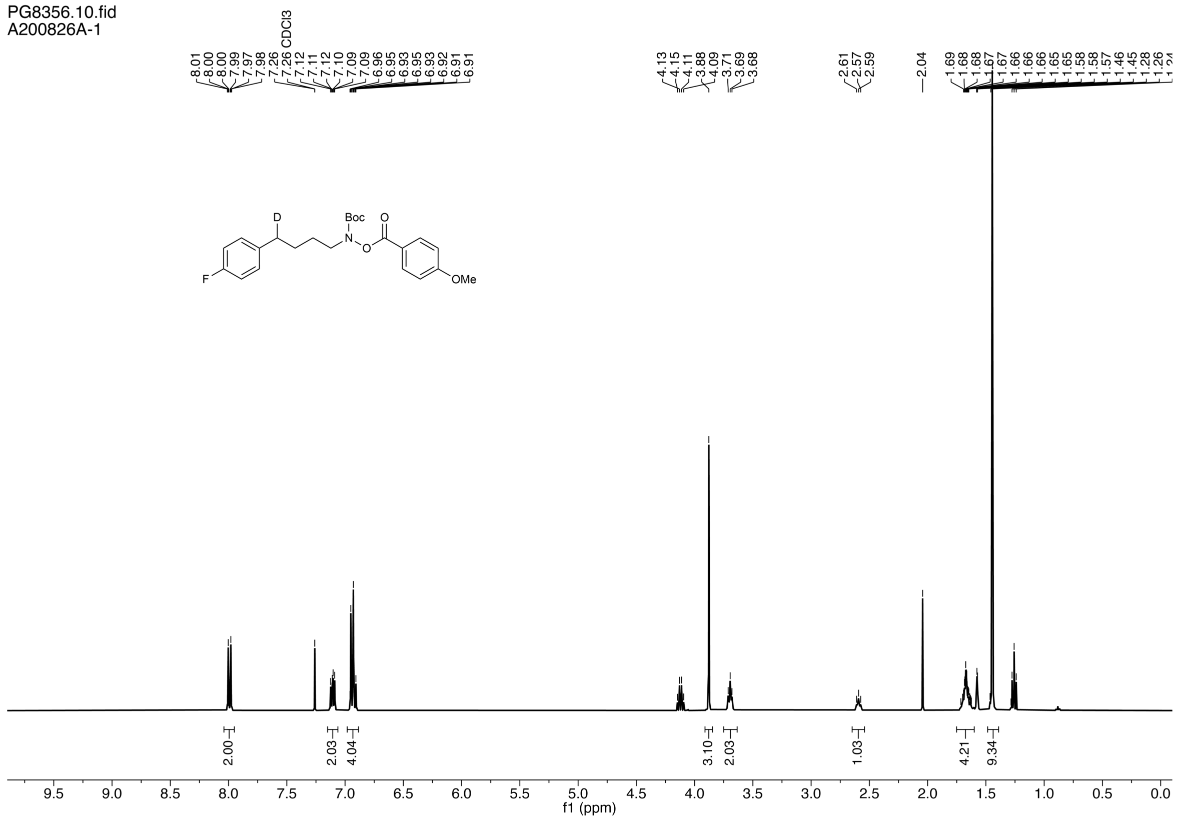

^{13}C NMR (100 MHz, $CDCl_3$)

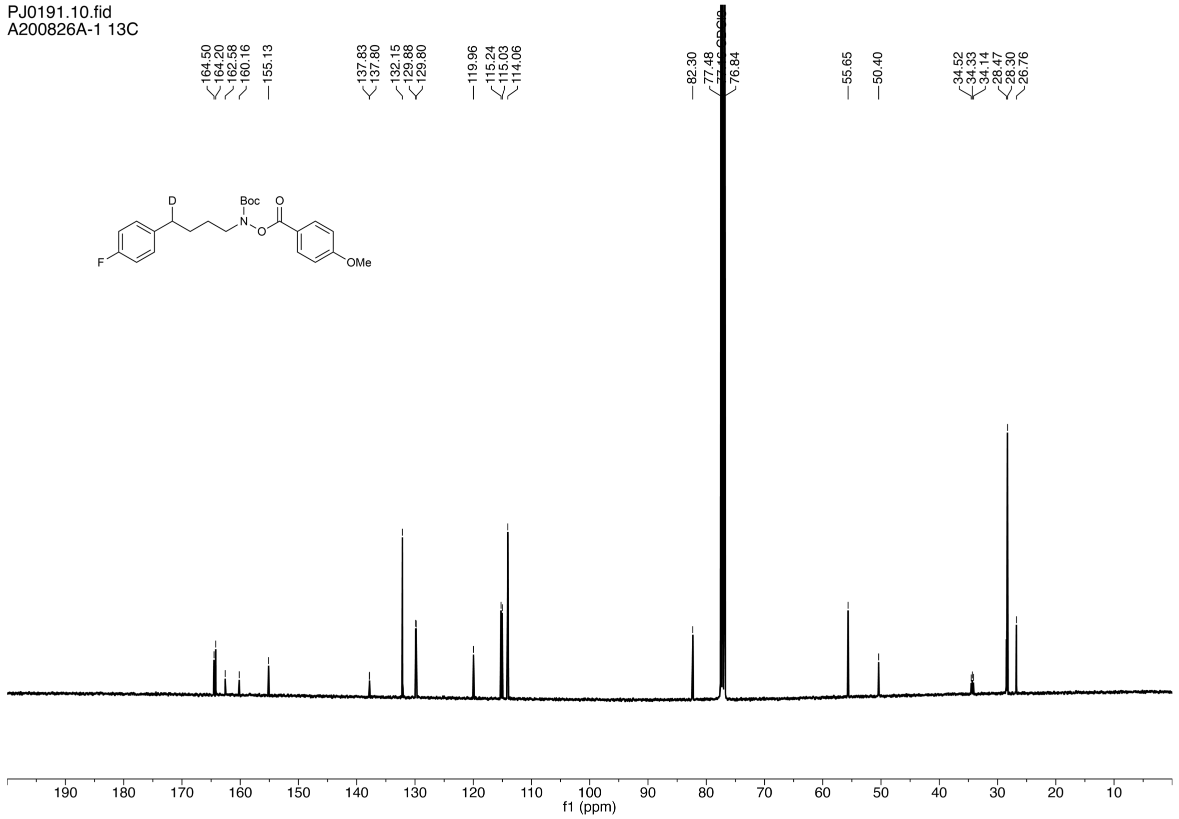

^{19}F NMR (376 MHz, $CDCl_3$)

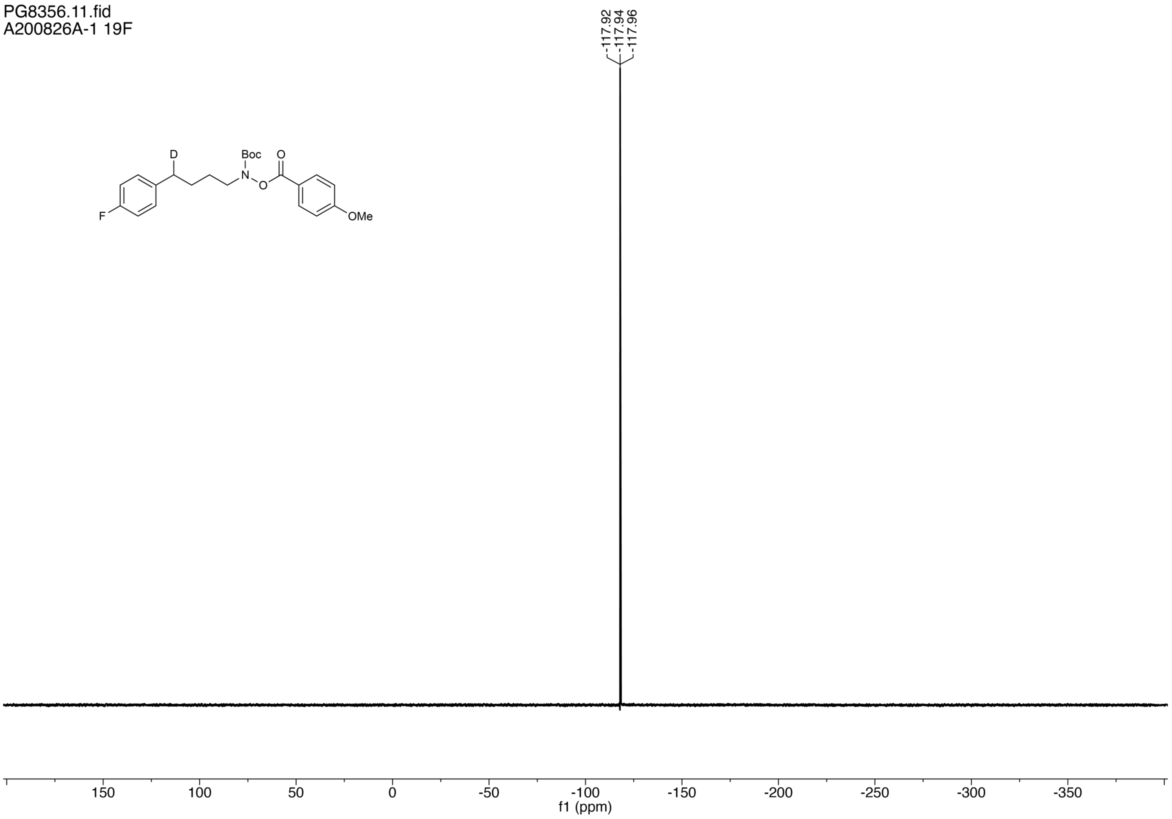

***N*-(4-(4-Fluorophenyl)butyl-4-*d*)-*O*-(4-methoxybenzoyl)hydroxylamine (17)**:

^{1}H NMR (400 MHz, $CDCl_3$)

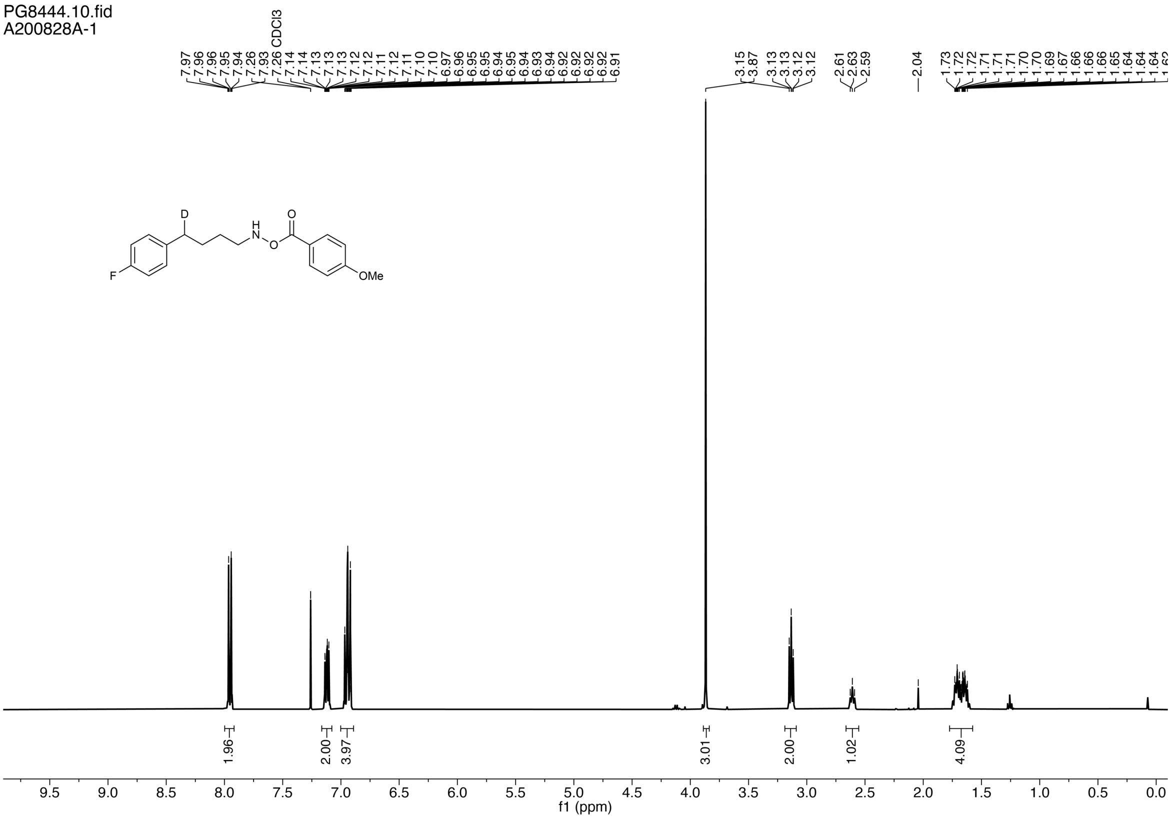

^{13}C NMR (100 MHz, $CDCl_3$)

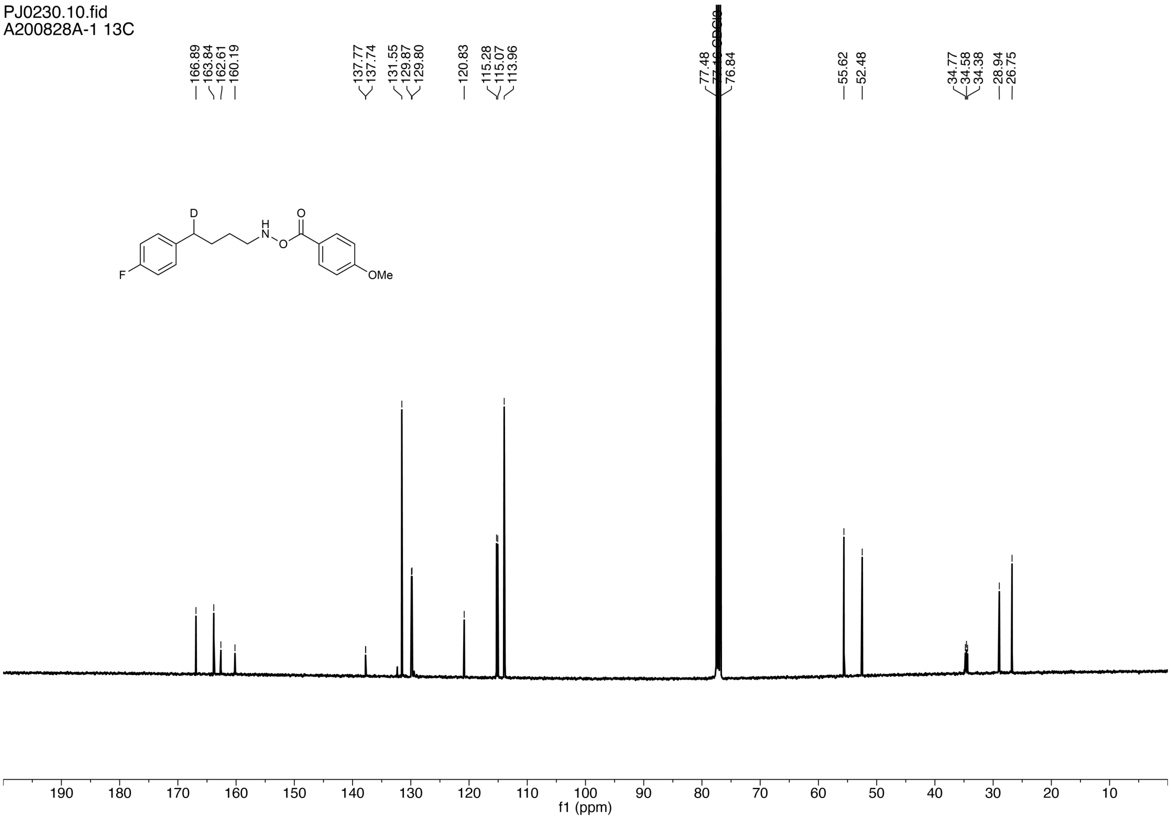

^{19}F NMR (376 MHz, $CDCl_3$)

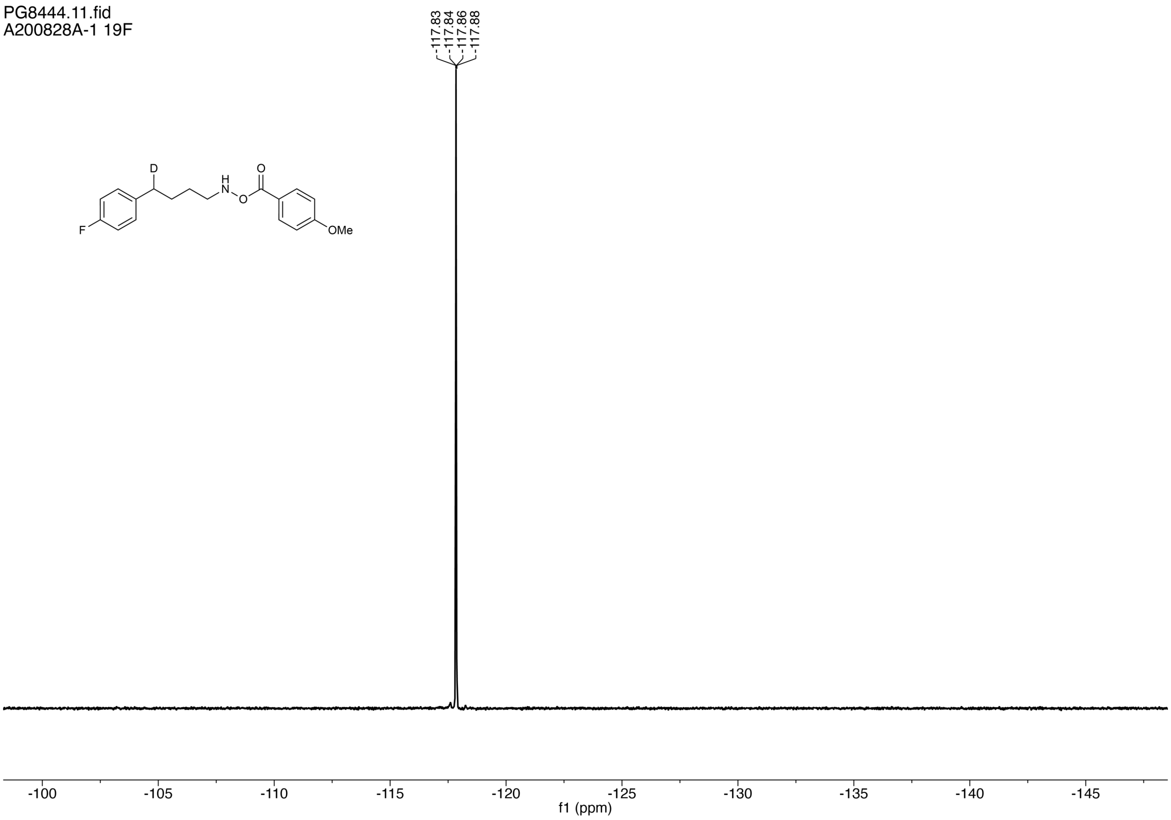

***tert*-Butyl ((4-cyanobenzoyl)oxy)(4-(4-fluorophenyl)butyl-4-*d*)carbamate (Boc-18)**:

¹H NMR (400 MHz, $CDCl_3$)

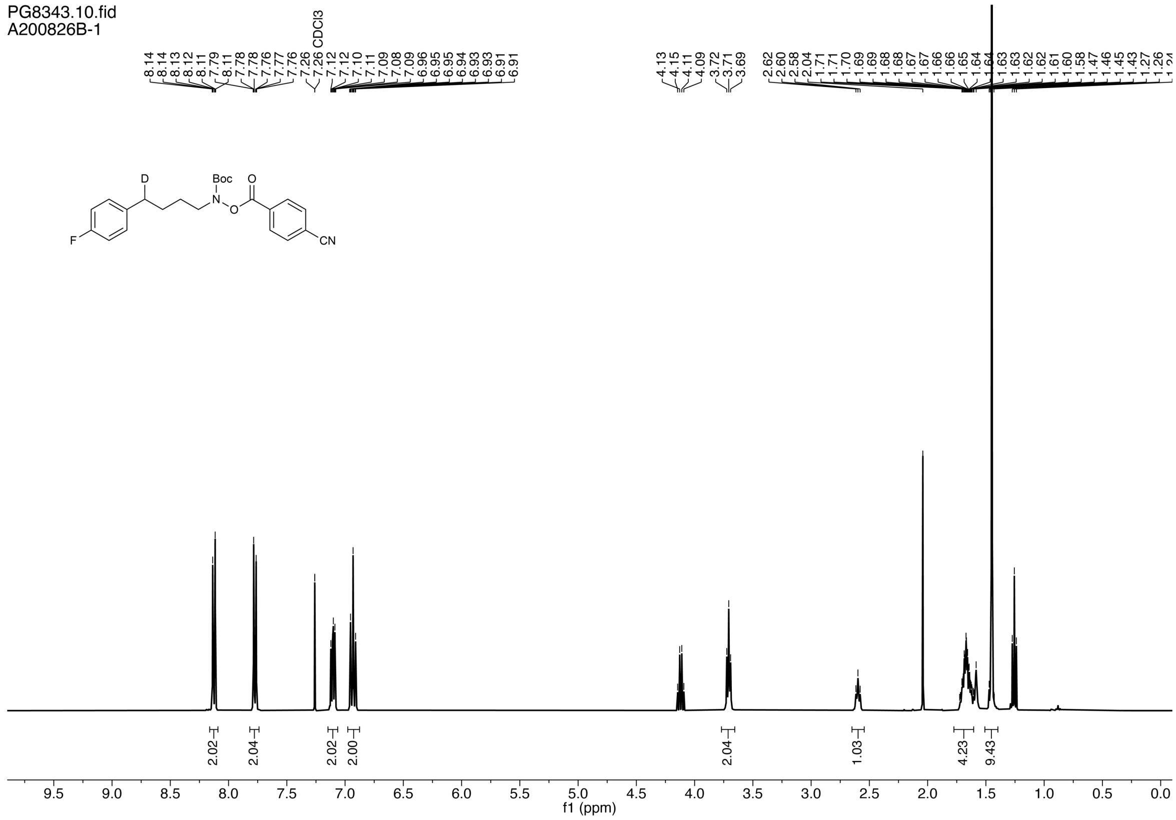

¹³C NMR (100 MHz, $CDCl_3$)

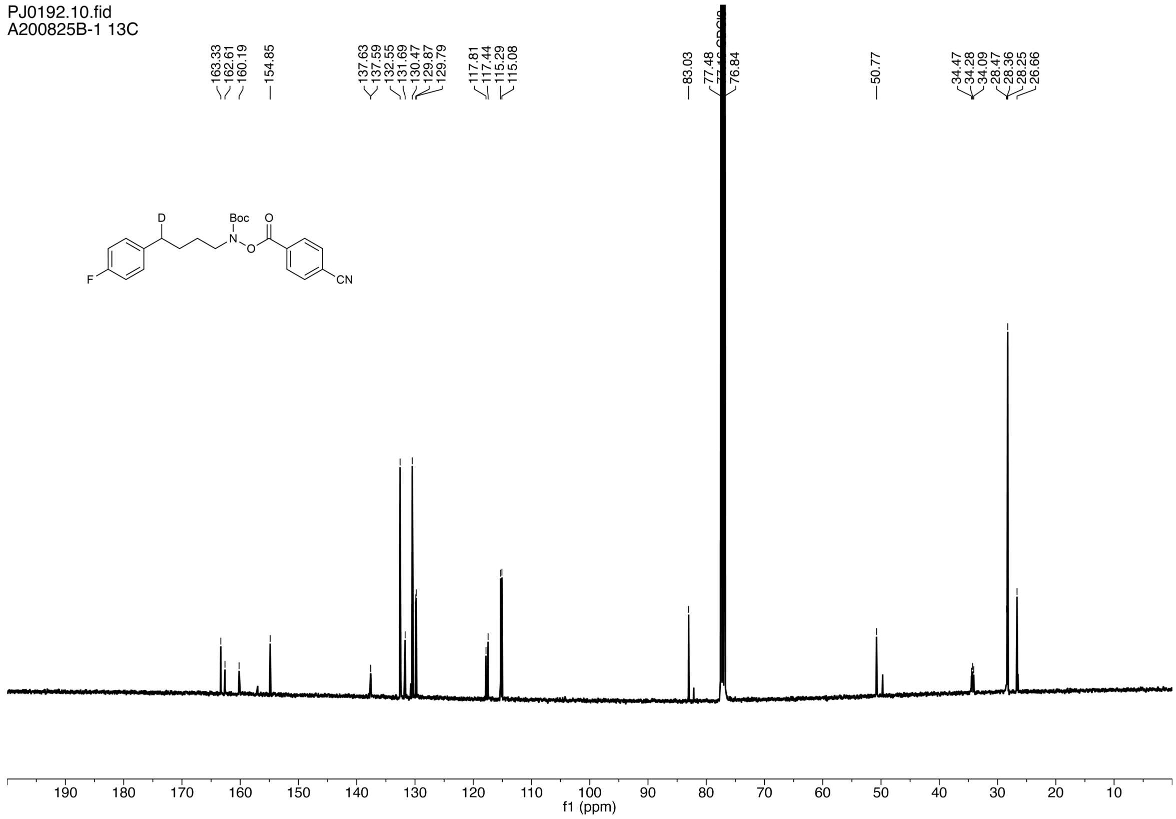

^{19}F NMR (376 MHz, $CDCl_3$)

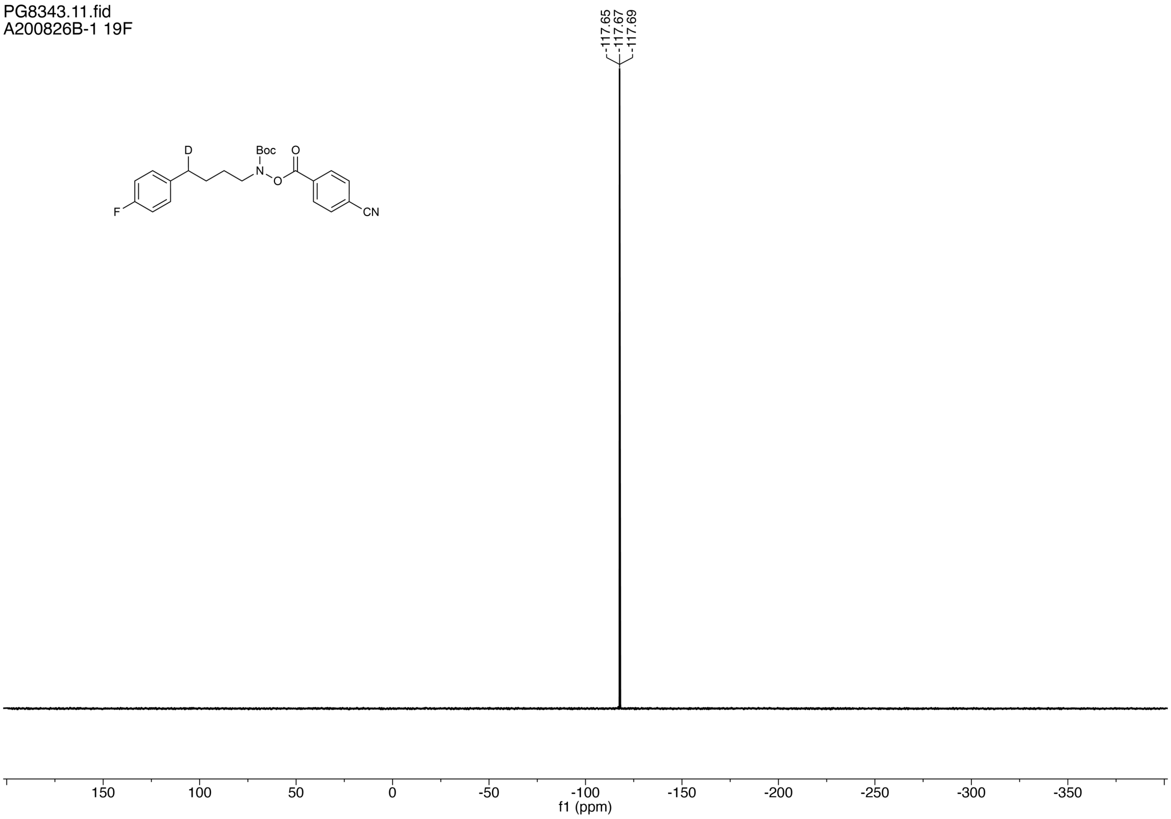

4-((((4-(4-Fluorophenyl)butyl-4-*d*)amino)oxy)carbonyl)benzonitrile (18):

^{1}H NMR (400 MHz, $CDCl_3$)

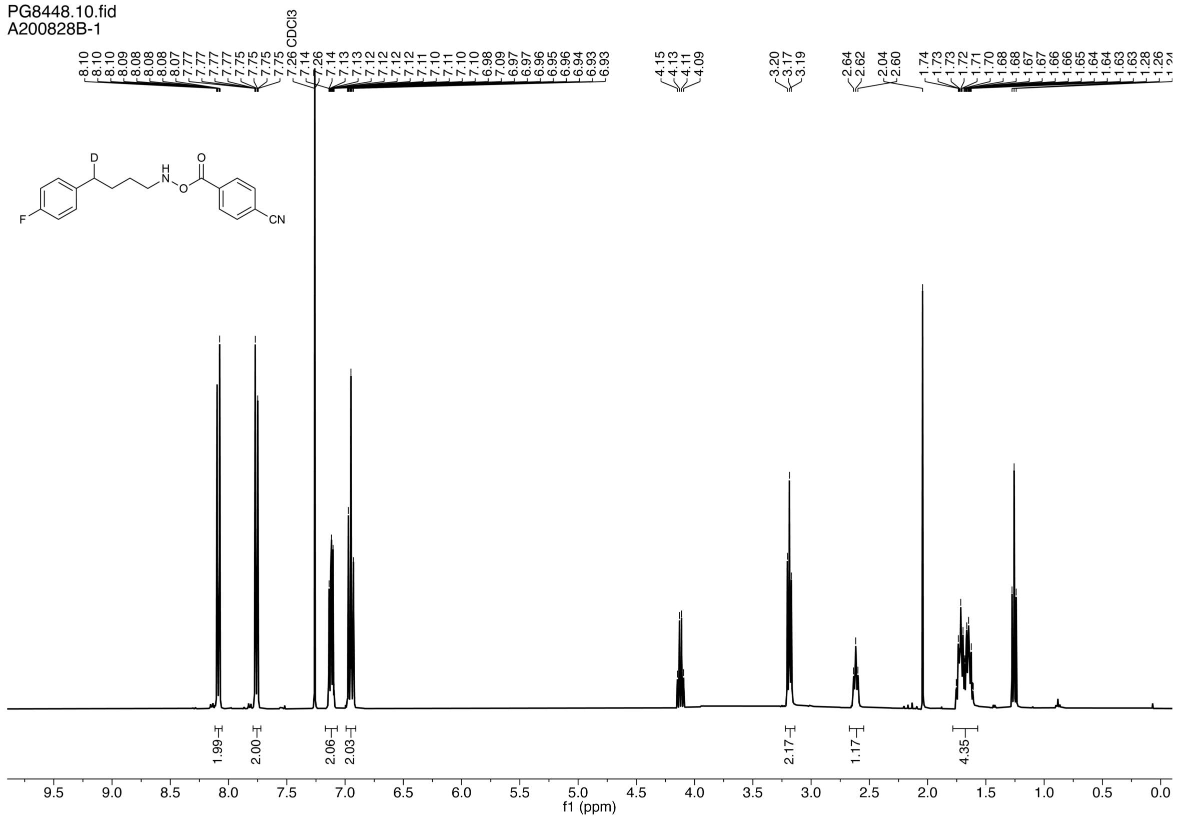

^{13}C NMR (100 MHz, $CDCl_3$)

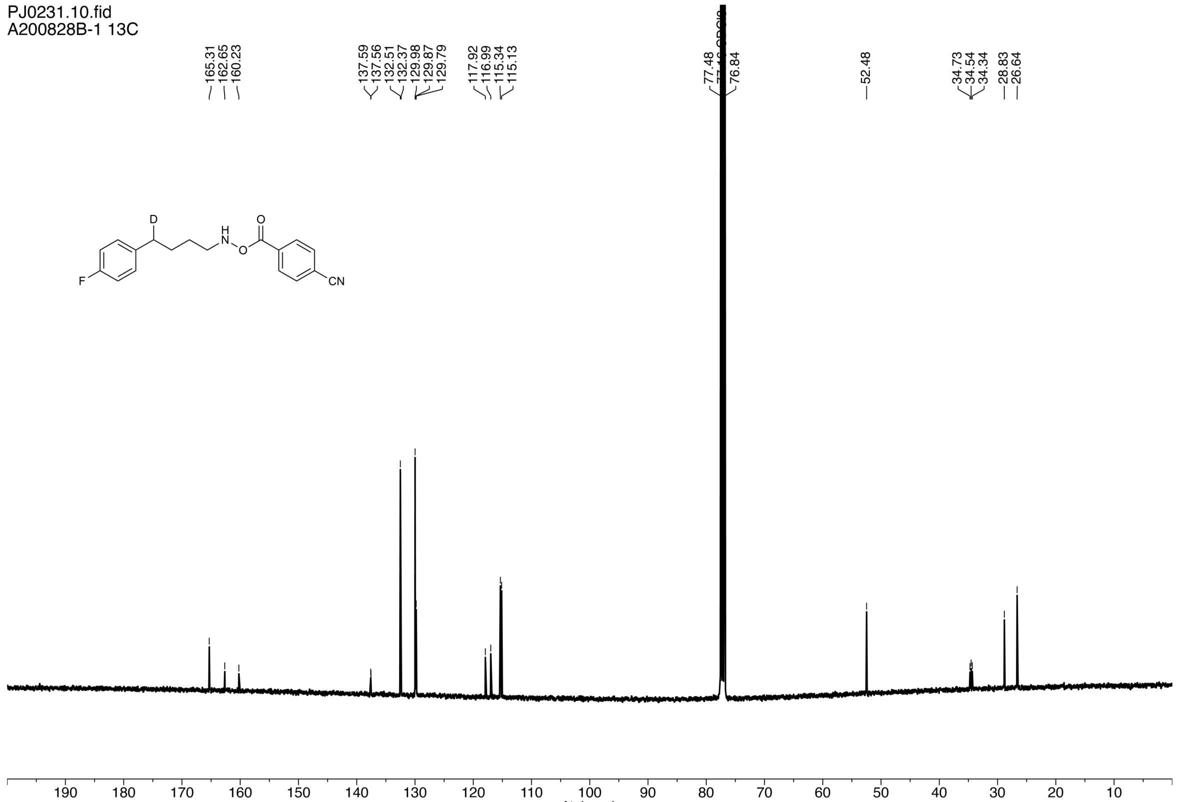

^{19}F NMR (376 MHz, $CDCl_3$)

PG8448.11.fid
A200828B-1 19F

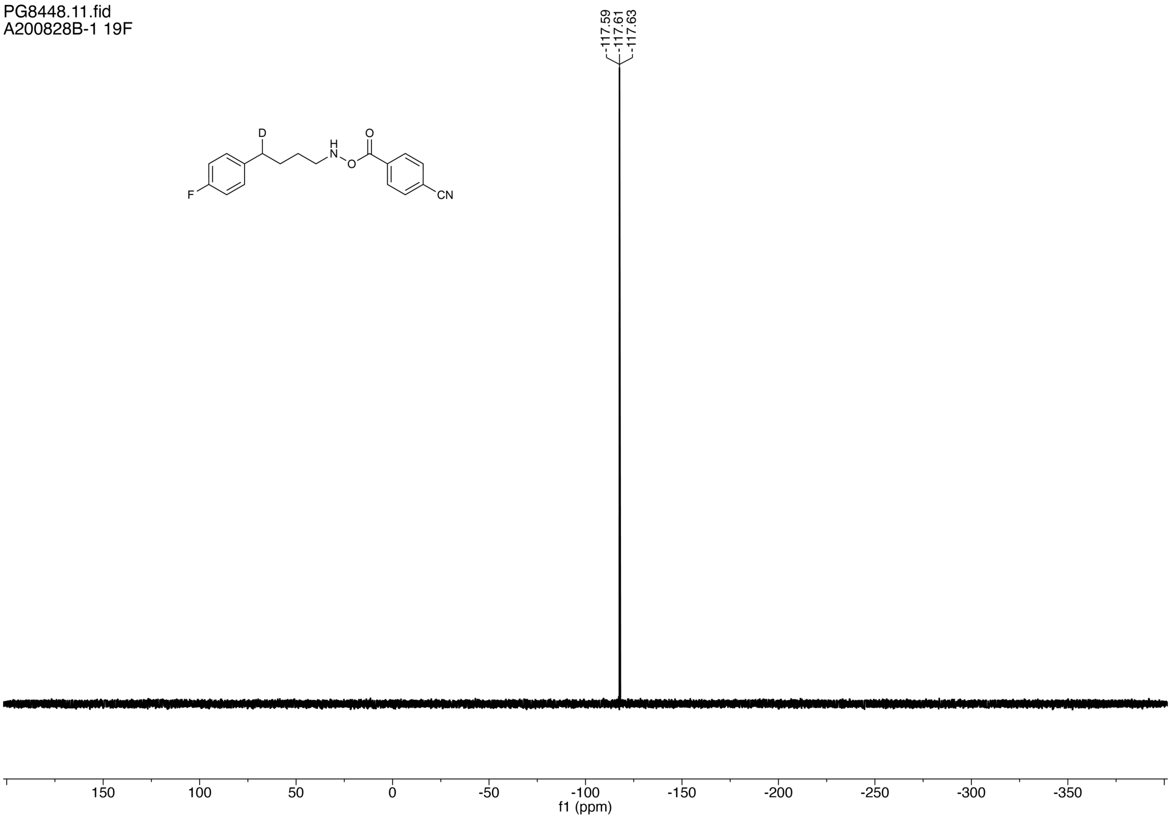

4-Isobutyl-2-phenyl-1-tosylpyrrolidine (Ts-12a):

^{1}H NMR (400 MHz, $CDCl_3$)

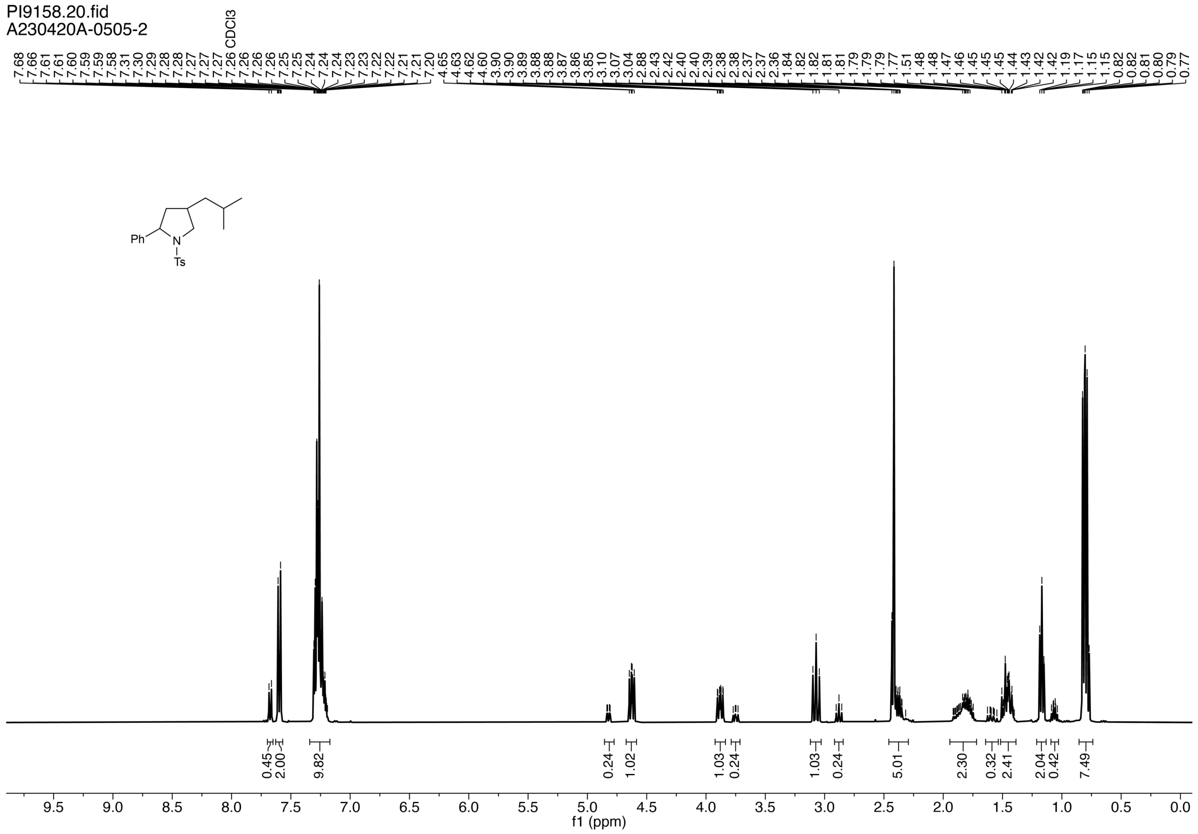

^{13}C NMR (100 MHz, $CDCl_3$)

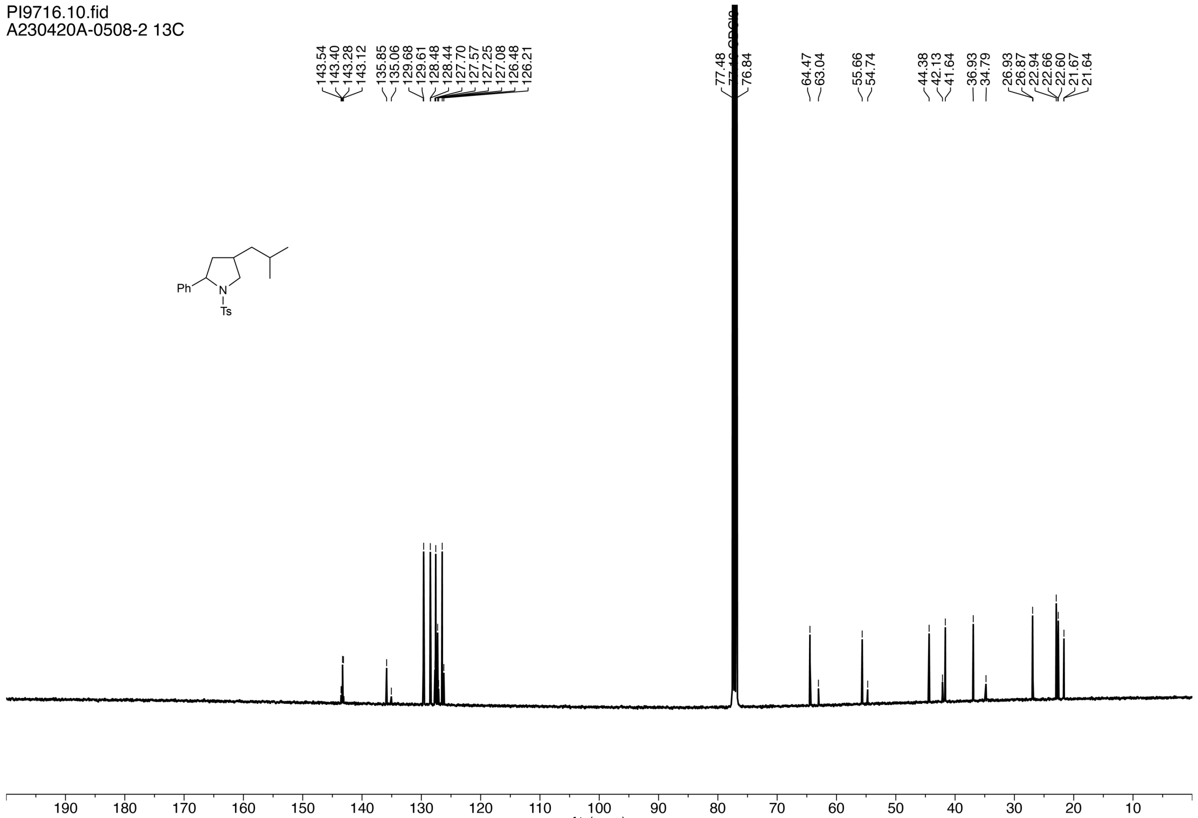

COSY:

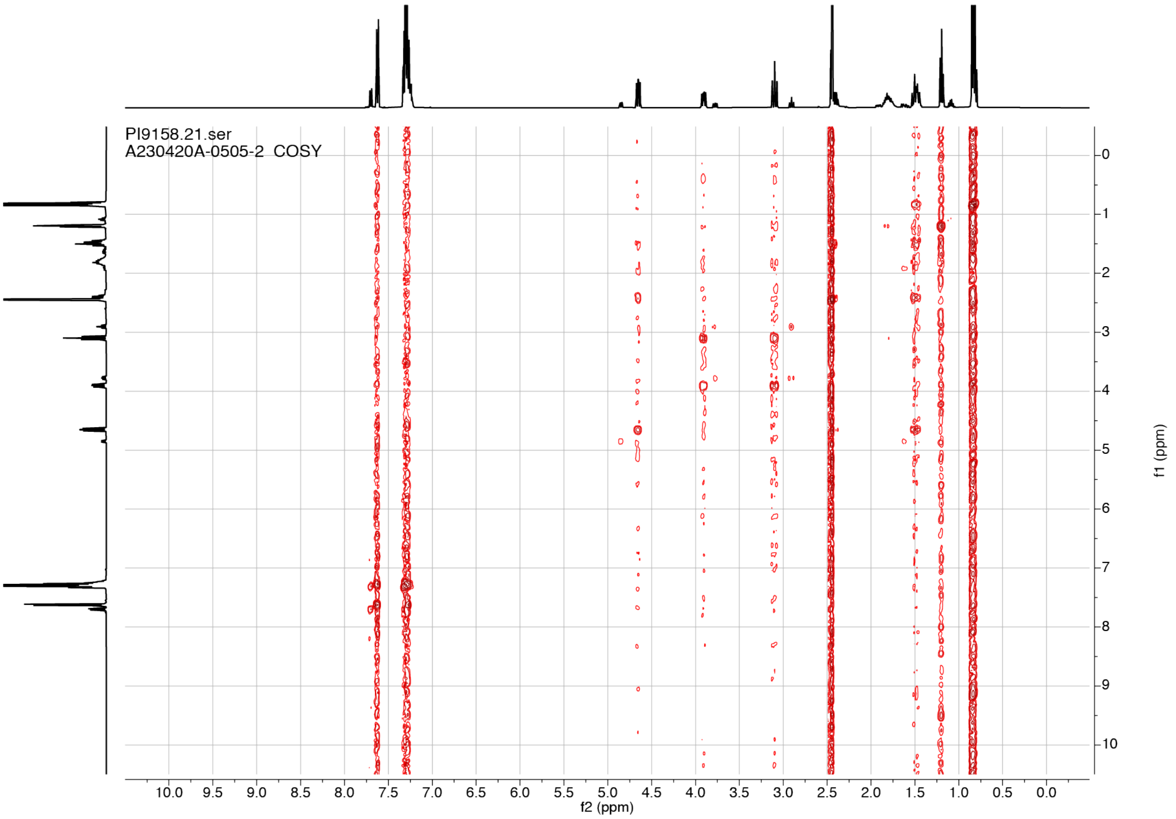
PI9158.21.ser
A230420A-0505-2 COSY
f2 (ppm)
f1 (ppm)

NOE

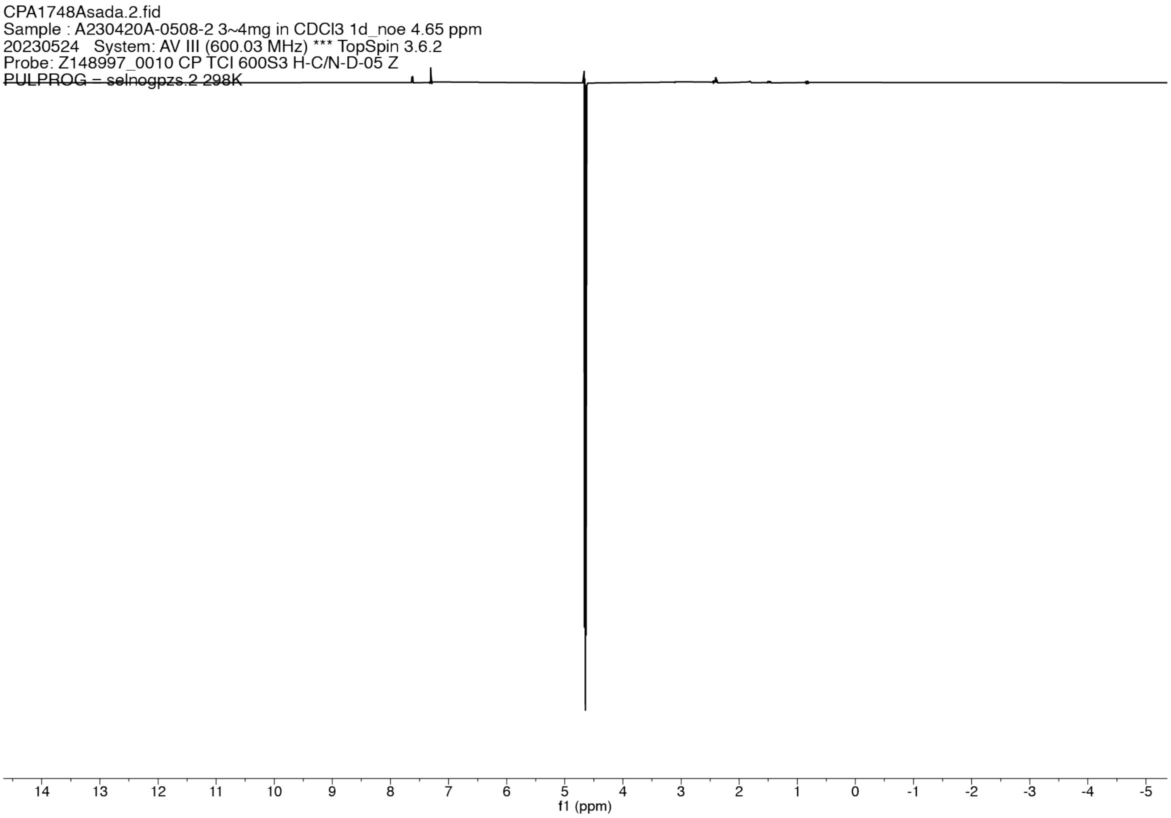
CPA1748Asada.2.fid
Sample : A230420A-0508-2 3~4mg in CDCl3 1d_noe 4.65 ppm
20230524 System: AV III (600.03 MHz) *** TopSpin 3.6.2
Probe: Z148997_0010 CP TCI 600S3 H-C/N-D-05 Z
PULPROG = selnogpzs.2 298K
f1 (ppm)

CPA1748Asada.3.fid
Sample : A230420A-0508-2 3~4mg in CDCl3 1d_noe 4.845 ppm
20230524 System: AV III (600.03 MHz) *** TopSpin 3.6.2
Probe: Z148997_0010 CP TCI 600S3 H-C/N-D-05 Z
PULPROG = selnogpzs.2 298K

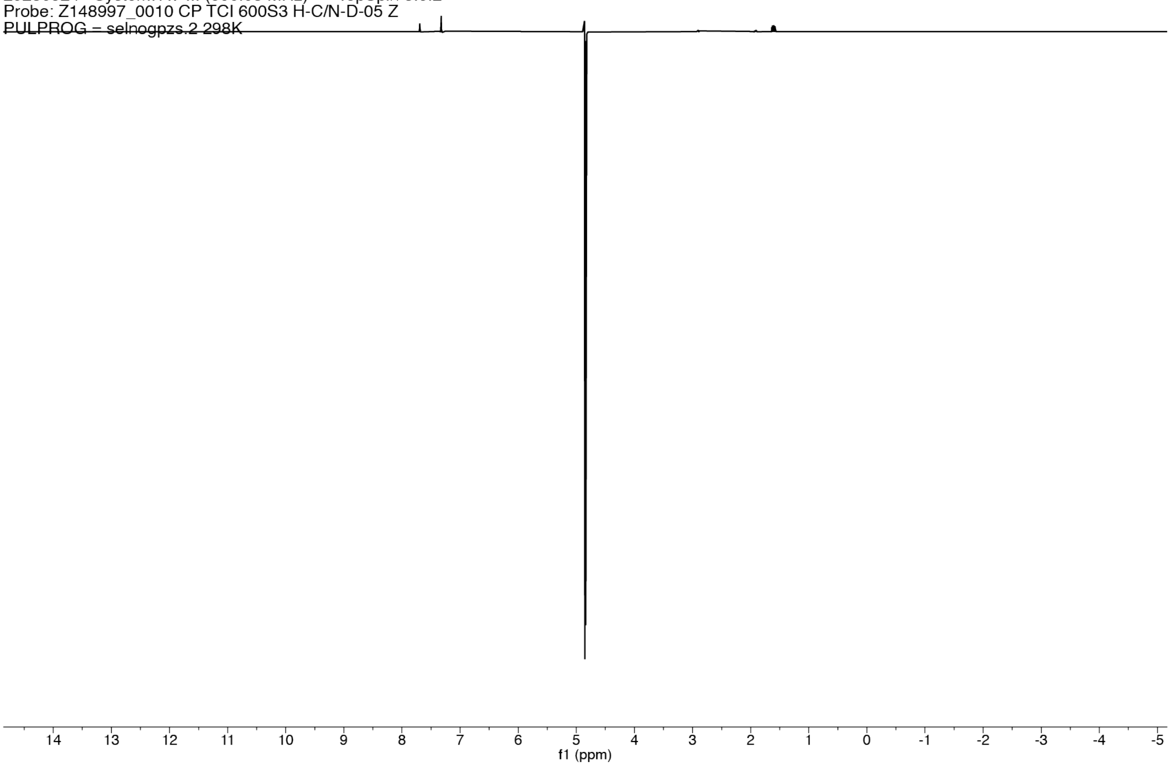

CPA1748Asada.4.fid
Sample : A230420A-0508-2 3~4mg in CDCl3 1d_noe 4.845 ppm
20230524 System: AV III (600.03 MHz) *** TopSpin 3.6.2
Probe: Z148997_0010 CP TCI 600S3 H-C/N-D-05 Z
PULPROG = selnogpzs.2 298K

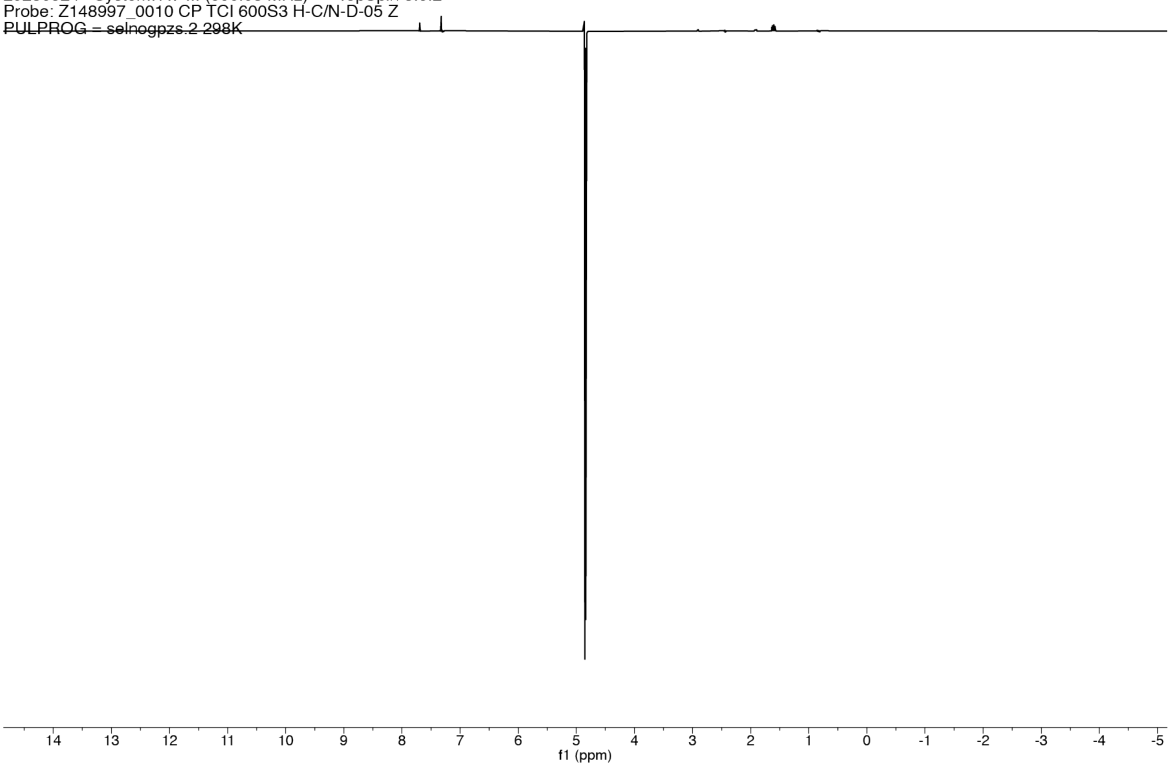

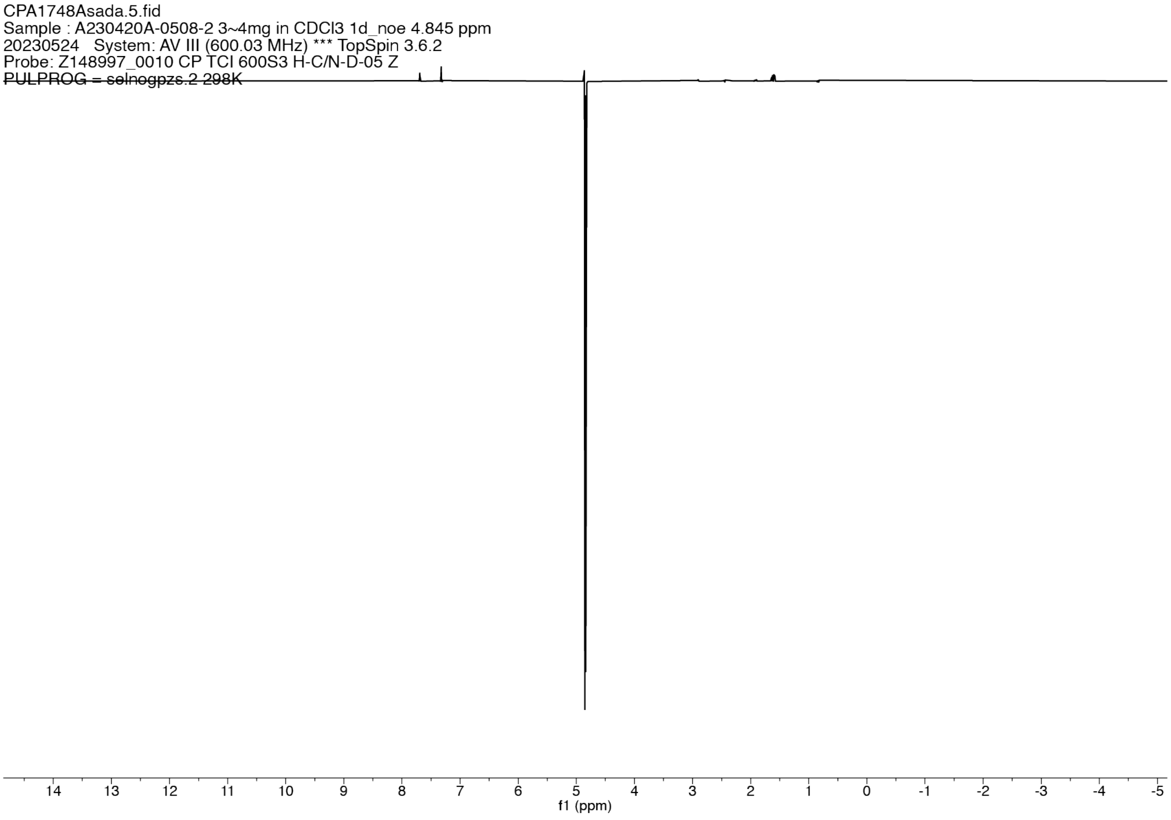
CPA1748Asada.5.fid
Sample : A230420A-0508-2 3~4mg in CDCl3 1d_noe 4.845 ppm
20230524 System: AV III (600.03 MHz) *** TopSpin 3.6.2
Probe: Z148997_0010 CP TCI 600S3 H-C/N-D-05 Z
PULPROG = selnogpzs.2 298K
14 13 12 11 10 9 8 7 6 5 4 3 2 1 0 -1 -2 -3 -4 -5
f1 (ppm)

2-Phenyl-4-propyl-1-tosylpyrrolidine (Ts-12b):

[1]**H NMR** (400 MHz, $CDCl_3$)

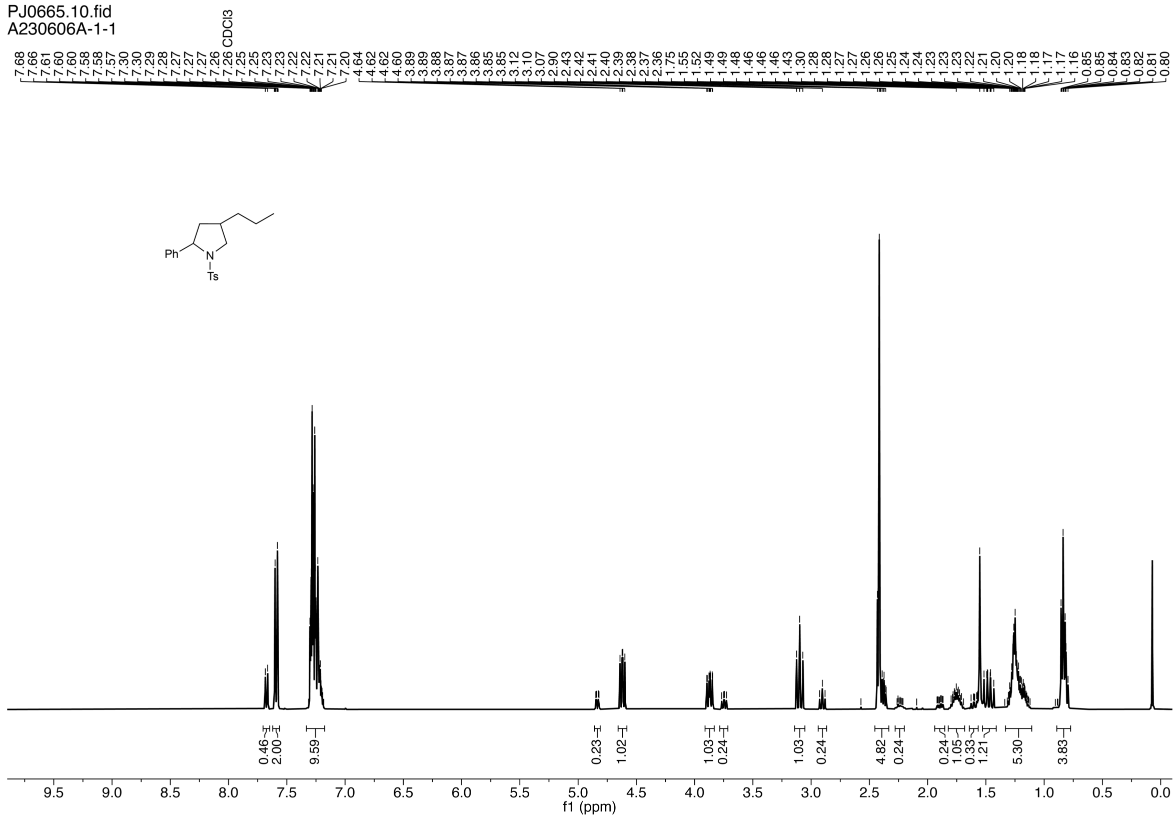

[13]**C NMR** (100 MHz, $CDCl_3$)

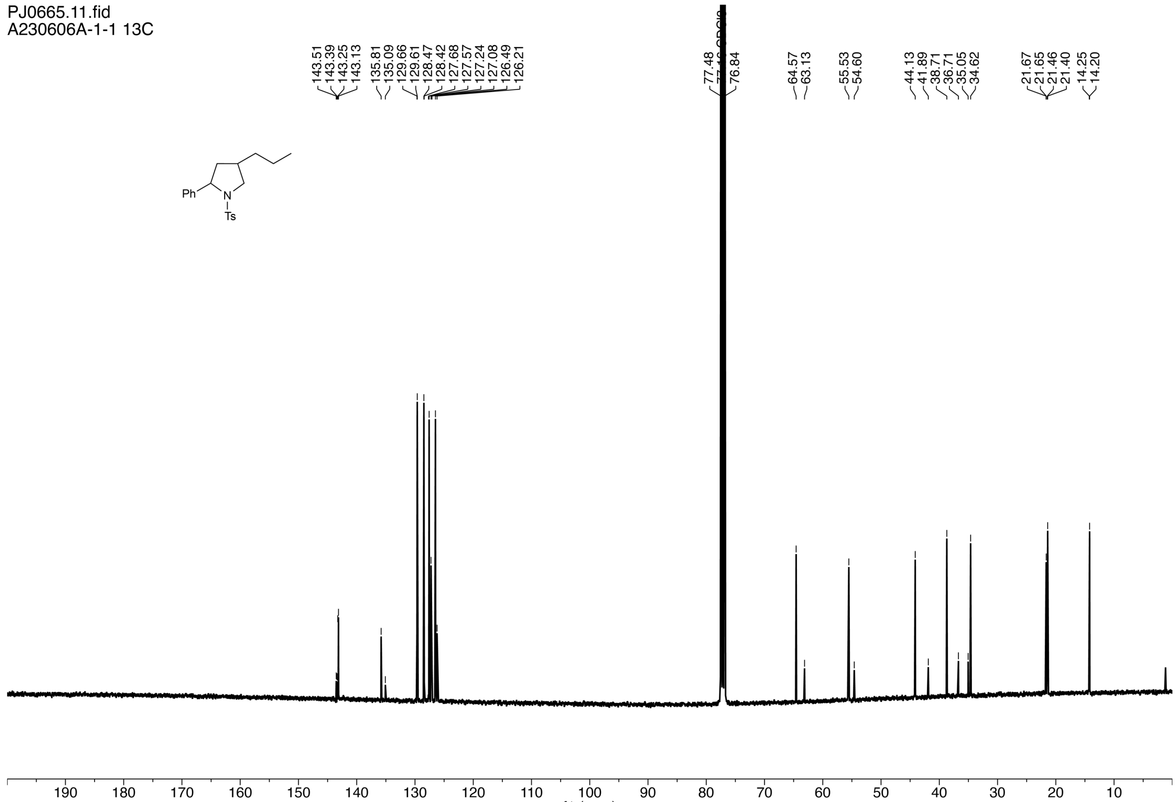

4-Ethyl-2-phenyl-1-tosylpyrrolidine (Ts-12c):

^{1}H NMR (400 MHz, $CDCl_3$)

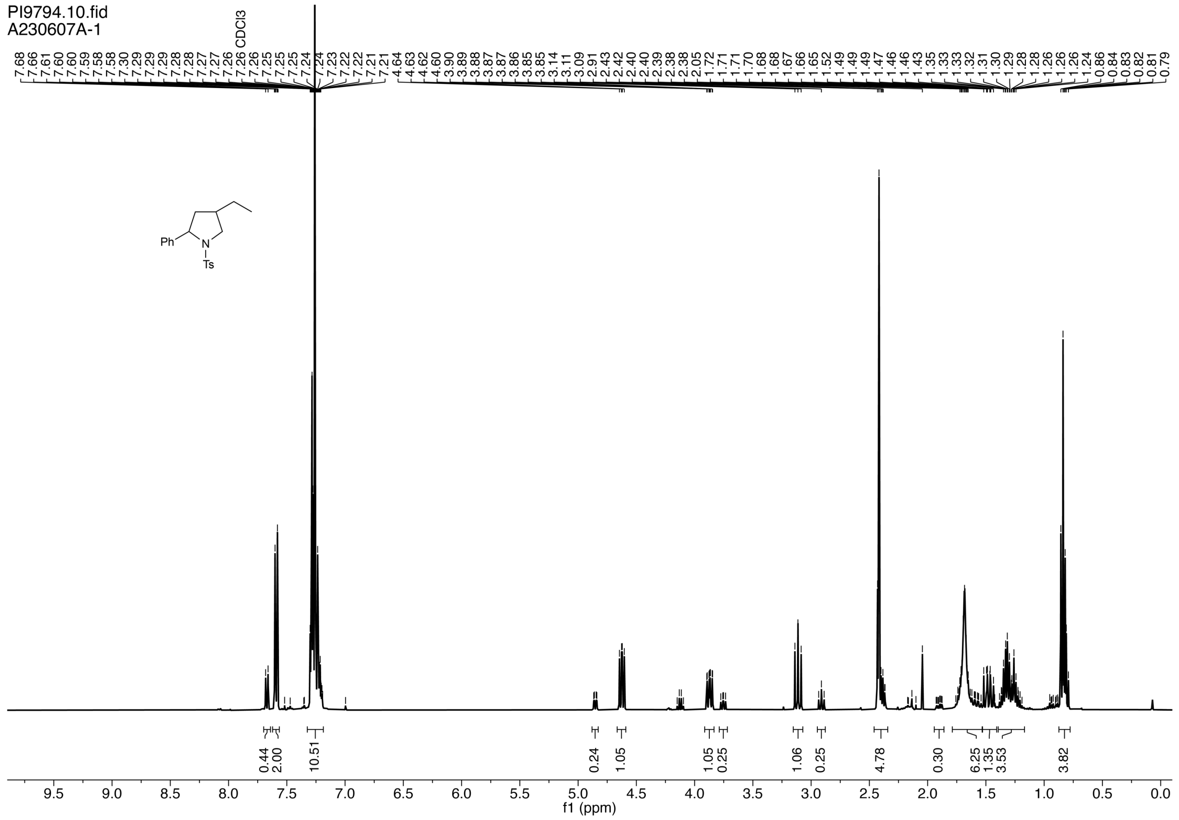

^{13}C NMR (100 MHz, $CDCl_3$)

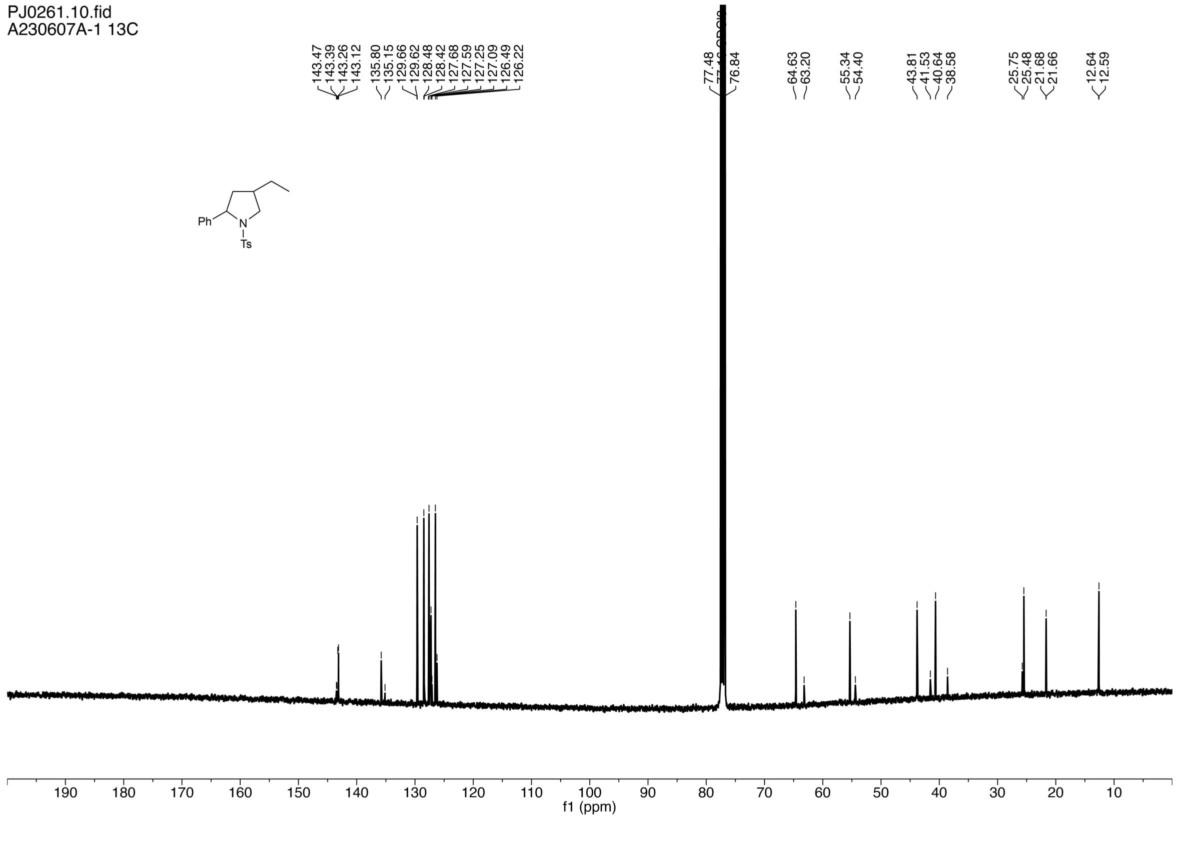

2,2-Dimethyl-4-phenethyl-1-tosylpyrrolidine (Ts-13a):

^{1}H NMR (400 MHz, $CDCl_3$)

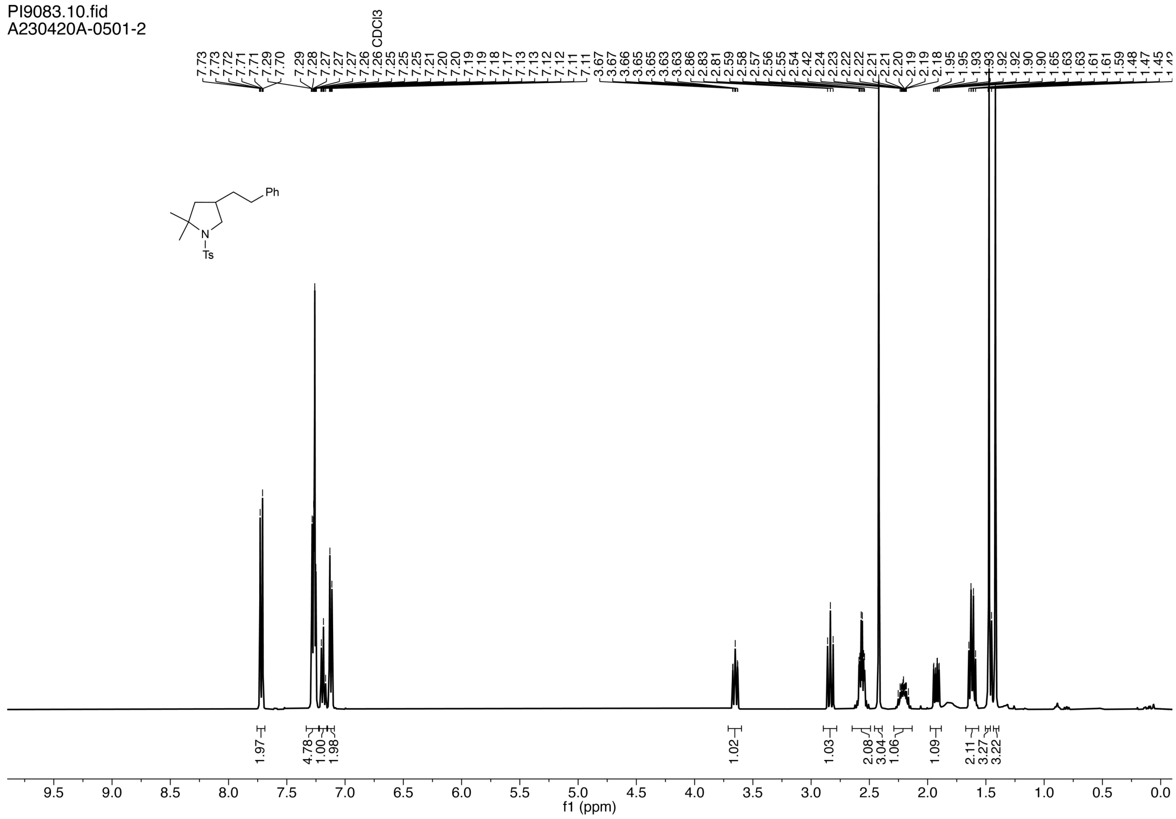

^{13}C NMR (100 MHz, $CDCl_3$)

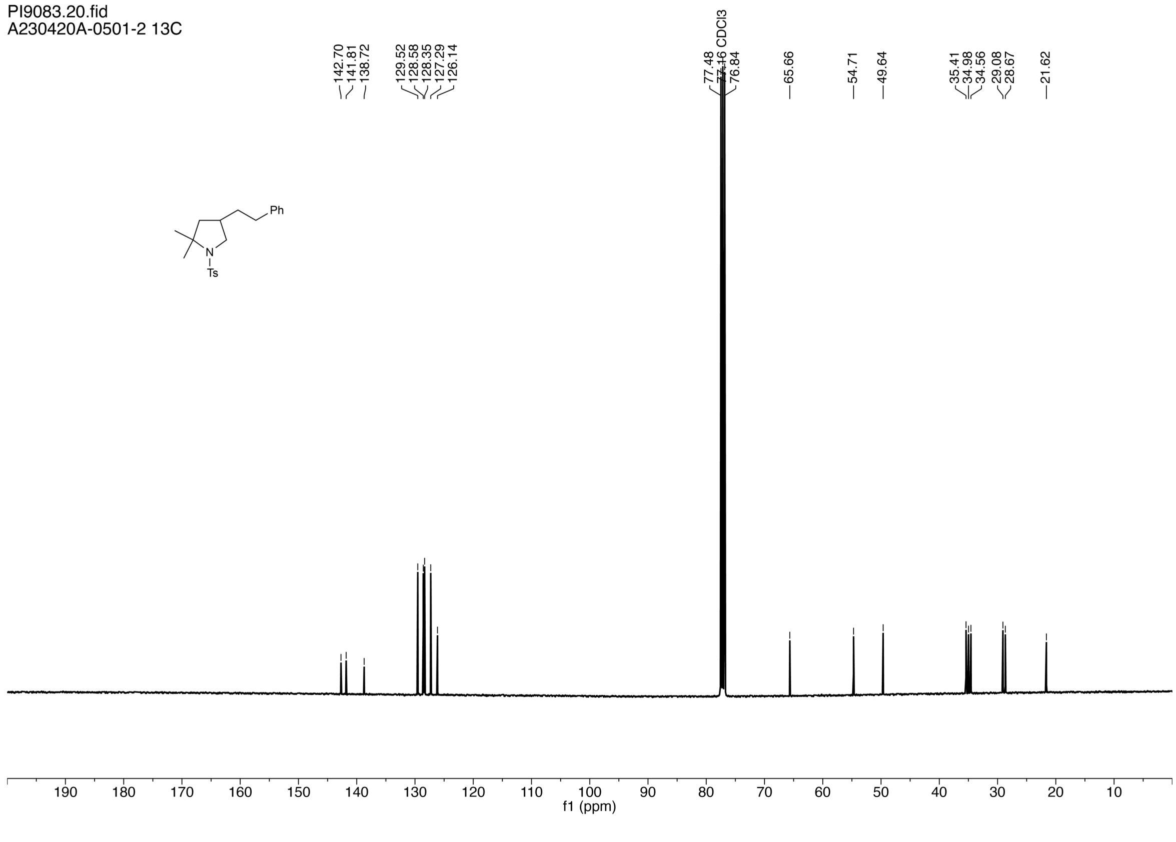

Supplement: Supplementary file 1 [file DataSheet1.PDF]
